# Selective One-Pot Multicomponent Synthesis of *N*-Substituted 2,3,5-Functionalized 3-Cyanopyrroles via the Reaction between α-Hydroxyketones, Oxoacetonitriles, and Primary Amines

**DOI:** 10.3390/molecules27165285

**Published:** 2022-08-18

**Authors:** Mengxin Xia, Ziad Moussa, Zaher M. A. Judeh

**Affiliations:** 1 School of Chemical and Biomedical Engineering, Nanyang Technological University, 62 Nanyang Drive, N1.2–B1-14, Singapore 637459, Singapore; 2 Department of Chemistry, College of Science, United Arab Emirates University, Al Ain P.O. Box 15551, United Arab Emirates

**Keywords:** pyrroles, carbohydrates, sustainable synthesis, three-component reactions, one-pot reactions

## Abstract

A one-step, three-component reaction between α-hydroxyketones, oxoacetonitriles, and primary amines gives *N*-substituted 2,3,5-functionalized 3-cyanopyrroles with complete selectivity in up to 90% isolated yields. The reaction worked on a wide substrate scope under mild reaction conditions (AcOH as a catalyst, EtOH, 70 °C, 3 h). The reaction proceeded with very high atom efficiency as water is the only molecule lost during the reaction. The practicality of the reaction was demonstrated on a large gram scale. The structures of the 3-cyanopyrroles were confirmed by single-crystal X-ray diffraction and NMR; this work provides a general and practical entry to pyrrole scaffolds suitably decorated for the synthesis of various bioactive pyrroles in a concise manner.

## 1. Introduction

**Figure 1:**
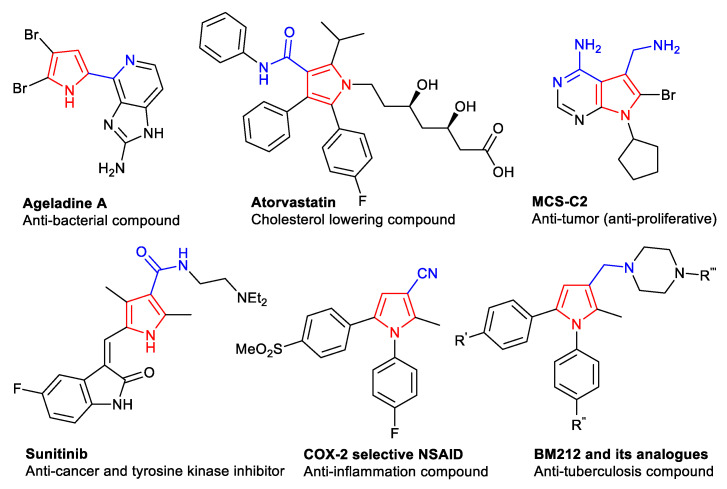
Examples of bioactive pyrrole-based compounds having a -CXN moiety (in blue).

Pyrroles are a class of nitrogen heterocycles with important applications in the pharmaceutical and material science industries [1,2,3]. Owing to their diverse bioactivities such as anti-inflammatory [4], anti-bacterial [5], anti-tumor [6], and anti-oxidative bioactivities [7], several pyrrole-based drugs have been successfully marketed to treat various conditions (Figure 1). Additionally, many pyrrole-based drug candidates have shown great promise in treating various conditions (Figure 1). Therefore, continuous efforts have been invested in designing simple, efficient, and sustainable routes to synthesize suitably functionalized pyrroles with specific “handles” for easy transformation to the final dug compounds. In this regard, special attention is given to synthesizing 3-cyanopyrroles since the cyano handle can easily be converted to -CXN functionality (e.g., X = H, H_2_, O, or simply nothing); this -CXN functionality appears in many drugs and drug candidates (Figure 1) [5,8,9,10,11,12].

**Figure 2:**
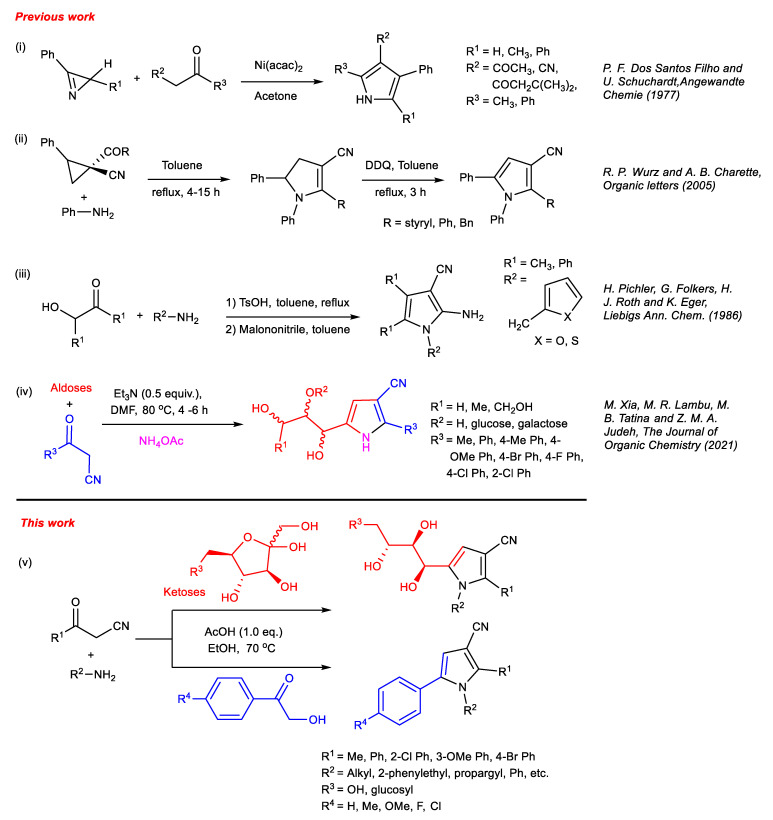
Previous and proposed work for the synthesis of 3-cyanopyrroles.

Previous efforts to synthesize 3-cyanopyrroles include (i) the nickel(II) bis(acetylacetonate)-catalyzed reaction between azirines and active methylene compounds reported in 1977 (Figure 2, (i)) [13]; (ii) the reaction between 1-nitro-1-cyclopropyl ketones and primary amines followed by oxidizing the dihydropyrrole products using 2,3-dichloro-5,6-dicyano-1,4-benzoquinone (DDQ) to give the desired 3-cyanopyrroles (Figure 2, (ii) [14]; and (iii)) the reaction between α-hydroxyketones, malononitrile and primary amines (Figure 2, (iii)) [15]; however, the first two reactions require extra steps to prepare the complex starting materials, use metals to catalyze the reactions, and are multistep syntheses. The last reaction works when the substituents at the α-hydroxyketones are similar (R^1^ and R^1,^ both either methyl or phenyl, Figure 1, (iii)) and the starting material itself is not considered sustainable. In this reaction, when the substituents are different, several products are formed, which reduces the yields and complicates separation [16]; moreover, these reactions are conducted at high temperatures > 90 °C in toluene. Additionally, other inefficient multistep reactions introduced the cyano handle on a preformed pyrrole and usually used heavy metals or toxic reagents [17]. These disadvantages encourage the search for a more robust, practical, and sustainable methodology.

Earlier, we developed a cascade reaction between aldoses, oxoacetonitriles, and NH_4_OAc to synthesize *N*-unsubstituted 3-cyanopyrroles (Figure 2, (iv)) [18]. Based on our previous experience [18,19], we envisioned that ketoses should also be ideal substrates for the synthesis of functionalized 3-cyanopyrroles (Figure 1, (iii)). Ketoses are cheap, sustainable materials and can introduce chirality in the pyrrole framework; moreover, their polyhydroxyalkyl chain can easily be converted into several functional groups; it is also beneficial to establish general reaction conditions that work for other α-hydroxyketones (ketoses are, in fact, masked α-hydroxyketones) such as phenacyl alcohols to add diversity to the substituents on the core 3-cyanopyrrole structure (Figure 1, (v)). Another important objective in this work is to achieve complete selectivity while using differently substituted α-hydroxyketones (R^1^ ≠ R^1^, Figure 1, (iii)) which has proven to be challenging in previous reports [16].

Herein, we report an AcOH-catalyzed selective one-pot three-component reaction between α-hydroxyketones (ketoses and phenacyl alcohols), oxoacetonitriles, and primary amines to synthesize densely functionalized 3-cyanopyrroles; this reaction gave the desired 3-cyanopyrroles in 53–90% yields and worked successfully on a gram scale. The structures of the 3-cyanopyrroles were confirmed using single-crystal X-ray analysis, and a plausible reaction mechanism was also provided.

## 2. Results and Discussion

### 2.1. Reaction Optimization

We started the investigation by stirring D-(+)-fructose **1** (1 mmol, 1.0 eq.), benzoylacetonitrile **2** (1.0 eq.), and benzylamine **3** (1.1 eq.) in one pot in the presence of AcOH (1.0 eq.) in EtOH (3 mL) at 70 °C for 3 h (Table 1, entry 1). The reaction gave 1-benzyl-2-phenyl-5-((1*R*,2*S*,3*R*)-1,2,3,4-tetrahydroxybutyl)-1*H*-pyrrole-3-carbonitrile **1a** (*N*-substituted 2,3,5-functionalized 3-cyanopyrrole) in 77% yield (Table 1, entry 1). Under these conditions, complete selectivity was obtained and *N*-substituted 2,3,5-functionalized 3-cyanopyrrole **1a** was the only product recovered. Attempts to increase the yield of **1a** by increasing the temperature or reaction time were unsuccessful and led to more decomposition products indicated by a darkening of the reaction mixture. Next, several acidic and basic catalysts were examined. Except for ZnCl_2_, which gave **1a** in 46% yield, other acids, including *p*-toluenesulfonic acid (PTSA) and camphor sulfonic acid (CSA), and bases including triethylamine (Et_3_N), sodium hydroxide (NaOH), potassium carbonate (K_2_CO_3_) and sodium methoxide (NaOMe) did not give the desired product, and the starting materials were recovered back (Table 1, entry 2–8). Changing EtOH solvent to acetonitrile (ACN) or dimethylformamide (DMF) did not improve the yield of **1a** (Table 1, entry 1 vs. 9 vs. 10). Further, attempts to reduce the equivalents of AcOH from 1 to 0.5 reduced the yield of **1a** dramatically (Table 1, entry 1 vs. 11). Attempts to reduce reaction temperature to 40 °C gave **1a** in trace amount (Table 1, entry 12). Therefore, we concluded the optimum conditions to be 1 equivalent of AcOH in EtOH at 70 °C for 3 h (Table 1, entry 1).

### 2.2. Substrate Scope

Initially, the scope of the reaction was investigated by reacting different ketoses, including *D*-(+)-fructose **1** and isomaltulose (also known as palatinose) **4** with primary amines **3** and **5**–**12** and oxoacetonitriles **2** and **13**, (Table 2) under the optimized condition (Table 1, entry 1). The selected ketoses were representative of mono- and disaccharides, the amines were representative of primary, secondary, unsaturated, aliphatic, aromatic, and heterocyclic amines, while the oxoacetonitriles were representative of aliphatic and aromatic nitriles. Ketoses **1** and **4** reacted selectively to give the *N*-substituted 2,3,5-functionalized 3-cyanopyrroles **1a**–**k** and **4a**–**c** in 55–86% isolated yields. In particular, 3-cyanopyrroles **4a**–**c** are interesting structures since they combine both a pyrrole moiety and a sugar moiety and are water-soluble [20]. The yield of the 3-cyanopyrroles **1a–k** and **4a–c** was affected by the type of sugar and the type of the primary amine. Hence, *D*-(+)-fructose/amines provide higher yields than isomaltulose/amines (Table 2, entries 1–6 vs. 12–14), and aliphatic amines provided higher yields than aromatic amines due to their higher nucleophilicity (Table 2, entries 1–6 vs. 7–9). All the 3-cyanopyrroles **1a**–**k** and **4a**–**c** were purified by column chromatography using a mixture of MeOH/DCM (5–10% MeOH/DCM for **1a**–**k**, and 15–20% MeOH/DCM for **4a**–**c**). The high polarity of the 3-cyanopyrroles is attributed to the extensive hydrogen bonding in the polyhydroxyalkyl chains.

Next, the reactivity of phenacyl alcohols **14**–**17** as the α-hydroxyketone was tested under the optimum conditions (Table 1, entry 1) to further extend the scope of this reaction and examine the selectivity. Additionally, phenacyl alcohols would also give needed diversity to the substituents on the 3-cyanopyrrole core. The reaction between phenacyl alcohols **14**–**17** [21] oxoacetonitriles **2**, **13**, and **18**–**20**, and primary amines **3**, **5**, **6**, **10**, **11**, and **21** selectively gave the desired *N*-substituted 2,3,5-functionalized 3-cyanopyrroles **14a**–**e**, **15a**, **16a**–**b**, and **17a**–**b** in excellent 70–90% isolated yields (Table 3). Again, aromatic amines generally gave lower yields than aliphatic amines due to their lower nucleophilicity. 3-Cyanopyrroles **14a**–**e**, **15a**, **16a**-**b**, and **17a**–**b** were purified using silica gel column chromatography using a mixture of 5–35% EtOAc/Hexane. These 3-cyanopyrroles showed the expected lower polarity on silica gel TLC and column chromatography compared to 3-cyanopyrroles **1a**–**k** and **4a**–**c** due to lower hydrogen bonding.

**Figure 3:**
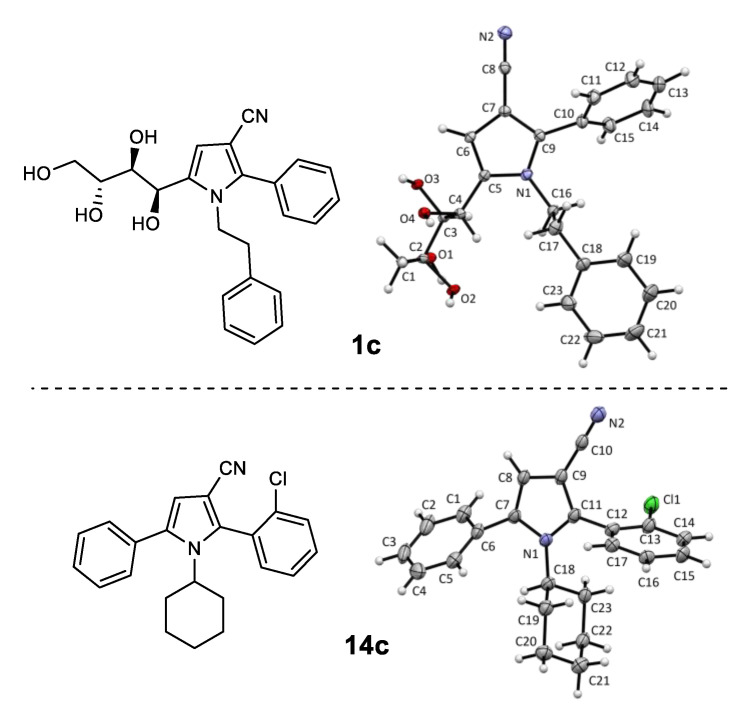
Single-crystal XRD of 3-cyanopyrroles **1c** and **14c** (displacement ellipsoids are drawn at the 50% probability level).

The structures of 3-cyanopyrroles **1c** and **14c** and the substitution pattern on the pyrrole ring were unambiguously confirmed by single-crystal X-ray diffraction analysis. The crystal structures of 3-cyanopyrroles **1c** (CCDC 2154839) and **14c** (CCDC 2154829) (Figure 3) combined with the NMR data (see Supplementary Materials) also confirmed the structures of pyrroles **14a**–**e, 15a**, **16a**–**b**, and **17a**–**b**.

### 2.3. Large-Scale Reactions

**Scheme 1:**
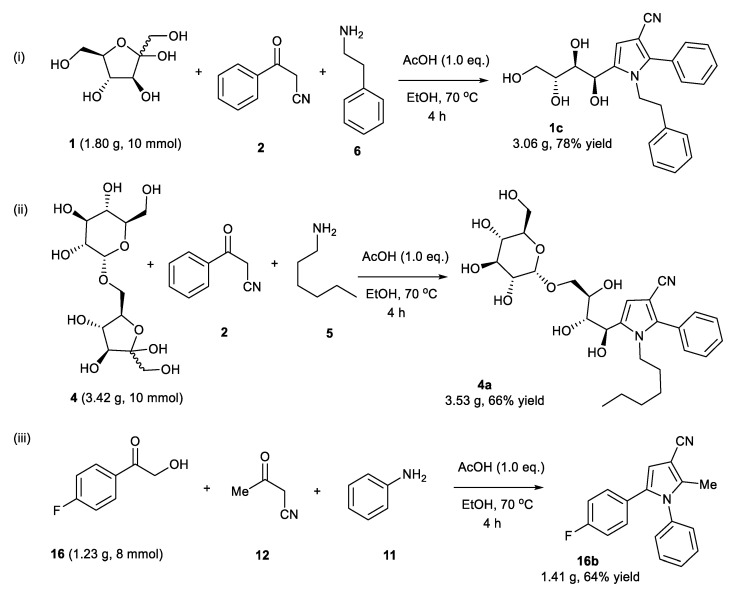
Large-scale reactions for the synthesis of 3-cyanopyrroles **1c**, **4a**, and **16b**.

To examine the practicality of this one-step synthesis, the reactions were conducted using 8–10 mmol of the α-hydroxyketones. *D*-(+)-fructose **1** (1.80 g, 10 mmol), benzoylacetonitrile **2,** and benzylamine **3** reacted to give 3-cyanopyrrole **1c** in 78% isolated yield (Scheme 1, (i)). Similarly, isomaltulose **4** (3.42 g, 10 mmol), benzoylacetonitrile **2,** and hexylamine **5** reacted to give 3-cyanopyrrole **4a** in 66% isolated yield (Scheme 1, (ii)). Additionally, the reaction between 1-(4-fluorophenyl)-2-hydroxyethan-1-one **16** (1.23 g, 8 mmol), 3-oxobutanenitrile **13** and aniline **11** gave 3-cyanopyrrole **16b** in 64% isolated yield (Scheme 1, (iii)). All large-scale reactions worked smoothly and selectively to give the desired 3-cyanopyrroles, which demonstrated the potential of this route even on a larger scale.

### 2.4. Structural Modification of the Synthesized Pyrroles

**Scheme 2:**
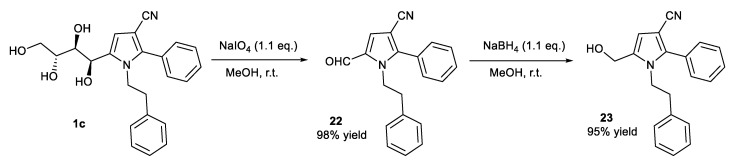
Modification of the polyhydroxyalkyl chain of 3-cyanopyrroles **1c** to 3-cyanopyrroles **22** and **23**.

The polyhydroxyalkyl chains of 3-cyanopyrroles **1a**–**k** are useful handles for easy modifications into other functional groups. For example, the polyhydroxyalkyl chain of pyrrole **1c** was oxidized effectively using sodium periodate to an aldehyde moiety, thus generating 5-formyl 3-cyanopyrroles **22** in 98% yield (Scheme 2) [22,23]. Further, the reduction of the formyl group of **22** using sodium borohydride gave 5-hydroxymethyl 3-cyanopyrroles **23** in 95% yield (Scheme 2) [24]. Both the aldehyde and hydroxyl groups can be used for homologation reactions or transformations to other functional groups. The 5-formyl and 5-hydroxymethyl groups are important functional substitutions that enable further modifications of 3-cyanopyrroles to become bioactive molecules or natural products [25,26].

### 2.5. Mechanistic Considerations

**Figure 4:**
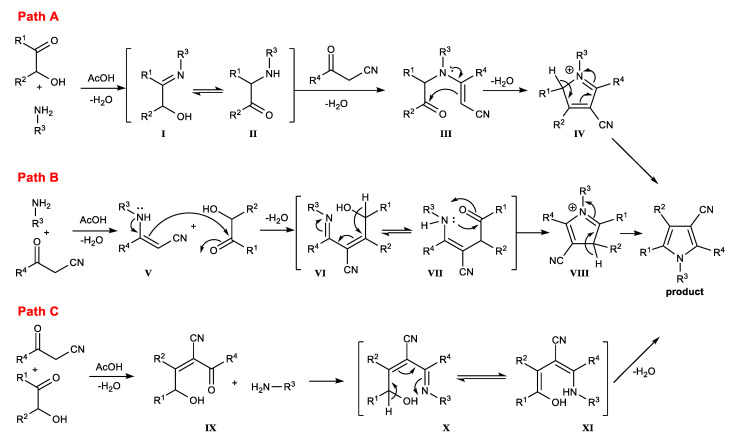
Proposed mechanism (paths A, B and C) for the formation of functionalized 3-cyanopyrroles.

Plausible reaction pathways are shown in Figure 4. In path A, the AcOH-catalyzed reaction between the α-hydroxyketones and amines gives the imine intermediate **I** which tautomerizes to intermediate **II**. The reaction of **II** with oxoacetonitriles gives enaminone intermediate **III**, which upon cyclization and dehydration, gives intermediate **IV**. Finally, the aromatization of **IV** gives the product (Figure 4, path A) [15,27]. In path B, oxoacetonitriles react first with primary amines to give the enamine intermediate **V,** which condenses with the α-hydroxyketones to give **VI**. Intermediate **VI** tautomerizes to **VII,** which upon intermolecular cyclization and loss of water gives **VIII**. Aromatization of **VIII** then gives the product (Figure 4, path B). In path C, the condensation between oxoacetonitriles and α-hydroxyketones gives intermediate **IX,** which upon condensation with the primary amines gives intermediate **X,** which isomerizes to intermediate **XI**. The product was formed from intermediate **XI** after intramolecular cyclization, dehydration, and aromatization (Figure 4, path C).

In all three pathways, the reaction proceeded with very high atom efficiency. Water was the only molecule lost during this three-component reaction.

## 3. Materials and Methods

All chemicals and AR grade solvents were obtained from Sigma-Aldrich (Saint Louis, MO, USA), Merck (Lebanon, NJ, USA) or Alfa Aesar (Tewksbury, MA, USA) and were used as received without further purification. IR spectra were recorded using Bruker MPA FT-IR machine (Karlsruhe, Germany). ^1^H NMR spectra were recorded at 300 MHz on a Bruker Avance DPX 300 or at 400 MHz Bruker Avance III 400 (BBFO 400) (Karlsruhe, Germany). ^13^C NMR spectra were recorded at 75.47 MHz on a Bruker Avance DPX 300 or at 101 MHz Bruker Avance III 400 (BBFO 400). Structural assignments were made with additional information from gCOSY, gHSQC, and gHMBC experiments. HRMS were measured using a hybrid Quadrupole Time-of-Flight (Q-TOF) (Waters Xevo G2-X2 MS) on Qstar XL MS/MS system (Milford, CT, USA). LCMS spectra were recorded using Agilent 6530 LCMS (Santa Clara, CA, USA). Single-crystal X-ray crystallographic analysis was done using Bruker D8 Quest (Karlsruhe, Germany). Analytical TLC was performed using Merck 60 F_254_ precoated silica gel plates (0.2 mm thickness) (Oakville, ON, Canada). The plates were visualized using UV radiation (254 nm) or stained in ceric ammonium sulfate solution with heating to detect the reaction spots. Flash chromatography was performed using Merck silica gel 60 (230–400 mesh) (Oakville, ON, Canada). The general procedure of phenacyl alcohols preparation, synthesis of 3-cyanopyrroles **1a**–**k**, **4a**–**c**, **14a**–**e, 15a**, **16a**–**b**, **17a**–**b** and compounds **22** and **23** are found below.

### 3.1. General Procedure—Preparation of Substituted Phenacyl Alcohols **14**–**17**

**Scheme 3:**
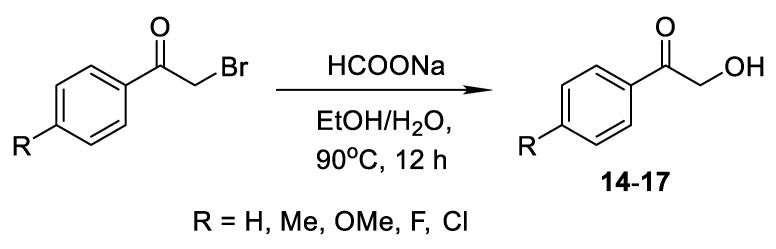
Preparation of Substituted Phenacyl Alcohols **14**–**17**.

A mixture of phenacyl bromide (20 mmol) and sodium formate (16 mmol) was stirred in ethanol/water (30 mL, EtOH:H_2_O = 9:1) at 90 °C for 12 hours. Upon completion of the reaction (TLC), the mixture was cooled to room temperature, and ethanol was evaporated under reduced pressure. The resulting concentrated mixture was diluted with water (8 mL) and extracted using ethyl acetate (10 mL × 3 times). The organic layers were dried over Mg_2_SO_4_, filtered, and evaporated to dryness under reduced pressure. The residual compound was purified using column chromatography using an EtOAc/Hexane mixture (1:4). This general procedure was used to prepare phenacyl alcohols **14**–**17** (Scheme 3).

### 3.2. General Procedure—Synthesis of 3-Cyanopyrroles **1a**–**k**, **4a**–**c**, **14a**–**e**, **15a**, **16a**–**b**, **17a**–**b**

AcOH (1.0 eq.) was added to a stirred solution of the α-hydroxyketones **1**, **4**, or **14**-**17** (1.0 eq.), oxoacetonitriles **2**, **13,** or **18**–**20** (1.0 eq.), and primary amines **3**, **5**–**12,** or **21** (1.1 eq.) in EtOH (3 mL) at room temperature. The reaction mixture was then heated at 70 °C for 3 h. Upon completion (TLC), the reaction mixture was evaporated to dryness under vacuum to produce the crude product as a foam. The foam was purified using silica gel column chromatography using eluent MeOH/CH_2_Cl_2_ (5:95 for *D*-(+)-fructose and 20:80 for isomaltulose) or EtOAc/Hexane (5–35:95–65) as eluants for phenacyl alcohols **14**–**17** to produce pure 3-cyanopyrroles. All reactions were conducted using 1.0 mmol of the substrates **1, 4**, and **14**–**17**. This general procedure was used to prepare 3-cyanopyrroles **1a**–**k**, **4a**–**c**, **14a**–**e, 15a**, **16a**–**b**, and **17a**–**b**.

1-Benzyl-2-Phenyl-5-((1*R*,2*S*,3*R*)-1,2,3,4-Tetrahydroxybutyl)-1*H*-Pyrrole-3-Arbonitrile (**1a**)

Obtained by general procedure 2 using *D*-(+)-fructose **1** (180 mg, 1.0 mmol), benzoylacetonitrile **2** (145 mg, 1.0 mmol), benzylamine **3** (120 µL, 1.1 mmol), and AcOH (57 µL, 1.0 mmol). The reaction produced the desired product **1a** (291 mg, 77%) as a hygroscopic pale-yellow solid. IR (KBr film) *ν*_max_ = 3416, 2221, 1637, 1398, 1077 cm^−1^. ^1^H NMR (300 MHz, MeOD) δ 7.43–7.38 (m, 3H), 7.34 (d, *J* = 5.2 Hz, 2H), 7.29–7.20 (m, 3H), 6.87 (d, *J* = 7.5 Hz, 2H), 6.79 (s, 1H), 5.40 (d, *J* = 12.0 Hz, 1H), 5.24 (d, *J* = 12.0 Hz, 1H), 4.88 (s, 1H), 3.79 – 3.69 (m, 3H), 3.61 (dd, *J* = 10.0, 4.3 Hz, 1H). ^13^C NMR (75 MHz, MeOD) δ 143.8, 138.9, 137.6, 131.0, 130.9, 130.4, 129.9, 129.9, 128.5, 126.9, 118.4, 112.0, 92.8, 74.4, 72.9, 66.1, 64.8, 49.4. DEPT135 ^13^C NMR (75 MHz, MeOD) δ 130.9, 130.4, 129.9 (2 × CH signals), 128.5, 126.9, 112.0, 74.4, 72.9, 66.1, 64.8, 49.4. HRMS (ESI-TOF) m/z: [M+H]^+^ calcd for C_22_H_23_N_2_O_4_^+^ 379.1658; found 379.1644. 

1-Hexyl-2-Phenyl-5-((1*R*,2*S*,3*R*)-1,2,3,4-Tetrahydroxybutyl)-1*H*-Pyrrole-3-Carbonitrile (**1b**)

Obtained by general procedure 2 using *D*-(+)-fructose **1** (180 mg, 1.0 mmol), benzoylacetonitrile **2** (145 mg, 1.0 mmol), hexylamine **5** (144 µL, 1.1 mmol), and AcOH (57 µL, 1.0 mmol). The reaction produced the desired product **1b** (320 mg, 86%) as a hygroscopic pale-yellow solid. IR (KBr film) *ν*_max_ = 3293, 2957, 2928, 2220, 1447, 1090 cm^−1^. ^1^H NMR (300 MHz, MeOD) δ 7.45–7.37 (m, 3H), 7.35–7.29 (m, 2H), 6.56 (s, 1H), 4.95 (s, 1H), 4.06–3.83 (m, 2H), 3.75–3.63 (m, 3H), 3.58–3.49 (m, 1H), 1.48–1.34 (m, 2H), 1.02-0.95 (m, 6H), 0.68 (t, *J* = 6.9 Hz, 3H). ^13^C NMR (75 MHz, MeOD) δ 144.5, 138.5, 133.1, 132.5, 131.7, 131.5, 119.9, 113.1, 93.8, 76.2, 74.4, 67.2, 66.3, 47.6, 33.6, 33.4, 28.6, 24.9, 15.7. DEPT135 ^13^C NMR (75 MHz, MeOD) δ 132.5, 131.7, 131.5, 113.1, 76.2, 74.4, 67.2, 66.3, 47.6, 33.6, 33.4, 28.6, 24.9, 15.7. HRMS (ESI-TOF) m/z: [M+Na]^+^ calcd for C_21_H_28_N_2_O_4_Na^+^ 395.1947; found 395.2000.

1-Phenethyl-2-Phenyl-5-((1*R*,2*S*,3*R*)-1,2,3,4-Tetrahydroxybutyl)-1*H*-Pyrrole-3-Carbonitrile (**1c**)

Obtained by general procedure 2 using *D*-(+)-fructose **1** (180 mg, 1.0 mmol), benzoylacetonitrile **2** (145 mg, 1.0 mmol), 2-phenylethylamine **6** (138 µL, 1.1 mmol), and AcOH (57 µL, 1.0 mmol). The reaction produced the desired product **1c** (329 mg, 84%) as a hygroscopic pale-yellow solid. IR (KBr film) *ν*_max_ = 3427, 3341, 2959, 2224, 1434, 1077 cm^−1^. ^1^H NMR (300 MHz, MeOD) δ 7.37–7.33 (m, 3H), 7.17–7.1 (m, 2H), 7.05-7.00 (m, 3H), 6.70 (dd, *J* = 6.6, 2.9 Hz, 2H), 6.58 (s, 1H), 4.99 (d, *J* = 1.4 Hz, 1H), 4.16–4.07 (m, 2H), 3.77–3.64 (m, 3H), 3.55 (dd, *J* = 10.2, 4.5 Hz, 1H), 2.69 (t, *J* = 7.5 Hz, 2H). ^13^C NMR (75 MHz, MeOD) δ 144.9, 140.5, 138.5, 132.8, 132.4, 131.6, 131.4, 131.3, 131.1, 129.2, 119.9, 113.2, 93.9, 76.3, 74.5, 67.3, 66.3, 49.3, 39.6. DEPT135 ^13^C NMR (75 MHz, MeOD) δ 132.4, 131.6, 131.4, 131.3, 131.1, 129.2, 113.2, 76.3, 74.5, 67.3, 66.3, 49.3, 39.6. HRMS (ESI-TOF) m/z: [M+H]^+^ calcd for C_23_H_25_N_2_O_4_^+^ 393.1814; found 393.1834.

1-(2-(1*H*-Imidazol-1-yl)ethyl)-2-Phenyl-5-((1*R*,2*S*,3*R*)-1,2,3,4-Tetrahydroxybutyl)-1*H*-Pyrrole-3-Carbonitrile (**1d**)

Obtained by general procedure 2 using *D*-(+)-fructose **1** (180 mg, 1.0 mmol), benzoylacetonitrile **2** (145 mg, 1.0 mmol), 2-(1*H*-imidazol-1-yl)ethanamine **7** (107 µL, 1.1 mmol), and AcOH (57 µL, 1.0 mmol). The reaction produced the desired product **1d** (279 mg, 73%) as a hygroscopic pale-yellow solid. IR (KBr film) *ν*_max_ = 3407, 2220, 1651, 1400, 1077 cm^−1^. ^1^H NMR (300 MHz, MeOD) δ 7.32 (dd, *J* = 6.2, 2.7 Hz, 3H), 7.05–6.95 (m, 3H), 6.69 (s, 1H), 6.61 (s, 1H), 6.44 (s, 1H), 4.88 (d, *J* = 2.5 Hz, 1H), 4.48–4.27 (m, 2H), 4.24–4.04 (m, 2H), 3.75–3.62 (m, 3H), 3.55 (dd, *J* = 10.8, 5.1 Hz, 1H). ^13^C NMR (75 MHz, MeOD) δ 145.4, 139.6, 138.7, 132.3, 131.8, 131.8, 131.4, 130.7, 122.2, 119.4, 113.8, 94.5, 76.4, 74.6, 67.4, 66.1, 49.3, 48.4. DEPT135 ^13^C NMR (75 MHz, MeOD) δ 139.6, 132.3, 131.8, 131.4, 130.7, 122.2, 113.8, 76.4, 74.6, 67.4, 66.1, 49.3, 48.4. HRMS (ESI-TOF) m/z: [M+H]^+^ calcd for C_22_H_22_BrN_2_O_3_^+^ 441.0814; found 441.0795.

2-Phenyl-1-(Prop-2-yn-1-yl)-5-((1*R*,2*S*,3*R*)-1,2,3,4-Tetrahydroxybutyl)-1*H*-Pyrrole-3-Carbonitrile (**1e**)

Obtained by general procedure 2 using *D*-(+)-fructose **1** (180 mg, 1.0 mmol), benzoylacetonitrile **2** (145 mg, 1.0 mmol), propargylamine **8** (70 µL, 1.1 mmol), and AcOH (57 µL, 1.0 mmol). The reaction produced the desired product **1e** (245 mg, 75%) as a hygroscopic pale-yellow solid. IR (KBr film) *ν*_max_ = 3290, 2219, 1699, 1400, 1089 cm^−1^. ^1^H NMR (300 MHz, MeOD) δ 7.49–7.39 (m, 5H), 6.58 (d, *J* = 0.4 Hz, 1H), 5.17–5.09 (m, 1H), 4.83 (dd, *J* = 18.2, 2.5 Hz, 1H), 4.60 (dd, *J* = 18.2, 2.5 Hz, 1H), 3.79–3.65 (m, 3H), 3.57 (dd, *J* = 10.7, 5.4 Hz, 1H), 2.78 (t, *J* = 2.5 Hz, 1H). ^13^C NMR (75 MHz, MeOD) δ 144.6, 138.5, 132.4, 132.0, 131.5, 119.6, 131.5, 94.1, 81.1, 76.9, 75.9, 75.5, 67.6, 66.3, 37.4. DEPT135 ^13^C NMR (75 MHz, MeOD) δ 132.4, 132.0, 131.5, 113.5, 75.9, 74.5, 67.5, 66.3, 37.4. HRMS (ESI-TOF) m/z: [M+H]^+^ calcd for C_22_H_22_BrN_2_O_3_^+^ 441.0814; found 441.0795.

1-(Naphthalen-1-ylmethyl)-2-Phenyl-5-((1*R*,2*S*,3*R*)-1,2,3,4-Tetrahydroxybutyl)-1*H*-Pyrrole-3-Carbonitrile (**1f**)

Obtained by general procedure 2 using *D*-(+)-fructose **1** (180 mg, 1.0 mmol), benzoylacetonitrile **2** (145 mg, 1.0 mmol), 1-naphthylmethylamine **9** (162 µL, 1.1 mmol), and AcOH (57 µL, 1.0 mmol). The reaction produced the desired product **1f** (291 mg, 68%) as a hygroscopic pale-yellow solid. IR (KBr film) *ν*_max_ = 3400, 2922, 2221, 1653, 1479, 1076 cm^−1^. ^1^H NMR (400 MHz, MeOD) δ 7.91–7.86 (m, 2H), 7.79 (d, *J* = 8.3 Hz, 1H), 7.53–7.48 (m, 2H), 7.42 – 7.38 (m, 1H), 7.34 (dd, *J* = 7.6, 1.8 Hz, 2H), 7.31 – 7.24 (m, 3H), 6.91 (s, 1H), 6.63 (d, *J* = 7.0 Hz, 1H), 5.79 (dd, *J* = 84.3, 17.7 Hz, 2H), 4.81 (d, *J* = 1.9 Hz, 1H), 3.81 (dd, *J* = 7.8, 2.1 Hz, 1H), 3.72 (dd, *J* = 10.9, 3.2 Hz, 1H), 3.67–3.62 (m, 1H), 3.58 (dd, *J* = 10.9, 5.7 Hz, 1H). ^13^C NMR (101 MHz, MeOD) δ 142.5, 136.4, 133.6, 133.2, 129.7, 129.3, 129.2, 129.0, 128.4 (2 × CH), 127.7, 126.2, 125.8, 125.2, 122.1, 121.9, 117.0, 110.8, 91.5, 73.1, 71.4, 64.7, 63.4, 46.3. DEPT135 ^13^C NMR (101 MHz, MeOD) δ 129.2, 129.0, 128.4 (2 × CH), 127.7, 126.2, 125.8, 125.2, 122.1, 121.9, 110.8, 73.1, 71.4, 64.7, 63.4, 46.3. HRMS (ESI-TOF) m/z: [M+Na]^+^ calcd for C_26_H_24_N_2_O_4_Na^+^ 451.1634; found 451.1610. 

1. -Cyclohexyl-2-Phenyl-5-((1*R*,2*S*,3*R*)-1,2,3,4-Tetrahydroxybutyl)-1*H*-Pyrrole-3-Carbonitrile (**1g**)

Obtained by general procedure 2 using *D*-(+)-fructose **1** (180 mg, 1.0 mmol), benzoylacetonitrile **2** (145 mg, 1.0 mmol), cyclohexylamine **10** (127 µL, 1.1 mmol), and AcOH (57 µL, 1.0 mmol). The reaction produced the desired product **1f** (266 mg, 72%) as a hygroscopic pale-yellow solid. IR (KBr film) *ν*_max_ = 3425, 2931, 2224, 1439, 1077 cm^−1^. ^1^H NMR (300 MHz, MeOD) δ 7.41–7.39 (m, 3H), 7.32–7.22 (m, 2H), 6.61 (s, 1H), 5.14 (s, 1H), 4.09 (s, 1H), 3.76–3.52 (m, 4H), 1.77 (s, 4H), 1.65 (d, *J* = 13.0 Hz, 2H), 1.48 (d, *J* = 12.4 Hz, 1H), 1.20–1.03 (m, 2H), 0.93 (s, 1H). ^13^C NMR (75 MHz, MeOD) δ 144.5, 138.9, 134.2, 133.2, 131.9, 131.1, 119.8, 113.7, 94.9, 76.5, 74.5, 67.0, 66.4, 61.5, 36.1, 35.9, 28.9 (2 × CH_2_), 27.8. DEPT135 ^13^C NMR (75 MHz, MeOD) δ 133.2, 131.9, 131.1, 76.5, 74.5, 67.0, 66.4, 61.5, 36.1, 35.9, 28.9 (2xCH_2_), 27.8. HRMS (ESI-TOF) m/z: [M+H]^+^ calcd for C_21_H_27_N_2_O_4_^+^ 371.1971; found 371.1972.

1,2-Diphenyl-5-((1*R*,2*S*,3*R*)-1,2,3,4-Tetrahydroxybutyl)-1*H*-Pyrrole-3-Carbonitrile (**1h**)

Obtained by general procedure 2 using *D*-(+)-fructose **1** (180 mg, 1.0 mmol), benzoylacetonitrile **2** (145 mg, 1.0 mmol), aniline **11** (100 µL, 1.1 mmol), and AcOH (57 µL, 1.0 mmol). The reaction produced the desired product **1g** (222 mg, 61%) as a hygroscopic pale-yellow solid. IR (KBr film) *ν*_max_ = 3408, 2219, 1635, 1497, 1095 cm−^1^. ^1^H NMR (300 MHz, MeOD) δ 7.30–7.25 (br s, 4H), 7.17–7.12 (m, 3H), 7.11–7.07 (m, 2H), 7.02 (s, 1H), 6.77 (d, *J* = 0.5 Hz, 1H), 4.57 (d, *J* = 2.0 Hz, 1H), 3.56–3.46 (m, 3H), 3.41 (dt, *J* = 5.5, 2.4 Hz, 1H). ^13^C NMR (75 MHz, MeOD) δ 144.4, 140.2, 139.8, 132.4, 132.3, 131.8, 131.7, 131.5, 131.0, 130.8, 119.8, 113.6, 94.6, 76.4, 74.4, 67.3, 66.1. DEPT135 ^13^C NMR (75 MHz, MeOD) δ 132.3, 31.8, 131.5, 131.0, 130.8, 113.6, 76.4, 74.4, 67.3, 66.1. HRMS (ESI-TOF) m/z: [M+H]^+^ calcd for C_21_H_21_N_2_O_4_^+^ 365.1501; found 365.1494. 

1-(4-Methoxyphenyl)-2-Phenyl-5-((1*R*,2*S*,3*R*)-1,2,3,4-Tetrahydroxybutyl)-1*H*-Pyrrole-3-Carbonitrile (**1i**)

Obtained by general procedure 2 using *D*-(+)-fructose **1** (180 mg, 1.0 mmol), benzoylacetonitrile **2** (145 mg, 1.0 mmol), *p*-anisidine **12** (126 µL, 1.1 mmol), and AcOH (57 µL, 1.0 mmol). The reaction produced the desired product **1h** (236 mg, 60%) as a hygroscopic pale-yellow solid. IR (KBr film) *ν*_max_ = 3402, 2942, 2223, 1514, 1400, 1251, 1078 cm^−1^. ^1^H NMR (300 MHz, MeOD) δ 7.23–7.08 (m, 6H), 6.98–6.74 (m, 3H), 6.73 (s, 1H), 4.55 (d, *J* = 1.9 Hz, 1H), 3.68 (s, 3H), 3.59–3.47 (m, 3H), 3.45–3.36 (m, 1H). ^13^C NMR (75 MHz, MeOD) δ 162.8, 144.6, 140.4, 132.7, 132.6, 132.3, 131.0, 130.8, 119.9, 116.8 (CH), 116.8 (C_q_), 113.4, 94.3, 76.5, 74.5, 67.4, 66.1, 57.5. DEPT135 ^13^C NMR (75 MHz, MeOD) δ 132.7, 132.3, 131.0, 130.8, 116.8, 113.4, 76.4, 74.4, 67.3, 66.1, 57.5. HRMS (ESI-TOF) m/z: [M+Na]^+^ calcd for C_22_H_22_N_2_O_5_Na^+^ 417.1426; found 417.1478. 

2-Methyl-1-Phenethyl-5-((1*R*,2*S*,3*R*)-1,2,3,4-Tetrahydroxybutyl)-1*H*-Pyrrole-3-Carbonitrile (**1j**)

Obtained by general procedure 2 using *D*-(+)-fructose **1** (180 mg, 1.0 mmol), 3-oxobutanenitrile **13** (83 mg, 1.0 mmol), 2-phenylethylamine **6** (138 µL, 1.1 mmol), and AcOH (57 µL, 1.0 mmol). The reaction produced the desired product **1i** (234 mg, 71%) as a hygroscopic pale-yellow solid. IR (KBr film) *ν*_max_ = 3350, 2944, 2218, 1638, 1429, 1077 cm^−1^. ^1^H NMR (400 MHz, MeOD) δ 7.33–7.23 (m, 3H), 7.14 (d, *J* = 7.6 Hz, 2H), 6.51 (s, 1H), 5.00 (d, *J* = 1.9 Hz, 1H), 4.32–4.12 (m, 2H), 3.86–3.70 (m, 3H), 3.64 (dd, *J* = 11.0, 5.1 Hz, 1H), 3.03 (t, *J* = 7.3 Hz, 2H), 2.04 (s, 3H). ^13^C NMR (101 MHz, MeOD) δ 138.6, 138.1, 134.3, 128.7, 128.3, 126.5, 117.2, 108.9, 89.2, 73.4, 71.7, 64.2, 63.3, 45.9, 36.6, 9.8. DEPT135 ^13^C NMR (101 MHz, MeOD) δ 128.7, 128.3, 126.5, 108.9, 73.4, 71.7, 64.2, 63.3, 45.9, 36.6, 9.8. HRMS (ESI-TOF) m/z: [M+H]^+^ calcd for C_18_H_23_N_2_O_4_^+^ 331.1658; found 331.1620.

1-Benzyl-2-Methyl-5-((1*R*,2*S*,3*R*)-1,2,3,4-Tetrahydroxybutyl)-1*H*-Pyrrole-3-Carbonitrile (**1k**)

Obtained by general procedure 2 using *D*-(+)-fructose **1** (180 mg, 1.0 mmol), 3-oxobutanenitrile **13** (83 mg, 1.0 mmol), benzylamine **3** (120 µL, 1.1 mmol), and AcOH (57 µL, 1.0 mmol). The reaction produced the desired product **1j** (212 mg, 67%) as a hygroscopic pale-yellow solid. IR (KBr film) *ν*_max_ = 3411, 2217 1636, 1428, 1078 cm^−1^. ^1^H NMR (400 MHz, MeOD) δ 7.36–7.24 (m, 3H), 6.98 (d, *J* = 7.4 Hz, 2H), 6.56 (s, 1H), 5.41 (d, *J* = 16.0 Hz, 1H), 5.27 (d, *J* = 16.0 Hz, 1H), 4.88 (s, 1H), 3.76–3.64 (m, 3H), 3.58 (dd, *J* = 11.0, 5.5 Hz, 1H), 2.25 (s, 3H). ^13^C NMR (101 MHz, MeOD) δ 138.8, 137.1, 135.0, 128.5, 127.1, 125.5, 117.0, 108.8, 89.9, 73.1, 71.4, 64.5, 63.3, 47.4, 10.0. DEPT135 ^13^C NMR (101 MHz, MeOD) δ 128.5, 127.1, 125.5, 108.8, 73.1, 71.4, 64.5, 63.3, 47.4, 10.0. HRMS (ESI-TOF) m/z: [M+H]^+^ calcd for C_17_H_21_N_2_O_4_^+^ 317.1501; found 317.1503.

1-Hexyl-2-Phenyl-5-((1*R*,2*S*,3*R*)-1,2,3-Trihydroxy-4-(((2*S*,3*R*,4*S*,5*S*,6*R*)-3,4,5-Trihydroxy-6-(Hydroxymethyl)tetrahydro-2*H*-Pyran-2-yl)oxy)butyl)-1*H*-Pyrrole-3-Carbonitrile (**4a**)

Obtained by general procedure 2 using isomaltulose **4** (342 mg, 1.0 mmol), benzoylacetonitrile **2** (145 mg, 1.0 mmol), hexylamine **5** (144 µL, 1.1 mmol), and AcOH (57 µL, 1.0 mmol). The reaction produced the desired product **4a** (363 mg, 68%) as a hygroscopic pale-yellow solid. IR (KBr film) *ν*_max_ = 3391, 2928, 2221, 1653, 1457, 1078 cm^−1^. ^1^H NMR (300 MHz, MeOD) δ 7.45–7.37 (m, 3H), 7.33 (dd, *J* = 7.6, 1.8 Hz, 2H), 6.58 (s, 1H), 5.01 (s, 1H), 4.74 (d, *J* = 3.7 Hz, 1H), 4.04–3.76 (m, 4H), 3.71 (dd, *J* = 13.4, 3.2 Hz, 2H), 3.61–3.49 (m, 4H), 3.33 (dd, *J* = 9.6, 3.6 Hz, 1H), 3.27–3.21 (m, 1H), 1.42 (d, *J* = 5.9 Hz, 2H), 1.1–0.9 (m, 6H), 0.69 (t, *J* = 6.9 Hz, 3H). ^13^C NMR (75 MHz, MeOD) δ 144.5, 138.8, 133.1, 132.5, 131.7, 131.5, 120.0, 113.2, 101.7, 93.9, 76.7, 75.4, 75.2, 75.1, 73.2, 72.8, 71.6, 67.0, 64.1, 47.6, 33.6, 33.4, 28.6, 24.9, 15.7. DEPT135 ^13^C NMR (75 MHz, MeOD) δ 132.5, 131.7, 131.5, 113.2, 101.7, 76.7, 75.4, 75.2, 75.1, 73.1, 72.8, 71.6, 67.0, 64.1, 47.6, 33.6, 33.4, 28.6, 24.9, 15.7. HRMS (ESI-TOF) m/z: [M+Na]^+^ calcd for C_27_H_38_N_2_O_9_Na^+^ 557.2475; found 557.2474.

1-Benzyl-2-Phenyl-5-((1*R*,2*S*,3*R*)-1,2,3-Trihydroxy-4-(((2*S*,3*R*,4*S*,5*S*,6*R*)-3,4,5-Trihydroxy-6-(Hydroxymethyl)tetrahydro-2*H*-Pyran-2-yl)oxy)butyl)-1*H*-Pyrrole-3-Carbonitrile (**4b)**

Obtained by general procedure 2 using isomaltulose **4** (342 mg, 1.0 mmol), benzoylacetonitrile **2** (145 mg, 1.0 mmol), benzylamine **3** (120 µL, 1.1 mmol), and AcOH (57 µL, 1.0 mmol). The reaction produced the desired product **4b** (297 mg, 55%) as a hygroscopic pale-yellow solid. IR (KBr film) *ν*_max_ = 3402, 2938, 2221, 1636, 1400, 1077, 1028 cm^−1^. ^1^H NMR (300 MHz, MeOD) δ 7.30 (dd, *J* = 6.6, 2.7 Hz, 3H), 7.24–7.20 (m, 2H), 7.13 (dd, *J* = 10.5, 7.1 Hz, 3H), 6.76 (d, *J* = 6.7 Hz, 2H), 6.68 (s, 1H), 5.29 (d, *J* = 16.0 Hz, 1H), 5.11 (d, *J* = 17.0 Hz, 1H), 4.82 (s, 1H), 4.70 (d, *J* = 3.6 Hz, 1H), 3.94–3.86 (m, 1H), 3.72–3.68 (m, 3H), 3.60–3.56 (m, 3H), 3.46–3.38 (m, 1H), 3.32 (dd, *J* = 9.7, 3.7 Hz, 1H), 3.25 (d, *J* = 9.1 Hz, 1H). ^13^C NMR (75 MHz, MeOD) δ 145.2, 140.4, 139.3, 132.4, 132.4, 131.8, 131.4 (CH), 131.4 (C_q_), 131.3, 130.0, 128.3, 119.9, 113.5, 101.7, 94.2, 76.7, 75.2, 75.1, 73.2, 72.6, 71.4, 67.4, 64.1, 50.9. DEPT135 ^13^C NMR (75 MHz, MeOD) δ 132.4, 131.8, 131.4, 130.0, 128.3, 113.5, 101.7, 76.7, 75.2, 75.0, 73.1, 72.6, 71.4, 67.4, 64.0, 50.9. HRMS (ESI-TOF) m/z: [M+Na]^+^ calcd for C_28_H_32_N_2_O_9_Na^+^ 563.2006; found 563.2002.

1-Hexyl-2-Methyl-5-((1*R*,2*S*,3*R*)-1,2,3-Trihydroxy-4-(((2*S*,3*R*,4*S*,5*S*,6*R*)-3,4,5-Trihydroxy-6-(hydroxymethyl)tetrahydro-2*H*-Pyran-2-yl)oxy)butyl)-1*H*-Pyrrole-3-Carbonitrile (**4c**)

Obtained by general procedure 2 using isomaltulose **4** (342 mg, 1.0 mmol), 3-oxobutanenitrile **13** (83 mg, 1.0 mmol), hexylamine **5** (144 µL, 1.1 mmol), and AcOH (57 µL, 1.0 mmol). The reaction produced the desired product **4c** (269 mg, 57%) as a hygroscopic pale-yellow solid. IR (KBr film) *ν*_max_ = 3402, 2931, 2217, 1651, 1429, 1078, 1027 cm^−1^. ^1^H NMR (400 MHz, MeOD) δ 6.50 (s, 1H), 5.03 (s, 1H), 4.84 (d, *J* = 3.7 Hz, 1H), 4.11–3.99 (m, 2H), 3.98–3.89 (m, 1H), 3.87–3.76 (m, 3H), 3.71–3.60 (m, 4H), 3.44 (dd, *J* = 9.6, 3.7 Hz, 1H), 3.38–3.33 (m, 1H), 2.39 (s, 3H), 1.79–1.67 (m, 2H), 1.38 (s, 6H), 0.94 (t, *J* = 6.8 Hz, 3H). ^13^C NMR (101 MHz, MeOD) δ 137.8, 134.4, 117.3, 108.7, 98.8, 89.3, 73.8, 72.5, 72.3, 72.1, 70.3, 69.9, 68.7, 63.9, 61.2, 44.4, 31.2, 30.5, 26.2, 22.3, 13.0, 10.0. DEPT135 ^13^C NMR (101 MHz, MeOD) δ 108.7, 98.8, 73.8, 72.5, 72.3, 72.1, 70.2, 69.8, 68.7, 63.9, 61.2, 44.4, 31.2, 30.5, 26.2, 22.3, 13.0, 10.0. HRMS (ESI-TOF) m/z: [M+Na]^+^ calcd for C_22_H_36_N_2_O_9_Na^+^ 495.2319; found 495.2320.

1-Hexyl-2,5-Diphenyl-1*H*-Pyrrole-3-Carbonitrile (**14a**)

Obtained by general procedure 2 using phenacyl alcohol **14** (136 mg, 1.0 mmol), benzoylacetonitrile **2** (145 mg, 1.0 mmol), hexylamine **5** (144 µL, 1.1 mmol), and AcOH (57 µL, 1.0 mmol). The reaction produced the desired product **14a** (295 mg, 90%) as a hygroscopic pale-yellow solid. IR (KBr film) *ν*_max_ = 2956, 2929, 2857, 2220, 1604, 1474, 1346 cm^−1^. ^1^H NMR (300 MHz, CDCl_3_) δ 7.53–7.40 (m, 10H), 6.49 (s, 1H), 4.06–3.94 (m, 2H), 1.25–1.13 (m, 2H), 1.07–0.96 (m, 2H), 0.92–0.79 (m, 4H), 0.71 (t, *J* = 7.2 Hz, 3H). ^13^C NMR (75 MHz, MeOD) δ 142.4, 136.2, 132.1, 130.1, 129.7, 129.3, 129.0 (2xCH), 128.8, 128.3, 117.4, 111.8, 92.7, 45.6, 30.7, 30.1, 25.6, 22.1, 13.8. DEPT135 ^13^C NMR (75 MHz, MeOD) δ 129.7, 129.3, 129.0, 129.0, 128.8, 128.3, 111.8, 45.6, 30.7, 301., 25.6, 22.1, 13.8. HRMS (ESI-TOF) m/z: [M+Na]^+^ calcd for C_23_H_24_N_2_Na^+^ 351.1837; found 351.1845.

1-Benzyl-2,5-Diphenyl-1*H*-Pyrrole-3-Carbonitrile (**14b**)

Obtained by general procedure 2 using phenacyl alcohol **14** (136 mg, 1.0 mmol), benzoylacetonitrile **2** (145 mg, 1.0 mmol), benzylamine **3** (120 µL, 1.1 mmol), and AcOH (57 µL, 1.0 mmol). The reaction produced the desired product **14b** (294 mg, 88%) as a hygroscopic pale-yellow solid. IR (KBr film) *ν*_max_ = 2220, 1473, 1452, 1415, 1346 cm^−1^. ^1^H NMR (300 MHz, CDCl_3_) δ 7.42 (br s, 5H), 7.39–7.30 (m, 5H), 7.21–7.11 (m, 3H), 6.72–6.54 (m, 3H), 5.19 (s, 2H). ^13^C NMR (75 MHz, MeOD) δ 142.8, 137.4, 136.7, 131.6, 129.7 (CH), 129.7 (C_q_), 129.5, 129.2, 128.9, 128.6, 128.5, 128.4, 127.4, 125.9, 117.2, 112.0, 93.3, 49.1. DEPT135 ^13^C NMR (75 MHz, MeOD) δ 129.7, 129.5, 129.2, 128.9, 128.6, 128.5, 128.4, 127.4, 125.9, 112.0, 49.1. HRMS (ESI-TOF) m/z: [M+Na]^+^ calcd for C_24_H_18_N_2_Na^+^ 357.1368; found 357.1360.

2-(2-Chlorophenyl)-1-Cyclohexyl-5-Phenyl-1*H*-Pyrrole-3-Carbonitrile (**14c**)

Obtained by general procedure 2 using phenacyl alcohol **14** (136 mg, 1.0 mmol), 2-chloro benzoylacetonitrile **18** (152 mg, 1.0 mmol), cyclohexylamine **10** (127 µL, 1.0 mmol), and AcOH (57 µL, 1.0 mmol). The reaction produced the desired product **14c** (310 mg, 86%) as a hygroscopic pale-yellow solid. IR (KBr film) *ν*_max_ = 2943, 2928, 2847, 2220, 1458, 1431, 1338 cm^−1^. ^1^H NMR (400 MHz, CDCl_3_) δ 7.56 (d, *J* = 8.0 Hz, 1H), 7.49-7.40 (m, 8H), 6.44 (s, 1H), 3.91–3.82 (m, 1H), 1.95 – 1.83 (m, 2H), 1.63 (d, *J* = 14.0 Hz, 2H), 1.56-1.34 (m, 3H), 1.09 – 0.93 (m, 2H), 0.77-0.71 (m, 1H). ^13^C NMR (101 MHz, MeOD) δ 138.0, 135.9, 135.8, 133.4, 132.7, 131.1, 130.4, 129.9, 128.5, 128.3, 126.6, 116.5, 111.9, 94.4, 59.6, 33.9, 32.8, 26.2 (2xCH_2_), 25.0. DEPT135 ^13^C NMR (101 MHz, MeOD) δ 133.4, 131.1, 130.5, 129.9, 128.5, 128.3, 126.6, 111.8, 59.6, 33.9, 32.8, 26.2, 26.2, 25.0. HRMS (ESI-TOF) m/z: [M+Na]^+^ calcd for C_23_H_21_N_2_ClNa^+^ 383.1291; found 383.1295.

1,2,5-Triphenyl-1*H*-Pyrrole-3-Carbonitrile (**14d**)

Obtained by general procedure 2 using phenacyl alcohol **14** (136 mg, 1.0 mmol), benzoylacetonitrile **2** (145 mg, 1.0 mmol), aniline **11** (100 µL, 1.1 mmol), and AcOH (57 µL, 1.0 mmol). The reaction produced the desired product **14d** (256 mg, 80%) as a hygroscopic pale-yellow solid. IR (KBr film) *ν*_max_ = 2229, 1597, 1493, 1085 cm^−1^. ^1^H NMR (300 MHz, CDCl_3_) δ 7.21–7.18 (m, 5H), 7.17–7.10 (m, 6H), 7.00–6.94 (m, 2H), 6.92–6.86 (m, 2H), 6.64 (s, 1H). ^13^C NMR (75 MHz, MeOD) δ 141.9, 137.3, 136.1, 131.2, 129.7, 129.4, 129.1, 129.0, 128.6, 128.4, 128.3 (2xCH), 128.2, 127.6, 117.2, 112.0, 93.6. DEPT135 ^13^C NMR (75 MHz, MeOD) δ 129.7, 129.1, 129.0, 128.6, 128.4, 128.3 (2 × CH), 128.2, 127.6, 112.4. HRMS (ESI-TOF) m/z: [M+H]^+^ calcd for C_23_H_17_N_2_^+^ 321.1392; found 321.1379.

1-(4-Fluorophenyl)-2,5-Diphenyl-1*H*-Pyrrole-3-Carbonitrile (**14e**)

Obtained by general procedure 2 using phenacyl alcohol **14** (136 mg, 1.0 mmol), benzoylacetonitrile **2** (145 mg, 1.0 mmol), 4-fluoroaniline **21** (104 µL, 1.1 mmol), and AcOH (57 µL, 1.0 mmol). The reaction produced the desired product **14e** (260 mg, 77%) as a hygroscopic pale-yellow solid. IR (KBr film) *ν*_max_ = 2227, 1602, 1514, 1489, 1232 cm^−1^. ^1^H NMR (300 MHz, CDCl_3_) δ 7.23–7.09 (m, 8H), 6.97 (dd, *J* = 6.6, 2.9 Hz, 2H), 6.95-6.80 (m, 4H), 6.63 (d, *J* = 0.6 Hz, 1H). ^13^C NMR (75 MHz, MeOD) δ 162.0 (d, J = 247.5 Hz, C-F), 141.9, 136.2, 133.3, 131.0, 130.3, 130.2, 129.8, 129.2, 129.0, 128.6, 128.5, 128.3, 127.7, 117.0, 116.2 (d, J = 22.5 Hz, C-H), 112.1, 93.7. DEPT135 ^13^C NMR (75 MHz, MeOD) δ 130.3, 130.2, 129.8, 129.0, 128.6, 128.5, 128.3, 127.8, 116.3, 116.0, 112.1. HRMS (ESI-TOF) m/z: [M+H]^+^ calcd for C_23_H_16_N_2_F^+^ 339.1298; found 339.1283.

1-Benzyl-2-Methyl-5-(*p*-tolyl)-1*H*-Pyrrole-3-Carbonitrile (**15a**)

Obtained by general procedure 2 using 2-hydroxy-1-(*p*-tolyl)ethan-1-one **15** (150 mg, 1.0 mmol), 3-oxobutanenitrile **13** (83 mg, 1.0 mmol), benzylamine **3** (120 µL, 1.1 mmol), and AcOH (57 µL, 1.0 mmol). The reaction produced the desired product **15a** (212 mg, 74%) as a hygroscopic pale-yellow solid. IR (KBr film) *ν*_max_ = 2218, 1700, 1559, 1496, 1353, 1155 cm^−1^. ^1^H NMR (400 MHz, CDCl_3_) δ 7.35–7.28 (m, 3H), 7.15 (s, 4H), 6.90 (d, *J* = 7.1 Hz, 2H), 6.42 (s, 1H), 5.11 (s, 2H), 2.36 (s, 3H), 2.30 (s, 3H). ^13^C NMR (101 MHz, MeOD) δ 138.7, 138.1, 136.9, 135.6, 129.3, 129.1, 129.0, 128.6, 127.6, 125.5, 117.3, 109.8, 92.0, 48.2, 21.2, 11.8. DEPT135 ^13^C NMR (101 MHz, MeOD) δ 129.3, 129.1, 129.0, 127.6, 125.5, 48.2, 21.2, 11.8. HRMS (ESI-TOF) m/z: [M+H]^+^ calcd for C_20_H_19_N_2_^+^ 287.1548; found 287.1542.

2-(4-Bromophenyl)-5-(4-Fluorophenyl)-1-Phenethyl-1*H*-Pyrrole-3-Carbonitrile (**16a**)

Obtained by general procedure 2 using 1-(4-fluorophenyl)-2-hydroxyethan-1-one **16** (154 mg, 1.0 mmol), 4-bromo benzoylacetonitrile **19** (224 mg, 1.0 mmol), 2-phenylethylamine **6** (138 µL, 1.1 mmol), and AcOH (57 µL, 1.0 mmol). The reaction produced the desired product **16a** (365 mg, 82%) as a hygroscopic pale-yellow solid. IR (KBr film) *ν*_max_ = 2220, 1557, 1493, 1225, 1157, 1071 cm^−1^. ^1^H NMR (400 MHz, CDCl_3_) δ 7.65–7.55 (m, 2H), 7.34–7.28 (m, 2H), 7.22–7.11 (m, 7H), 6.67–6.51 (m, 2H), 6.46 (s, 1H), 4.21 (t, *J* = 7.0 Hz, 2H), 2.44 (t, *J* = 7.0 Hz, 2H). ^13^C NMR (101 MHz, MeOD) δ 162.8 (d, J = 242.4 Hz, C-F), 141.2, 136.7, 135.4, 132.2, 131.2, 131.1 (2xCH), 128.6, 128.4, 127.8 (2xC_q_), 126.9, 123.6, 116.8, 116.0, 115.8, 112.2, 93.2, 47.1, 36.4. DEPT135 ^13^C NMR (101 MHz, MeOD) δ 132.2, 131.2, 131.1, 131.1, 128.6, 128.4, 126.9, 115.9 (d, J = 20.2 Hz, C-H), 47.1, 36.4. HRMS (ESI-TOF) m/z: [M+H]^+^ calcd for C_25_H_19_N_2_FBr^+^ 445.0716; found 445.0730.

5-(4-Fluorophenyl)-2-Methyl-1-Phenyl-1*H*-Pyrrole-3-Carbonitrile (**16b**)

Obtained by general procedure 2 using 1-(4-fluorophenyl)-2-hydroxyethan-1-one **16** (154 mg, 1.0 mmol), 3-oxobutanenitrile **13** (83 mg, 1.0 mmol), aniline **11** (100 µL, 1.1 mmol), and AcOH (57 µL, 1.0 mmol). The reaction produced the desired product **16b** (193 mg, 70%) as a hygroscopic pale-yellow solid. IR (KBr film) *ν*_max_ = 2221, 1700, 1559, 1496, 1225, 1160 cm^−1^. ^1^H NMR (400 MHz, CDCl_3_) δ 7.47–7.42 (m, 3H), 7.16–7.11 (m, 2H), 7.04–6.98 (m, 2H), 6.88 (t, *J* = 8.7 Hz, 2H), 6.52 (s, 1H), 2.29 (s, 3H). ^13^C NMR (101 MHz, MeOD) δ 162.0 (d, J = 252.5 Hz, C-F), 139.9, 137.4, 134.2, 130.1, 130.0, 129.5, 128.8, 128.1, 127.6, 117.0, 115.3 (d, J = 20.2 Hz, C-H), 110.1, 92.5, 12.4. DEPT135 ^13^C NMR (101 MHz, MeOD) δ 130.1, 130.0, 129.5, 128.8, 128.1, 115.4, 115.2, 110.0, 12.4. HRMS (ESI-TOF) m/z: [M+H]^+^ calcd for C_18_H_14_N_2_F^+^ 277.1141; found 277.1138.

1.-Benzyl-5-(4-Chlorophenyl)-2-(3-Methoxyphenyl)-1*H*-Pyrrole-3-Carbonitrile (**17a**)

Obtained by general procedure 2 using 1-(4-chlorophenyl)-2-hydroxyethan-1-one **17** (170 mg, 1.0 mmol), 3-methoxy benzoylacetonitrile **20** (175 mg, 1.0 mmol), benzylamine **3** (120 µL, 1.1 mmol), and AcOH (57 µL, 1.0 mmol). The reaction produced the desired product **17a** (334 mg, 84%) as a hygroscopic pale-yellow solid. IR (KBr film) *ν*_max_ = 2221, 1603, 1557, 1487, 1287, 1093 cm^−1^. ^1^H NMR (400 MHz, CDCl_3_) δ 7.38–7.30 (m, 3H), 7.24–7.20 (m, 5H), 7.03 (d, *J* = 7.9 Hz, 1H), 6.96 (dd, *J* = 8.3, 2.5 Hz, 1H), 6.91 (d, *J* = 1.4 Hz, 1H), 6.76–6.66 (m, 2H), 6.60 (s, 1H), 5.17 (s, 2H), 3.69 (s, 3H). ^13^C NMR (101 MHz, MeOD) δ 159.7, 142.9, 137.5, 135.3, 134.5, 130.6, 130.5, 130.0, 128.8, 128.7, 127.6, 125.8, 122.1, 116.9, 115.6, 114.6, 112.4, 93.5, 55.2, 49.2. DEPT135 ^13^C NMR (101 MHz, MeOD) δ 130.6, 130.0, 128.9, 128.7, 127.6, 125.8, 122.1, 115.6, 114.6, 112.4, 55.2, 49.2. HRMS (ESI-TOF) m/z: [M+H]^+^ calcd for C_25_H_20_N_2_OCl^+^ 399.1264; found 399.1274.

5-(4-Chlorophenyl)-2-Methyl-1-Phenyl-1*H*-Pyrrole-3-Carbonitrile (**17b**)

Obtained by general procedure 2 using 1-(4-chlorophenyl)-2-hydroxyethan-1-one **17** (170 mg, 1.0 mmol), 3-oxobutanenitrile **13** (83 mg, 1.0 mmol), aniline **11** (100 µL, 1.1 mmol), and AcOH (57 µL, 1.0 mmol). The reaction produced the desired product **17b** (205 mg, 70%) as a hygroscopic pale-yellow solid. IR (KBr film) *ν*_max_ = 2223, 1700, 1559, 1496, 1399, 1094 cm^−1^. ^1^H NMR (400 MHz, CDCl_3_) δ 7.48–7.42 (m, 3H), 7.19–7.11 (m, 4H), 6.96 (d, *J* = 8.5 Hz, 2H), 6.56 (s, 1H), 2.29 (s, 3H). ^13^C NMR (101 MHz, MeOD) δ 140.2, 137.3, 133.9, 133.2, 129.9, 129.6, 129.4, 128.9, 128.5, 128.1, 116.9, 110.4, 92.7, 12.4. DEPT135 ^13^C NMR (101 MHz, MeOD) δ 129.6, 129.4, 128.9, 128.5, 128.1, 110.4, 12.4. HRMS (ESI-TOF) m/z: [M+H]^+^ calcd for C_18_H_14_N_2_Cl^+^ 293.0846; found 293.0843.

5-Formyl-1-Phenethyl-2-Phenyl-1*H*-Pyrrole-3-Carbonitrile (**22**)

NaIO_4_ (235 mg, 1.1 mmol) was slowly added to a solution of **1c** (392 mg, 1.0 mmol) in MeOH (10 mL) at room temperature, and the reaction mixture was stirred at room temperature for 3 h. The reaction mixture was then concentrated under vacuum, diluted with H_2_O (5 mL), and extracted with ethyl acetate (3 × 30 mL). The combined organic extracts were dried over magnesium sulfate and filtered, and the solvent was then evaporated under vacuum to produce **22** (295 mg, 98%) as a pale-yellow solid. IR (KBr film) *ν*_max_ = 2228, 1674, 1457, 1404, 1363, 1142 cm^−1^. ^1^H NMR (400 MHz, CDCl_3_) δ 9.67 (s, 1H), 7.50-7.46 (m, 3H), 7.33 (s, 1H), 7.22–7.15 (m, 5H), 6.87 (d, *J* = 6.5 Hz, 2H), 4.55 (t, *J* = 7.2 Hz, 2H), 2.90 (t, *J* = 7.1 Hz, 2H). ^13^C NMR (101 MHz, MeOD) δ 179.4, 148.5, 137.0, 131.7, 130.2, 129.5, 129.0, 128.7, 128.7, 127.3, 127.3, 126.9, 115.2, 95.0, 48.3, 37.3. DEPT135 ^13^C NMR (101 MHz, MeOD) δ 179.4, 130.2, 129.5, 129.0, 128.7, 128.7, 127.3, 126.9, 48.3, 37.3. HRMS (ESI-TOF) m/z: [M+H]^+^ calcd for C_20_H_17_N_2_O^+^ 301.1341; found 301.1335.

5-(Hydroxymethyl)-1-Phenethyl-2-Phenyl-1*H*-Pyrrole-3-Carbonitrile (**23**)

NaBH_4_ (28 mg, 0.74 mmol) was added to a stirred solution of **22** (200 mg, 0.67 mmol) in MeOH (10 mL) at room temperature. Stirring was continued for 1 h (TLC), and the solvent was then evaporated under reduced vacuum. The residue was flushed via silica gel column chromatography and was eluted with a gradient of 40% EtOAc/ hexane to produce **23** (191 mg, 95% yield) as a pale-yellow solid. IR (KBr film) *ν*_max_ = 3478, 2221, 1674, 1481, 1346 cm^−1^. ^1^H NMR (400 MHz, CDCl_3_) δ 7.51-7.37 (m, 3H), 7.41–7.35 (m, 2H), 7.22-7.19 (m, 3H), 6.84 (s, 2H), 6.45 (s, 1H), 4.51 (s, 2H), 4.27 (t, *J* = 7.4 Hz, 2H), 2.78 (t, *J* = 7.3 Hz, 2H). ^13^C NMR (101 MHz, MeOD) δ 142.7, 137.4, 133.3, 129.6, 129.6, 129.3, 129.0, 128.7, 128.6, 126.9, 117.0, 111.9, 92.1, 56.6, 46.6, 37.2. DEPT135 ^13^C NMR (101 MHz, MeOD) δ 129.6, 129.3, 129.0, 128.7, 128.6, 126.9, 111.9, 56.6, 46.6, 37.2. HRMS (ESI-TOF) m/z: [M+H]^+^ calcd for C_20_H_19_N_2_O^+^ 303.1497; found 303.1494.

## 4. Conclusions

In summary, we established an efficient and simple three-component reaction between α-hydroxyketones, oxoacetonitriles, and primary amines to selectively give densely functionalized 3-cyanopyrroles in 55–90% isolated yields. The structure and the substitution pattern of the products were unambiguously confirmed by single-crystal X-ray analysis. The practicality of this method was demonstrated on large-scale syntheses of several pyrroles. Noteworthy is the high atom economy of this reaction which makes it attractive for practical applications. Currently, we are expanding the scope of this reaction and using the process to synthesize different pyrrole-based drug candidates and intermediates such as the anti-tuberculosis drugs BM212, BM521, and BM533.

## Figures and Tables

**Table 1:**
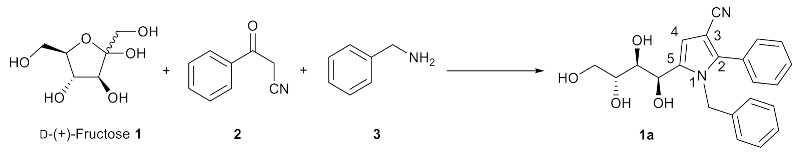
Optimization of the reaction conditions for the three-component synthesis of 3-cyanopyrrole **1a**.

Entry	Catalyst (eq.)	Solvent	Temp.	Yield (%) ^[a]^
**1**	**AcOH (1.0)**	**EtOH**	**70** **°** **C**	**77**
2	ZnCl_2_ (1.0)	EtOH	70 °C	46
3	PTSA (1.0)	EtOH	70 °C	ND ^[b]^
4	CSA (1.0)	EtOH	70 °C	ND
5	Et_3_N (1.0)	EtOH	70 °C	Trace
6	NaOH (1.0)	EtOH	70 °C	ND
7	K_2_CO_3_ (1.0)	EtOH	70 °C	ND
8	NaOMe (1.0)	EtOH	70 °C	ND
9	AcOH (1.0)	ACN	70 °C	69
10	AcOH (1.0)	DMF	70 °C	76
11	AcOH (0.5)	EtOH	70 °C	58
12	AcOH (1.0)	EtOH	40 °C	Trace

^[a]^ Isolated yields. All reactions were conducted at 70 °C for 3 h using 3 mL of the solvent. ^[b]^ ND: Not detected.

**Table 2:**
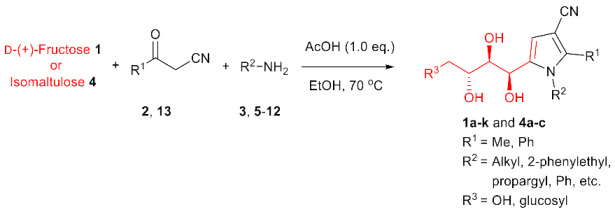
Substrate scope of the AcOH-catalyzed three-component reaction between ketoses **1**, and **4**, primary amines **3** and **5**–**12**, and oxoacetonitriles **2** and **13** for the selective synthesis of 3-cyanopyrroles **1a**–**k** and **4a**–**c** ^[a]^.

No.	Ketose	Oxoacetonitrile	Amine	3-Cyanopyrrole	Yield ^[b]^
1	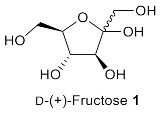	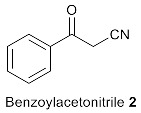	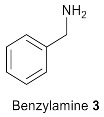	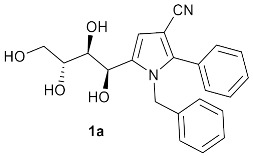	77
2	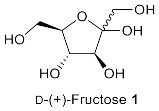	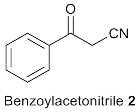	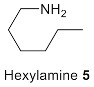	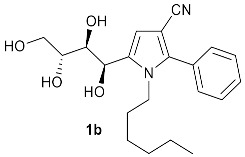	86
3	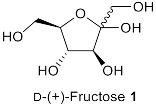	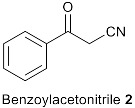	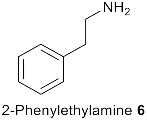	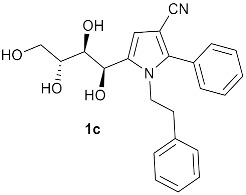	84
4	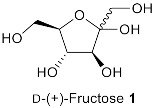	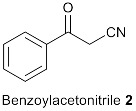	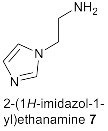	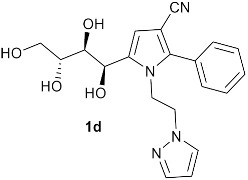	73
5	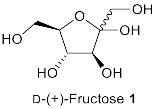	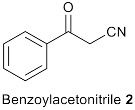	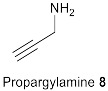	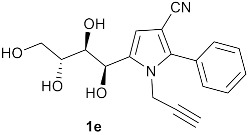	75
6	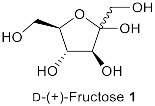	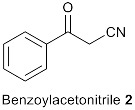	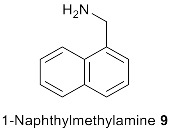	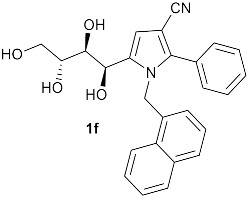	68
7	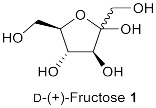	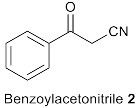	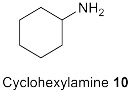	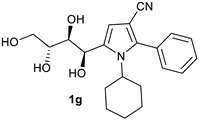	72
8	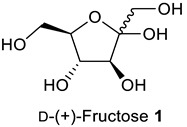	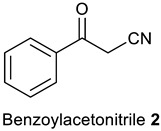	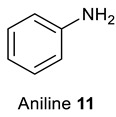	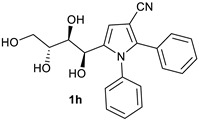	61
9	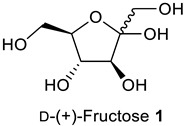	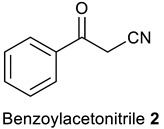	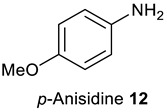	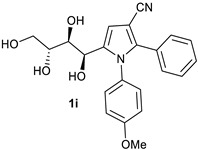	60
10	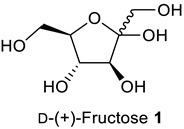	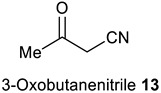	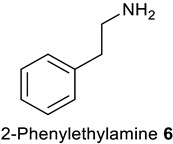	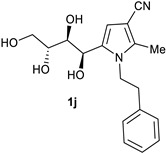	71
11	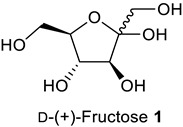	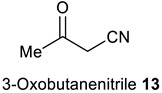	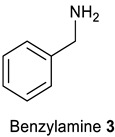	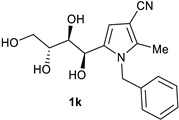	67
12	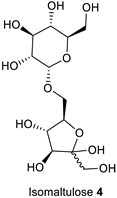	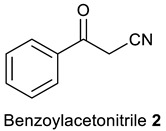	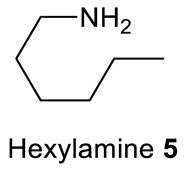	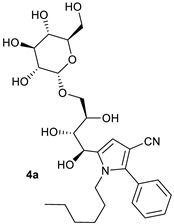	68
13	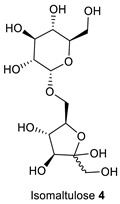	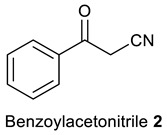	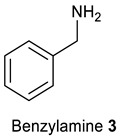	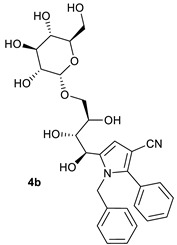	55
14	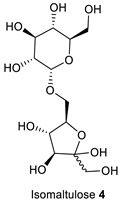	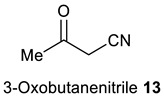	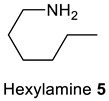	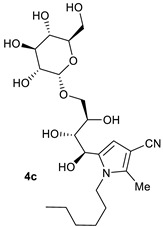	57

^[a]^ A mixture of ketoses **1** or **4** (1.0 mmol), oxoacetonitriles **2** or **13** (1.0 mmol), primary amines **3** or **5**–**12** (1.1 mmol) and AcOH (1.0 mmol) were stirred in EtOH (3 mL) at 70 °C for 3 h. ^[b]^ Isolated yields.

**Table 3:**
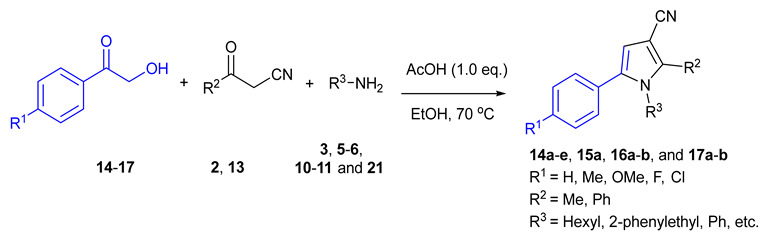
The substrate scope of the AcOH-catalyzed three-component reaction between phenacyl alcohols **14**–**17**, oxoacetonitriles **2**, **13**, and **18**–**20**, and primary amines **3**, **5**, **6**, **10**, **11**, and **21** for the selective synthesis of 3-cyanopyrroles **14a**–**e**, **15a**, **16a**–**b**, and **17a**–**b** ^[a]^.

No.	α-Hydroxyketon	Oxoacetonitrile	Amine	3-Cyanopyrrole	Yield ^[b]^
1	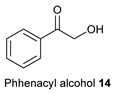	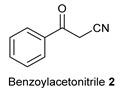	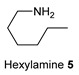	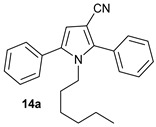	90
2	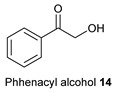	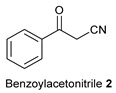	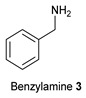	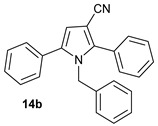	88
3	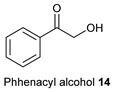	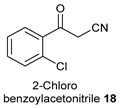	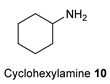	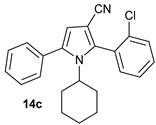	86
4	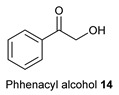	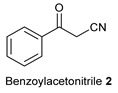	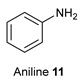	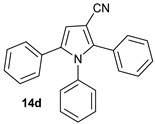	80
5	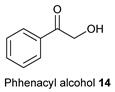	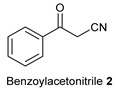	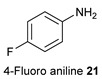	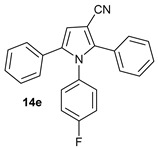	77
6	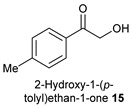	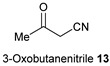	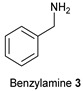	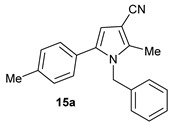	74
7	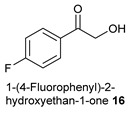	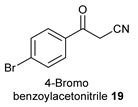	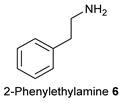	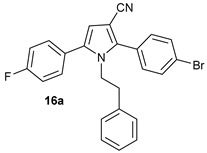	82
8	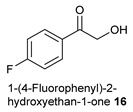	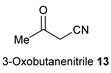	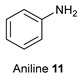	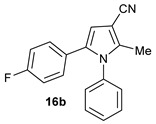	70
9	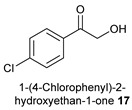	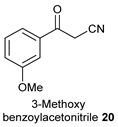	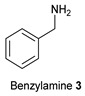	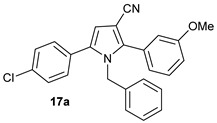	84
10	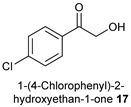	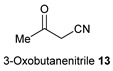	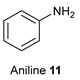	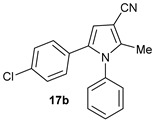	70

^[a]^ A mixture of phenacyl alcohols **14**–**17** (1.0 mmol), oxoacetonitriles **2**, **13** and **18**–**20** (1.0 mmol), and primary amines **3**, **5**, **6**, **10**, **11** and **21** (1.1 mmol) were stirred in EtOH (3 mL) at 70 °C for 3 h. ^[b]^ Isolated yields.

## Data Availability

The data presented in this study are available in supplementary materials.

## Supplementary Materials

The following supporting information can be downloaded at: https://www.mdpi.com/article/10.3390/molecules27165285/s1. Copies of the ^1^H NMR, ^13^C NMR, and 2D NMR spectra of the synthesized compounds as well as single-crystal X-ray data of pyrrole **1c** and **13c** are available online.

## Sample Availability

Samples of the compounds are available from the authors.
