# Supplementary material for: Selective One-Pot Multicomponent Synthesis of N-Substituted 2,3,5-Functionalized 3-Cyanopyrroles via the Reaction between α-Hydroxyketones, Oxoacetonitriles, and Primary Amines
Source: Molecules. 2022 Aug 18;27(16):5285. doi: 10.3390/molecules27165285 (PMC9416797; doi:10.3390/molecules27165285)
Supplement: Supplementary file 1 [file molecules-27-05285-s001.zip › molecules-1857406-supplementary.pdf]

Supporting Information

Article

Selective One-Pot Multicomponent Synthesis of *N*-Substituted 2,3,5-Functionalized 3-Cyanopyrroles via the Reaction between α-Hydroxyketones, Oxoacetonitriles, and Primary Amines

Mengxin Xia [1], Ziad Moussa [2] and Zaher M. A. Judeh [1,*]

1. School of Chemical and Biomedical Engineering, Nanyang Technological University, 62 Nanyang Drive, N1.2–B1-14, Singapore 637459, Singapore
2. Department of Chemistry, College of Science, United Arab Emirates University, Al Ain P.O. Box 15551, United Arab Emirates

* Correspondence: zaher@ntu.edu.sg; Tel.: +65-67906738; Fax: +65-67947553

	Table of Contents	Figures
1.	^{1}H NMR and ^{13}C NMR spectra of pyrroles **1a–k**, **4a–c**, **14a–e, 15a**, **16a–b**, **17a–b**, **22** and **23**	S1–S26
2.	Single-crystal X-ray and crystal parameters of pyrroles **1c** and **14c**	S27, S28
3.	2D NMR spectra of **14b**	S29

1. ^{1}H NMR and ^{13}C NMR spectra

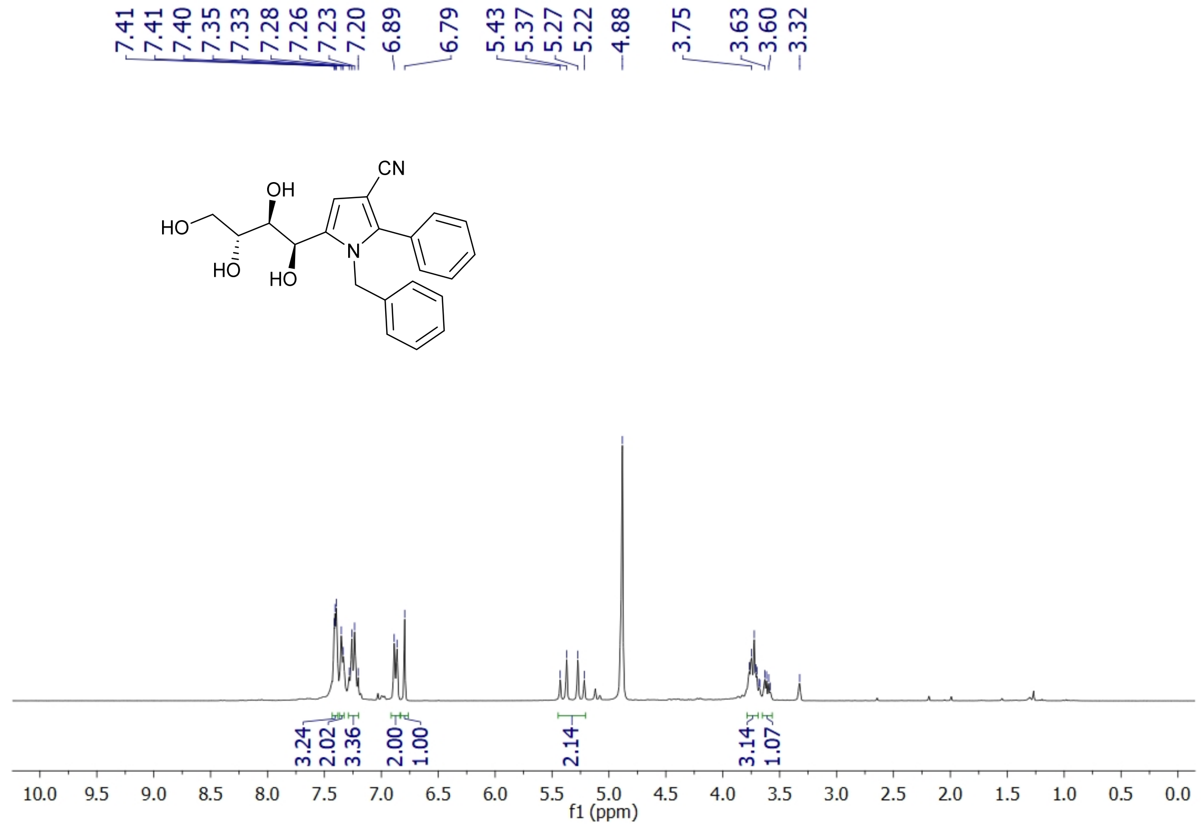

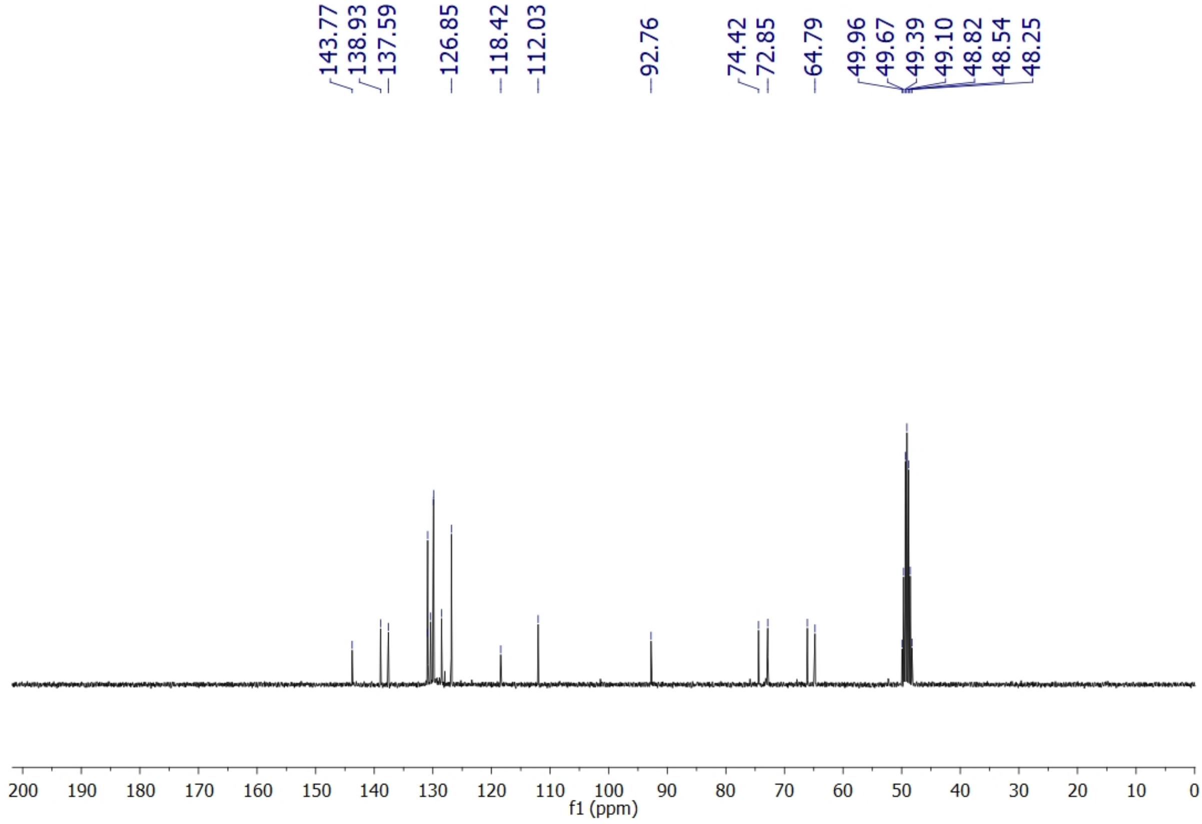

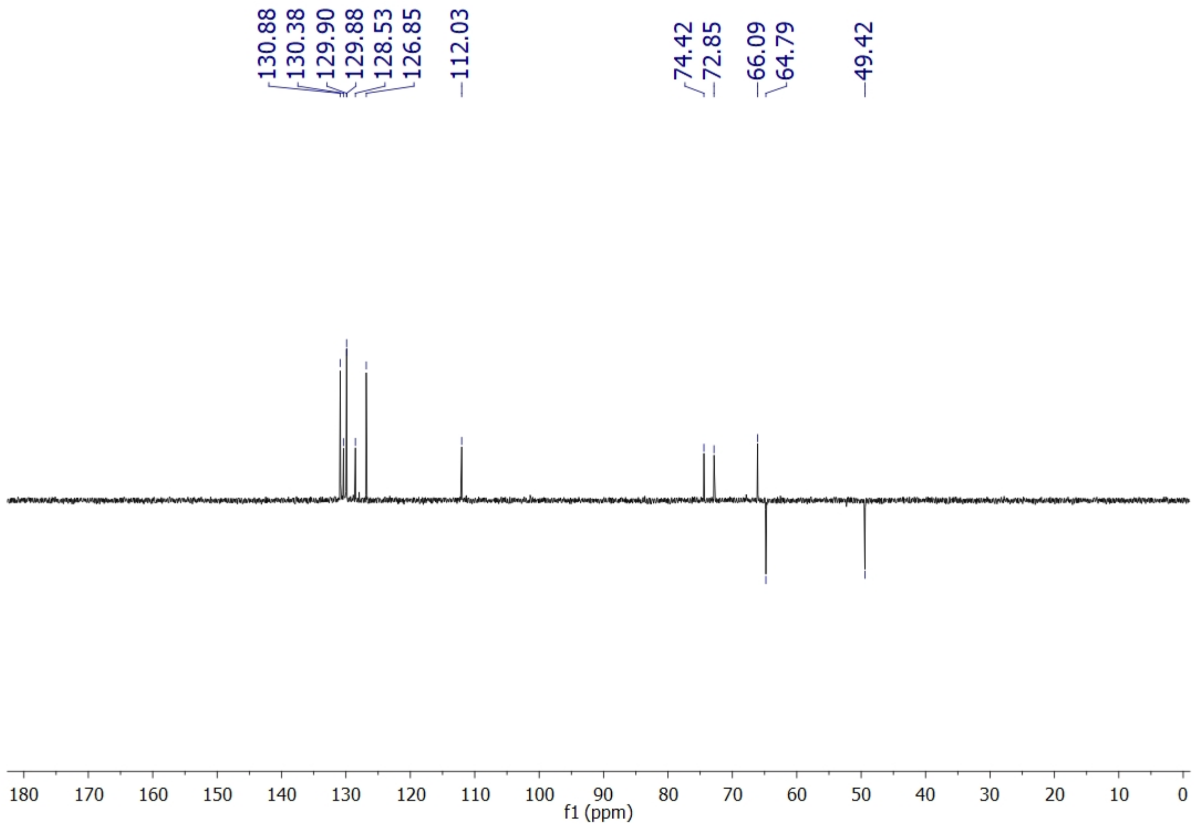

Figure S1. ^{1}H NMR and ^{13}C NMR spectra of 1-benzyl-2-phenyl-5-((1*R*,2*S*,3*R*)-1,2,3,4-tetrahydroxybutyl)-1*H*-pyrrole-3-carbonitrile.

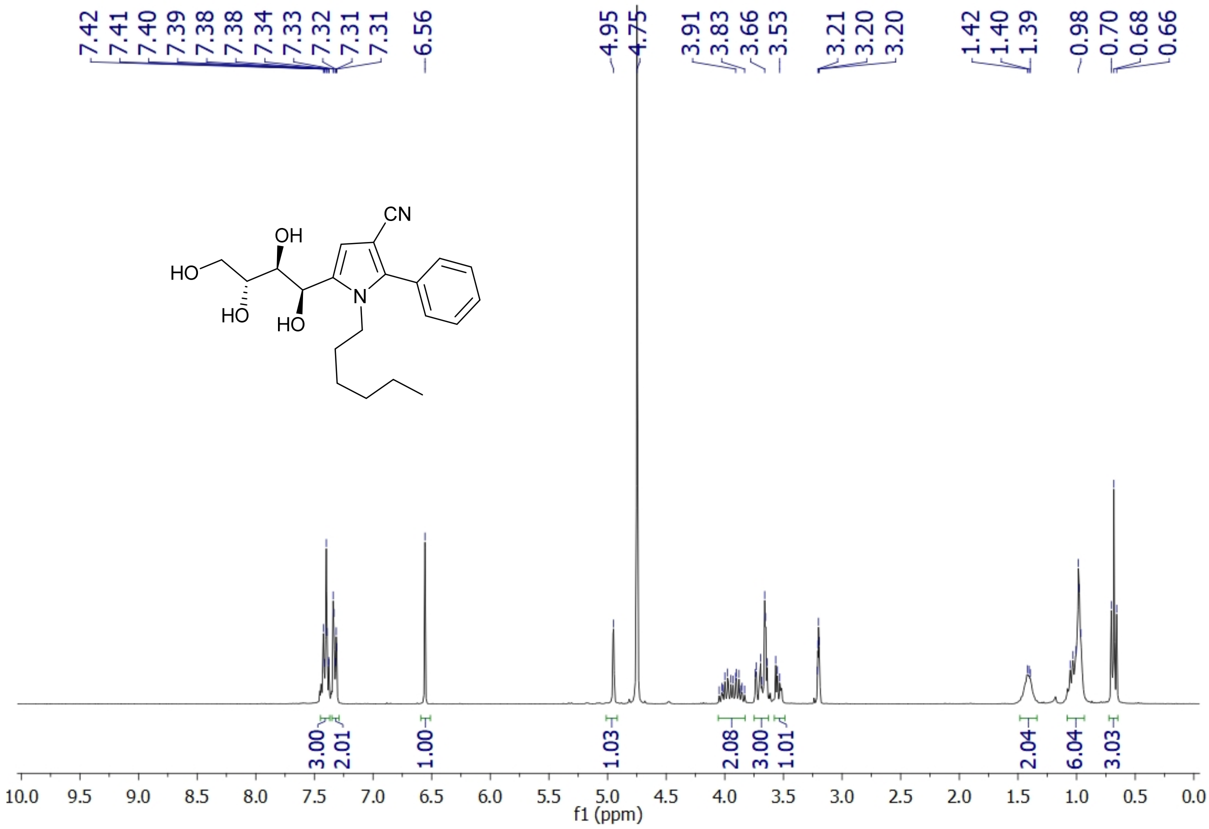
7.42
7.41
7.40
7.39
7.38
7.38
7.34
7.33
7.32
7.31
7.31
6.56
4.95
4.75
3.91
3.83
3.66
3.53
3.21
3.20
3.20
1.42
1.40
1.39
0.98
0.70
0.68
0.66
CN
OH
HO
HO
HO
N
3.00
2.01
1.00
1.03
2.08
3.00
1.01
2.04
6.04
3.03
10.0 9.5 9.0 8.5 8.0 7.5 7.0 6.5 6.0 5.5 5.0 4.5 4.0 3.5 3.0 2.5 2.0 1.5 1.0 0.5 0.0
f1 (ppm)

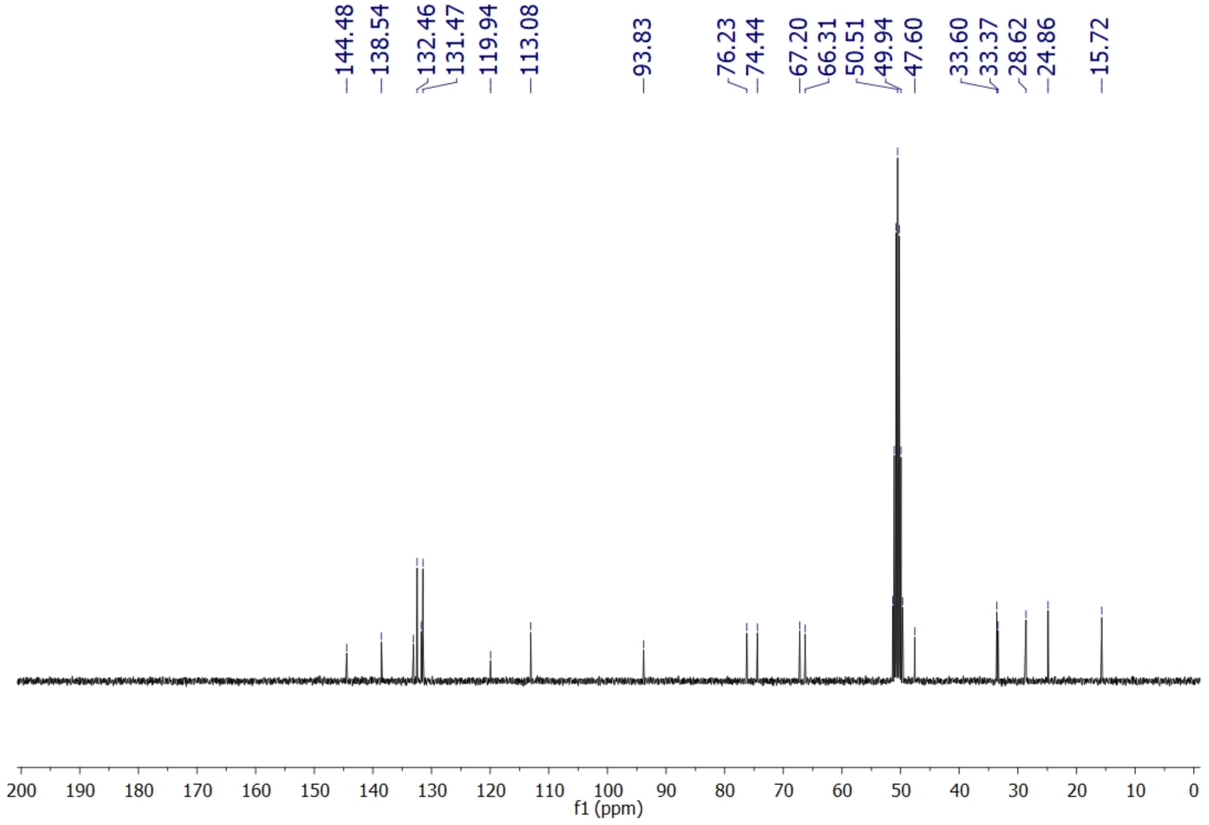
144.48
138.54
132.46
131.47
119.94
113.08
93.83
76.23
74.44
67.20
66.31
50.51
49.94
47.60
33.60
33.37
28.62
24.86
15.72
200 190 180 170 160 150 140 130 120 110 100 90 80 70 60 50 40 30 20 10 0
f1 (ppm)

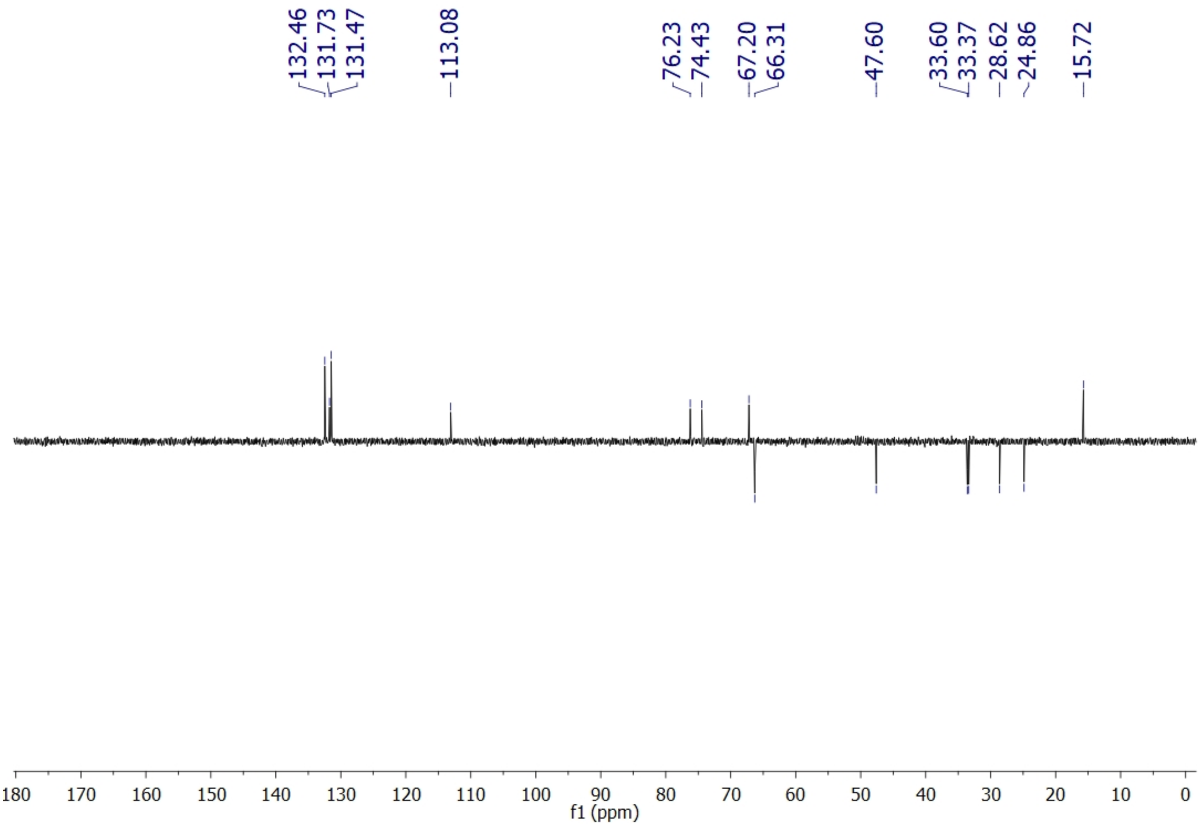

Figure S2. ^{1}H NMR and ^{13}C NMR spectra of 1-Hexyl-2-phenyl-5-((1*R*,2*S*,3*R*)-1,2,3,4-tetrahydroxybutyl)-1*H*-pyrrole-3-carbonitrile.

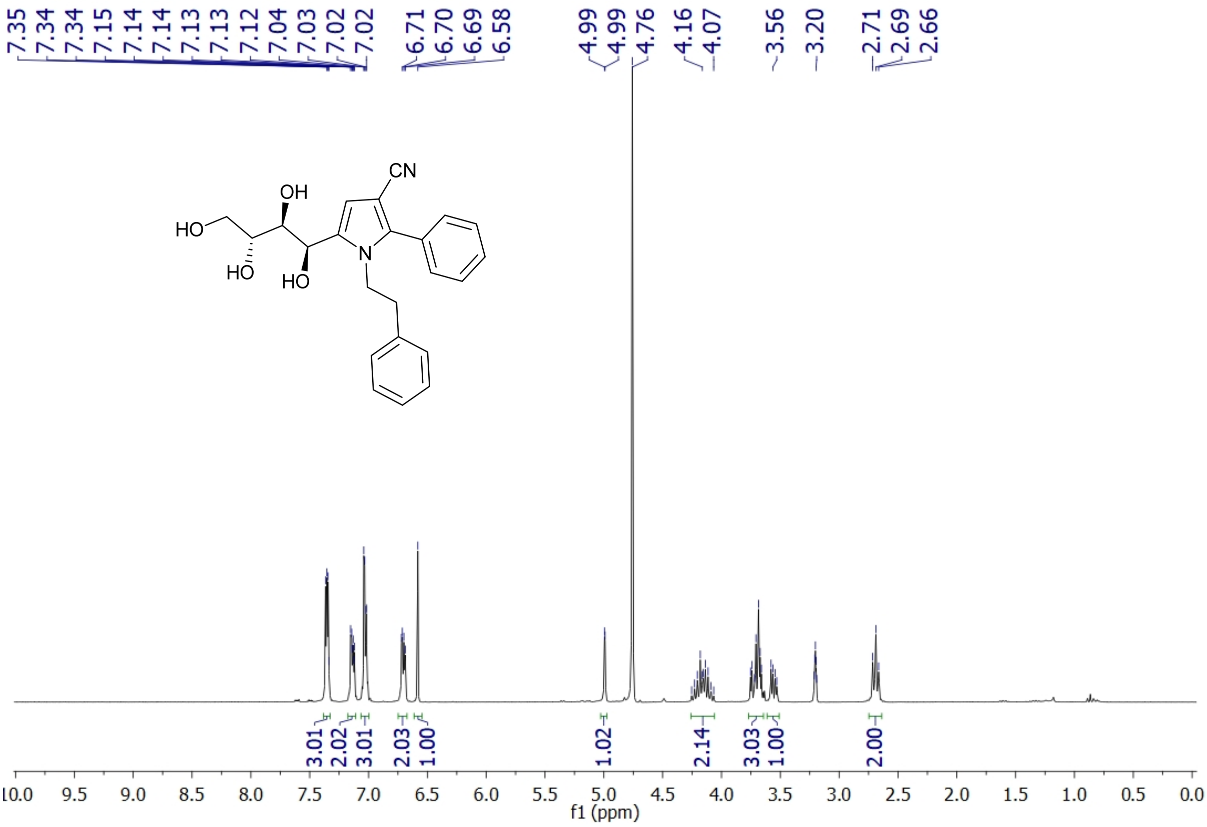
7.35
7.34
7.34
7.15
7.14
7.14
7.13
7.13
7.12
7.04
7.03
7.02
7.02
6.71
6.70
6.69
6.58
4.99
4.99
4.76
4.16
4.07
3.56
3.20
2.71
2.69
2.66
CN
OH
HO
HO
HO
N
3.01
2.02
3.01
2.03
1.00
1.02
2.14
3.03
1.00
2.00
10.0 9.5 9.0 8.5 8.0 7.5 7.0 6.5 6.0 5.5 5.0 4.5 4.0 3.5 3.0 2.5 2.0 1.5 1.0 0.5 0.0
f1 (ppm)

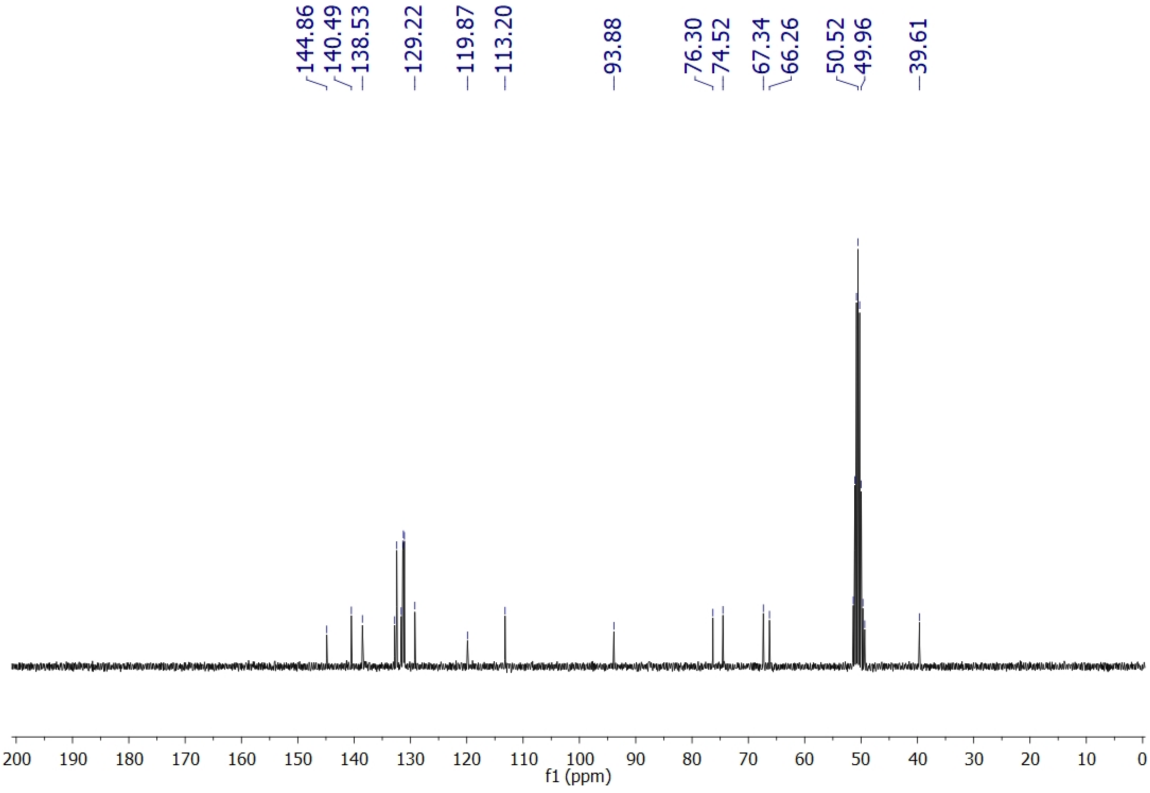
144.86
140.49
138.53
129.22
119.87
113.20
93.88
76.30
74.52
67.34
66.26
50.52
49.96
39.61
200 190 180 170 160 150 140 130 120 110 100 90 80 70 60 50 40 30 20 10 0
f1 (ppm)

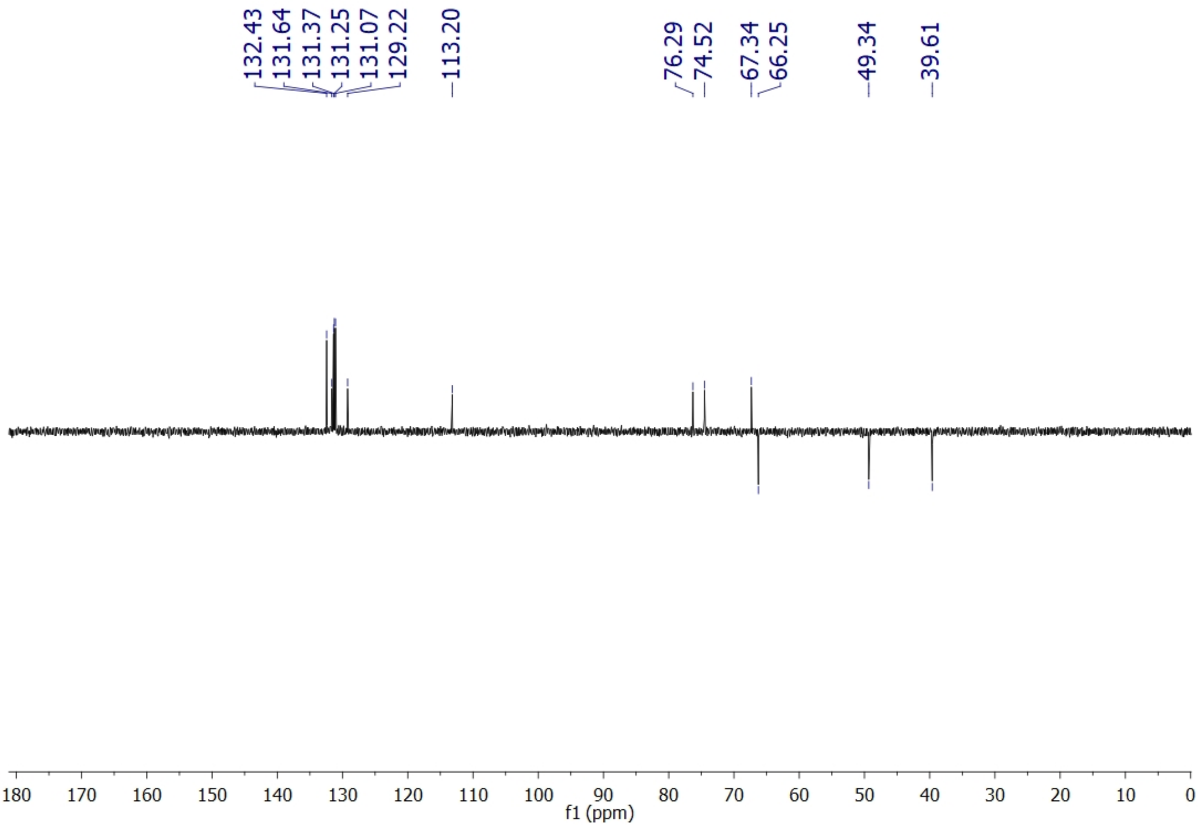

Figure S3. ^{1}H NMR and ^{13}C NMR spectra of 1-Phenethyl-2-phenyl-5-((1*R*,2*S*,3*R*)-1,2,3,4-tetrahydroxybutyl)-1*H*-pyrrole-3-carbonitrile.

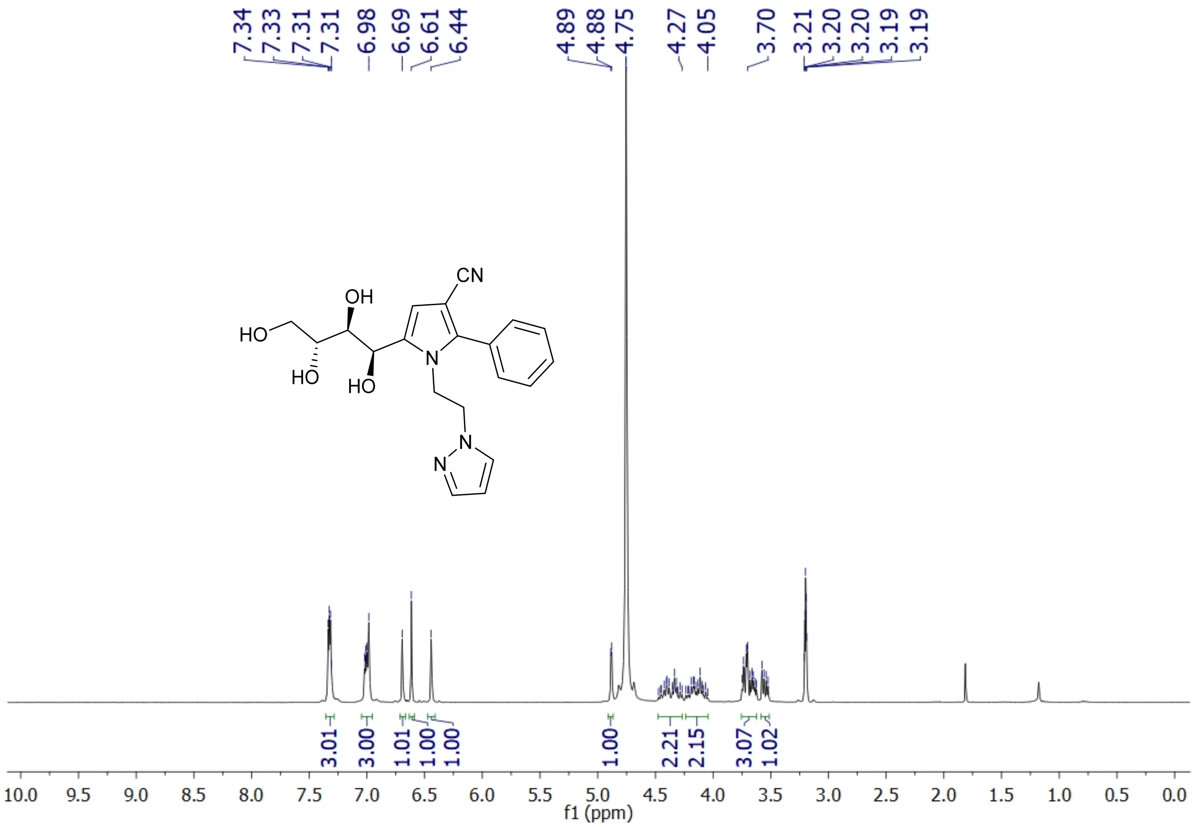
7.34
7.33
7.31
7.31
6.98
6.69
6.61
6.44
4.89
4.88
4.75
4.27
4.05
3.70
3.21
3.20
3.20
3.19
3.19
CN
OH
HO
HO
HO
N
N
N
3.01
3.00
1.01
1.00
1.00
1.00
2.21
2.15
3.07
1.02
10.0 9.5 9.0 8.5 8.0 7.5 7.0 6.5 6.0 5.5 5.0 4.5 4.0 3.5 3.0 2.5 2.0 1.5 1.0 0.5 0.0
f1 (ppm)

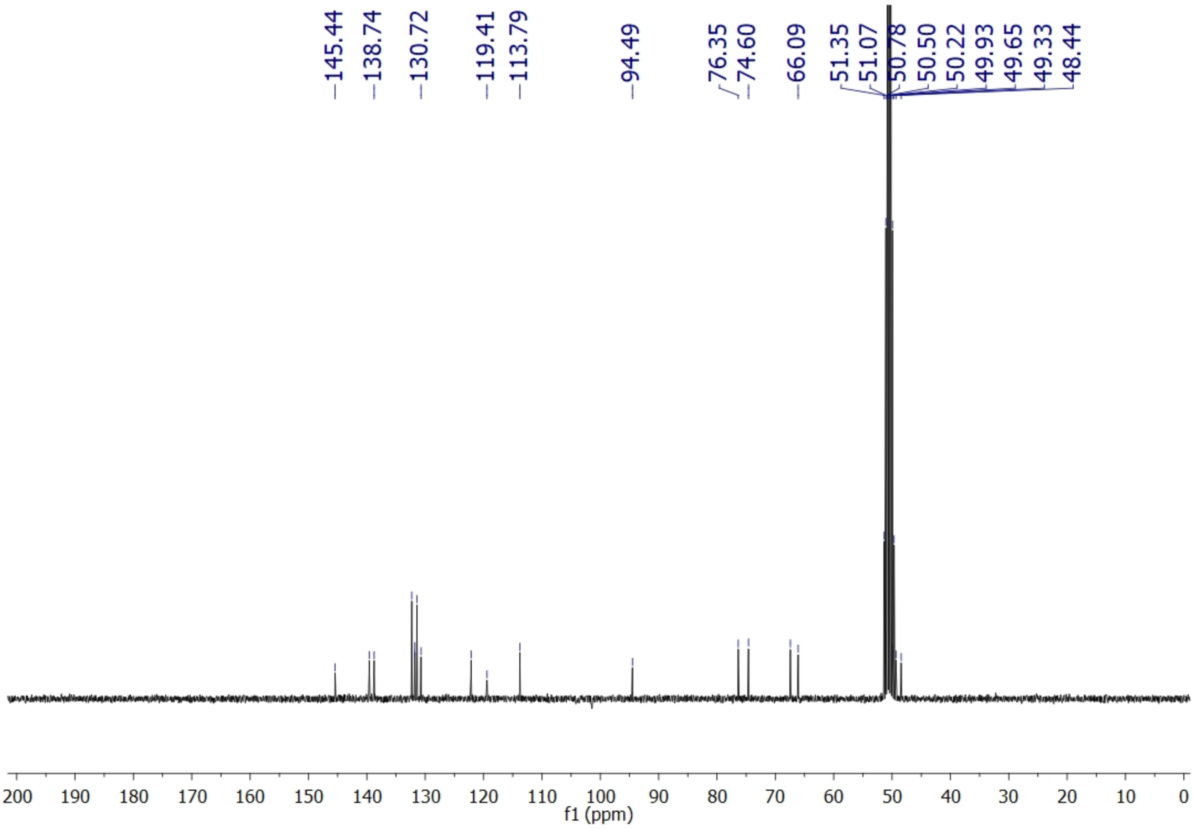
145.44
138.74
130.72
119.41
113.79
94.49
76.35
74.60
66.09
51.35
51.07
50.78
50.50
50.22
49.93
49.65
49.33
48.44
200 190 180 170 160 150 140 130 120 110 100 90 80 70 60 50 40 30 20 10 0
f1 (ppm)

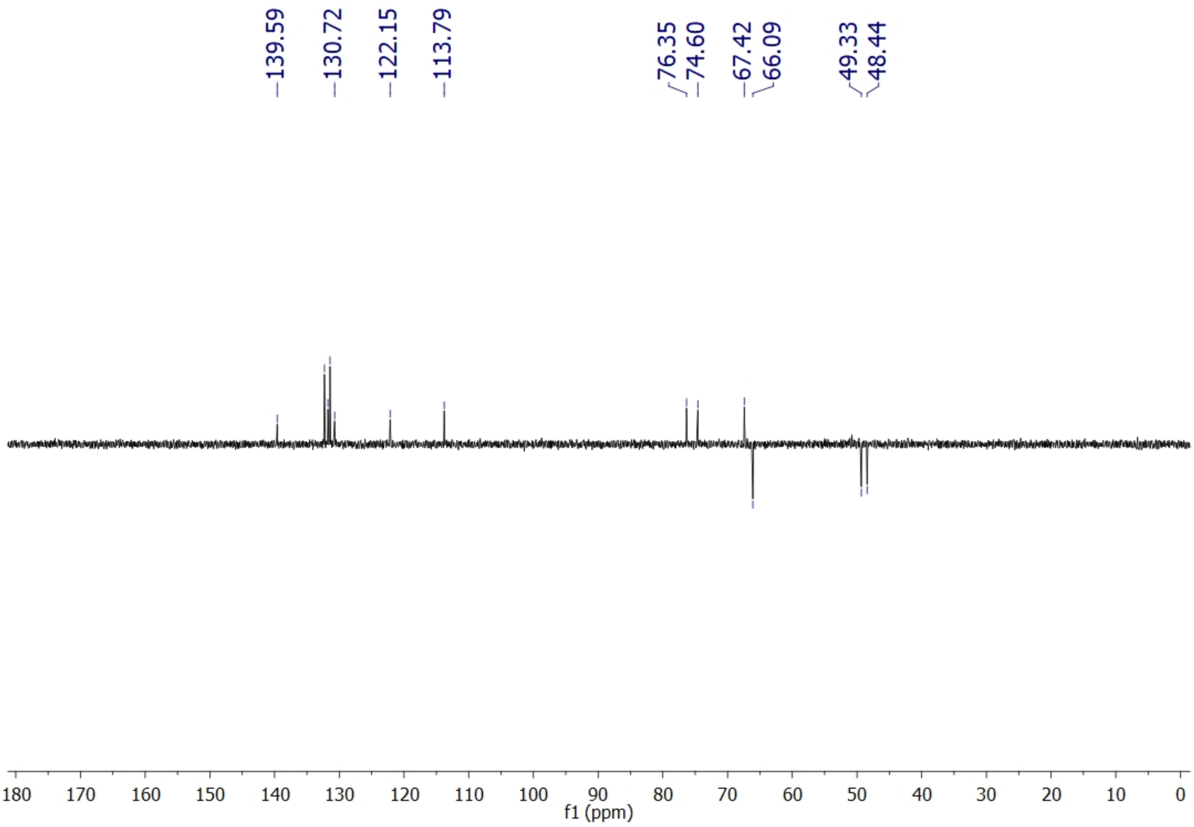

Figure S4. ^{1}H NMR and ^{13}C NMR spectra of 1-(2-(1*H*-Imidazol-1-yl)ethyl)-2-phenyl-5-((1*R*,2*S*,3*R*)-1,2,3,4-tetrahydroxybutyl)-1*H*-pyrrole-3-carbonitrile.

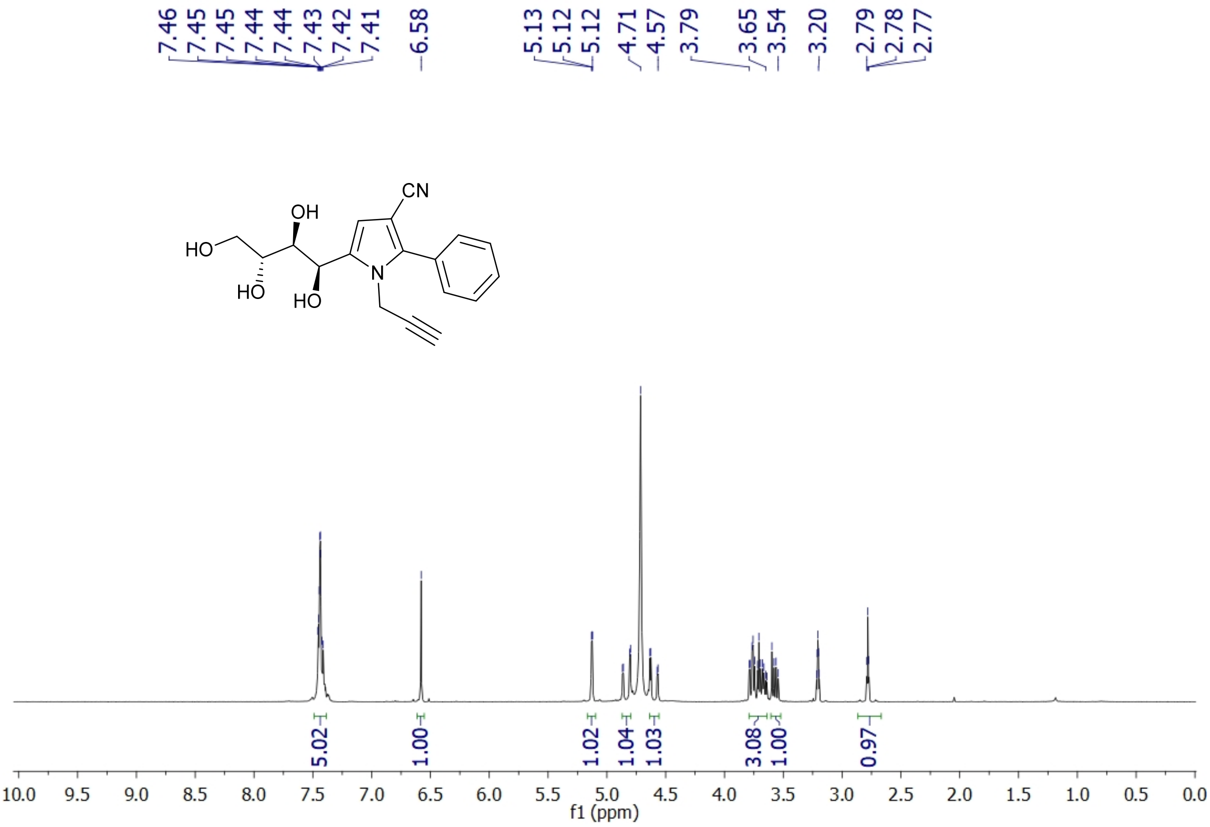
7.46
7.45
7.45
7.44
7.44
7.43
7.42
7.41
6.58
5.13
5.12
5.12
4.71
4.57
3.79
3.65
3.54
3.20
2.79
2.78
2.77
CN
OH
HO
HO
HO
N
5.02
1.00
1.02
1.04
1.03
3.08
1.00
0.97
10.0 9.5 9.0 8.5 8.0 7.5 7.0 6.5 6.0 5.5 5.0 4.5 4.0 3.5 3.0 2.5 2.0 1.5 1.0 0.5 0.0
f1 (ppm)

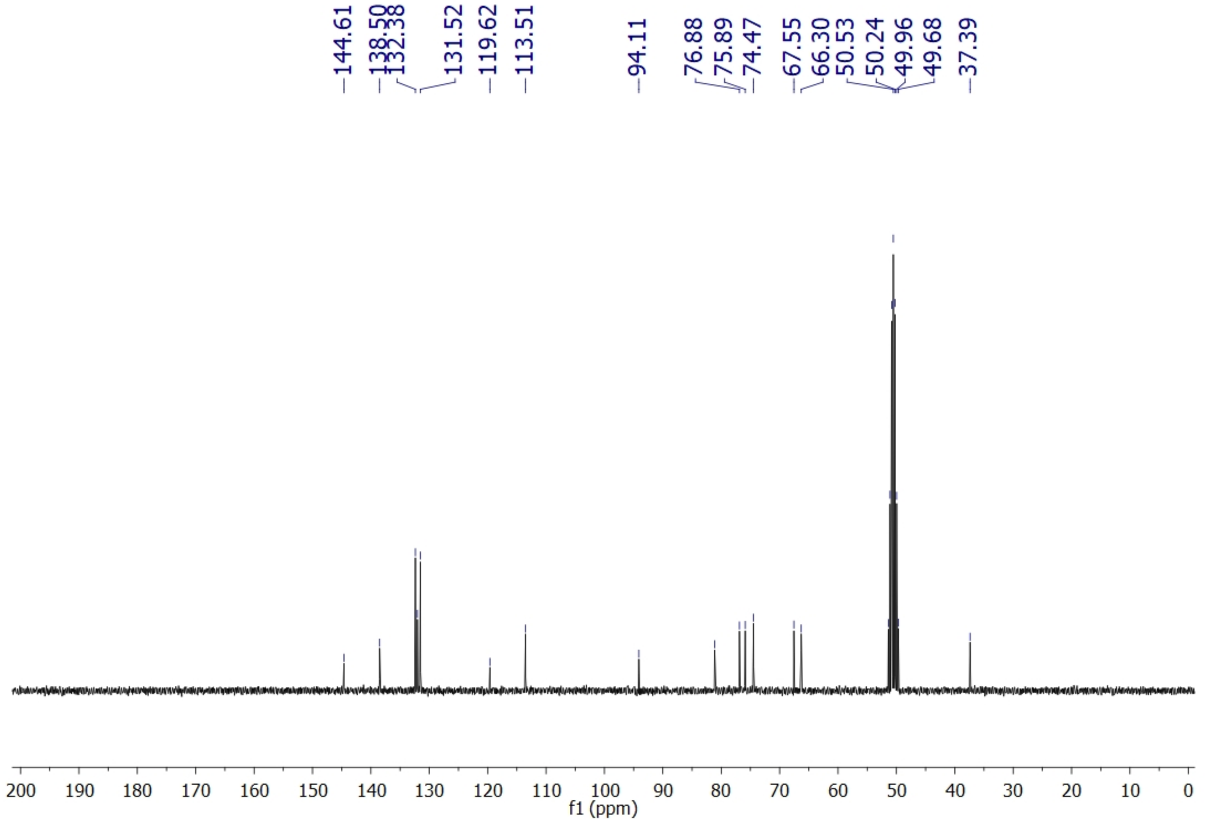
144.61
138.50
132.38
131.52
119.62
113.51
94.11
76.88
75.89
74.47
67.55
66.30
50.53
50.24
49.96
49.68
37.39
200 190 180 170 160 150 140 130 120 110 100 90 80 70 60 50 40 30 20 10 0
f1 (ppm)

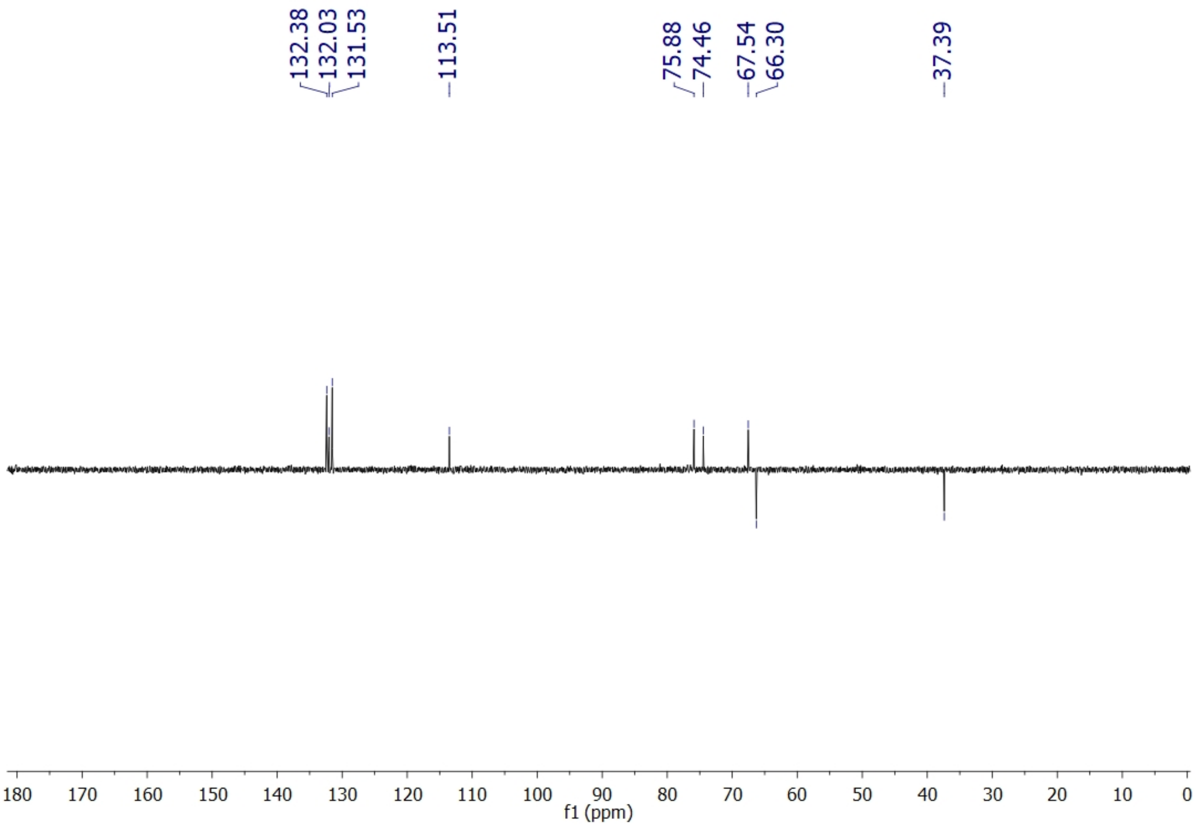

Figure S5. ^{1}H NMR and ^{13}C NMR spectra of 2-Phenyl-1-(prop-2-yn-1-yl)-5-((1*R*,2*S*,3*R*)-1,2,3,4-tetrahydroxybutyl)-1*H*-pyrrole-3-carbonitrile.

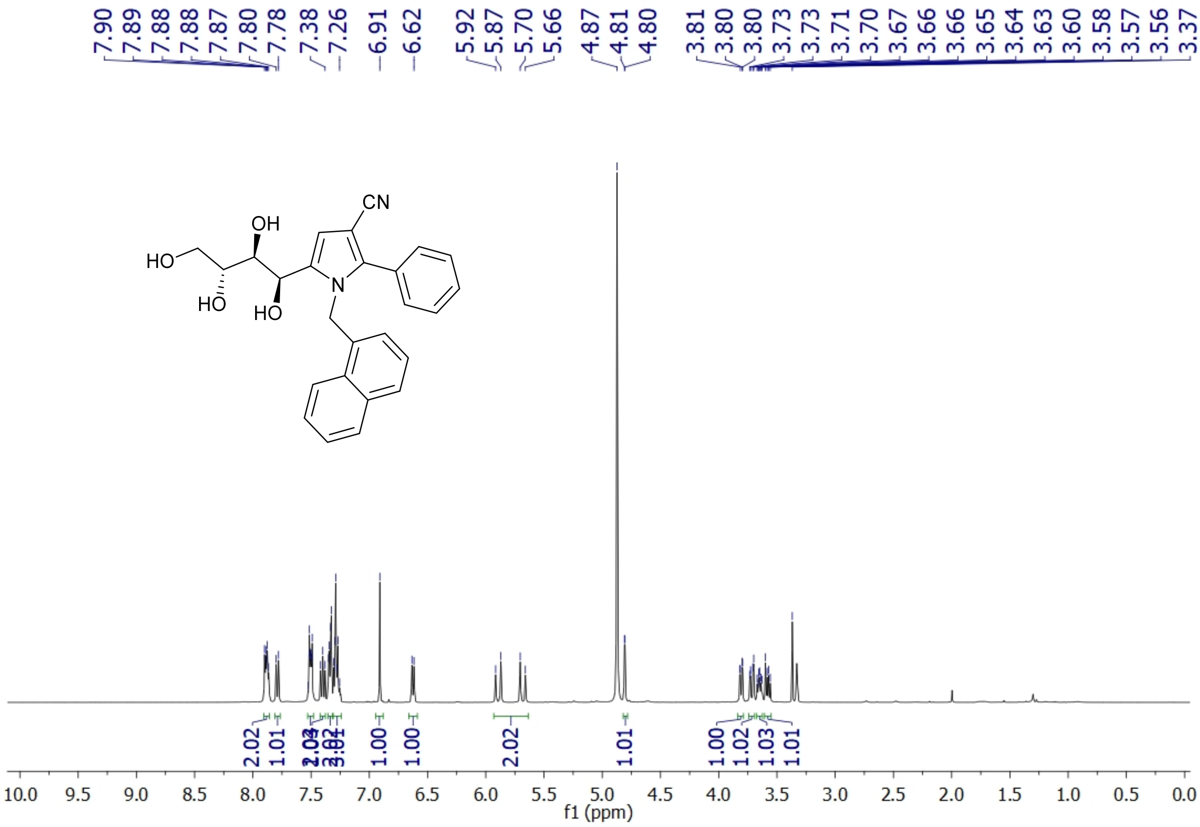
7.90
7.89
7.88
7.88
7.87
7.80
7.78
7.38
7.26
6.91
6.62
5.92
5.87
5.70
5.66
4.87
4.81
4.80
3.81
3.80
3.80
3.73
3.73
3.71
3.70
3.67
3.66
3.66
3.65
3.64
3.63
3.60
3.58
3.57
3.56
3.37
CN
OH
HO
N
HO
HO
2.02
1.01
1.00
1.00
2.02
1.01
1.00
1.02
1.03
1.01
10.0
9.5
9.0
8.5
8.0
7.5
7.0
6.5
6.0
5.5
5.0
4.5
4.0
3.5
3.0
2.5
2.0
1.5
1.0
0.5
0.0
f1 (ppm)

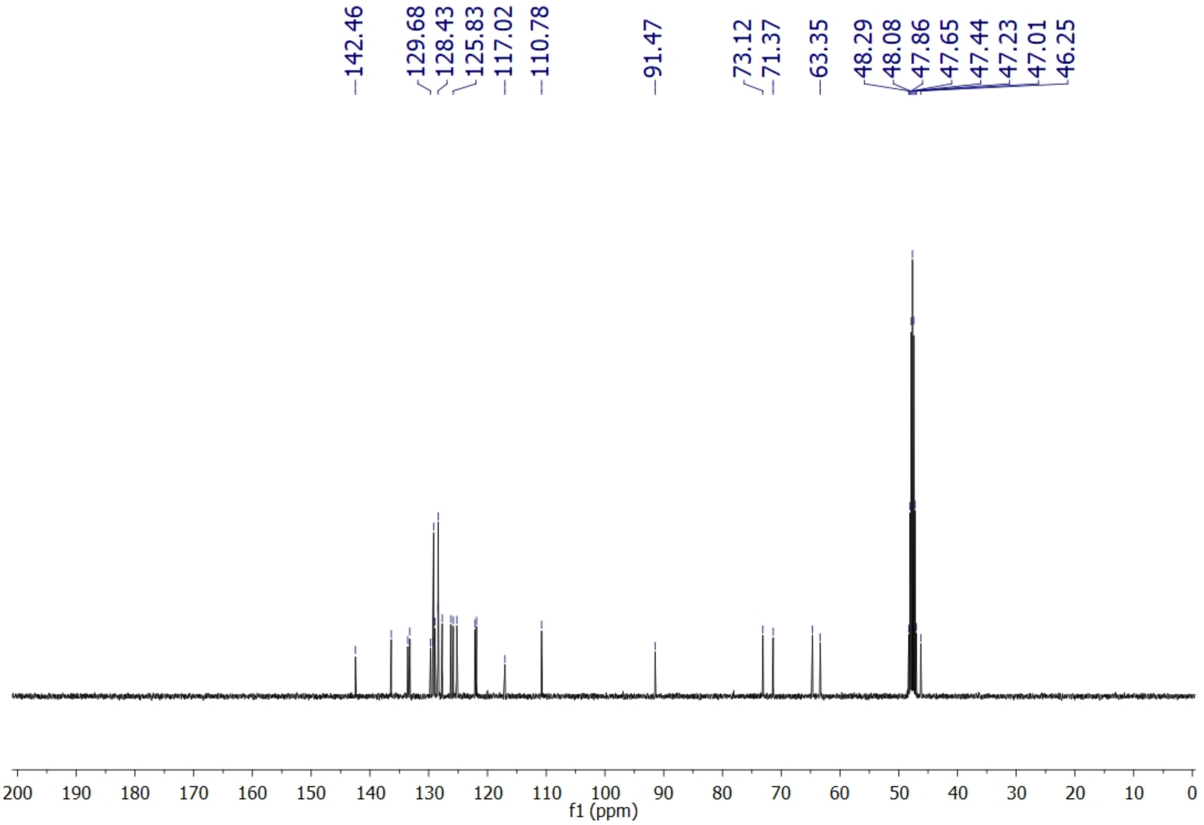
142.46
129.68
128.43
125.83
117.02
110.78
91.47
73.12
71.37
63.35
48.29
48.08
47.86
47.65
47.44
47.23
47.01
46.25
200
190
180
170
160
150
140
130
120
110
100
90
80
70
60
50
40
30
20
10
0
f1 (ppm)

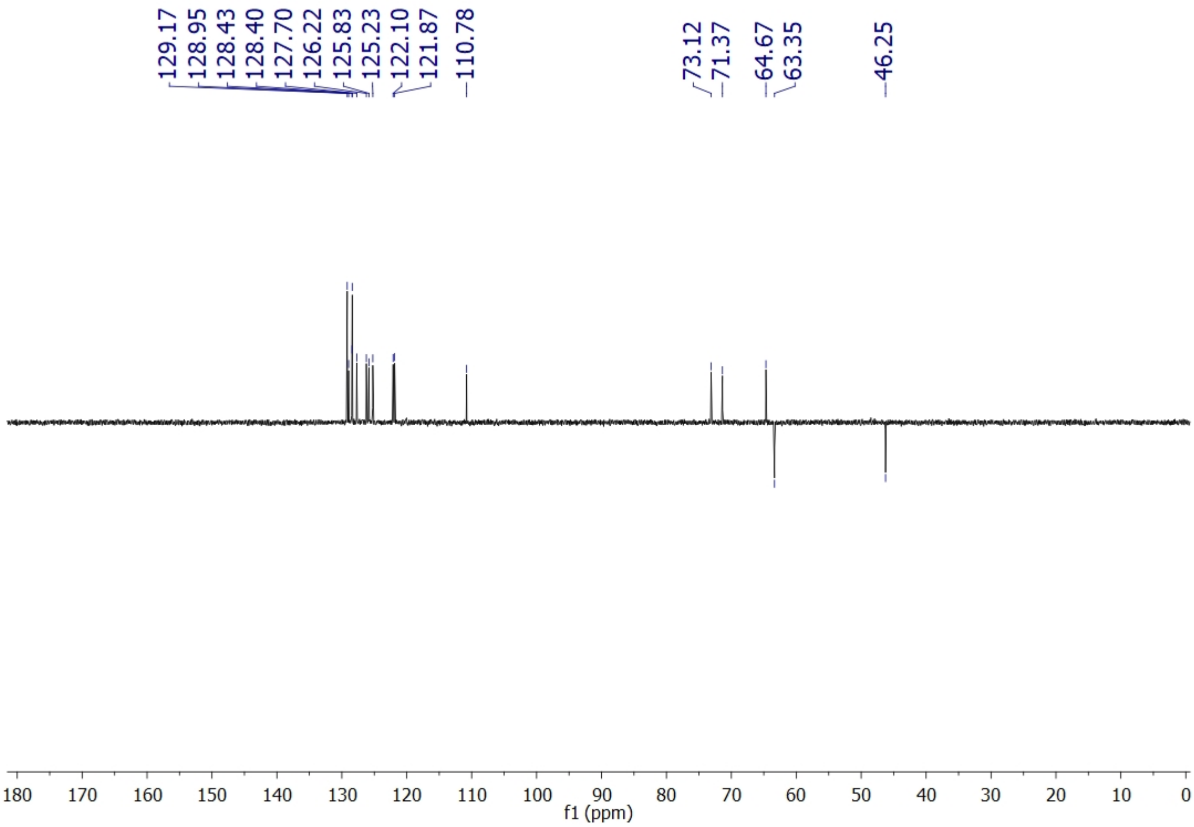

Figure S6. ^{1}H NMR and ^{13}C NMR spectra of 1-(Naphthalen-1-ylmethyl)-2-phenyl-5-((1*R*,2*S*,3*R*)-1,2,3,4-tetrahydroxybutyl)-1*H*-pyrrole-3-carbonitrile.

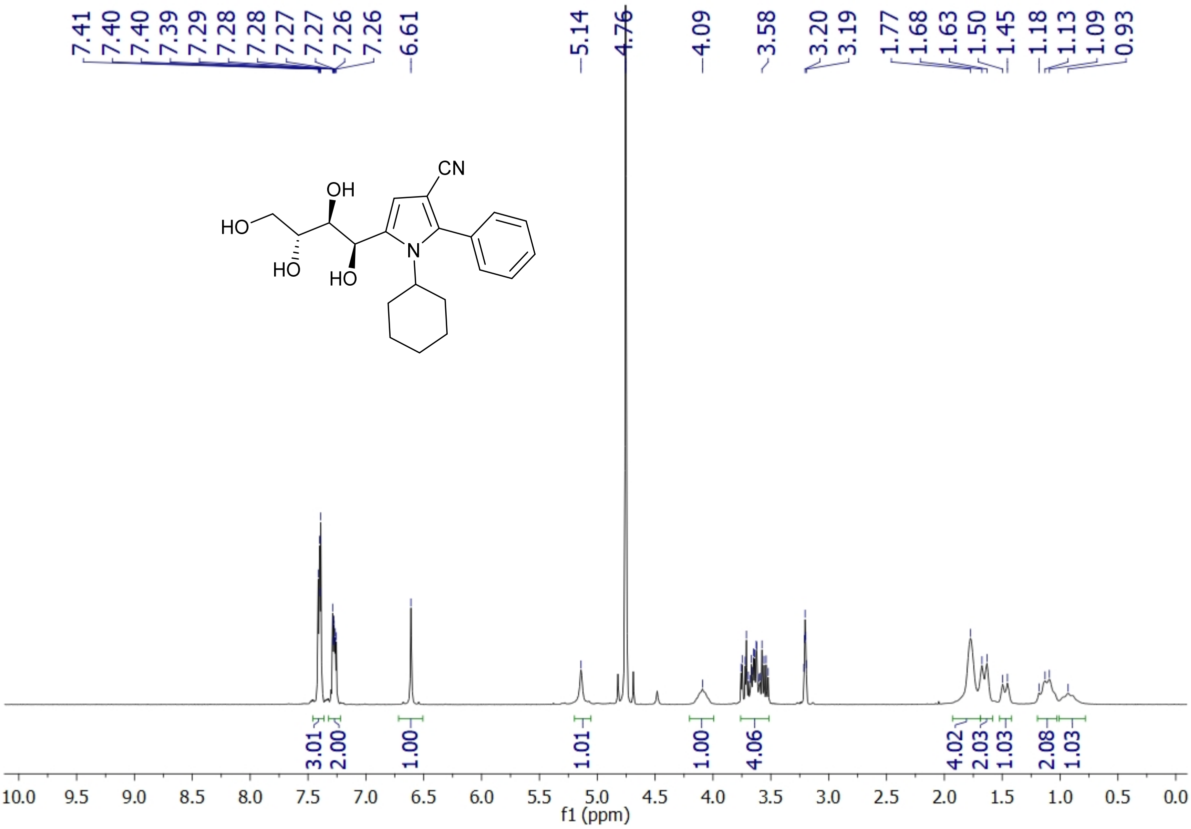
7.41 7.40 7.40 7.39 7.29 7.28 7.28 7.27 7.27 7.26 7.26 6.61 5.14 4.76 4.09 3.58 3.20 3.19 1.77 1.68 1.63 1.50 1.45 1.18 1.13 1.09 0.93
CN
OH
HO
HO
HO
N
3.01 2.00 1.00 1.01 1.00 4.06 4.02 2.03 1.03 2.08 1.03
f1 (ppm)

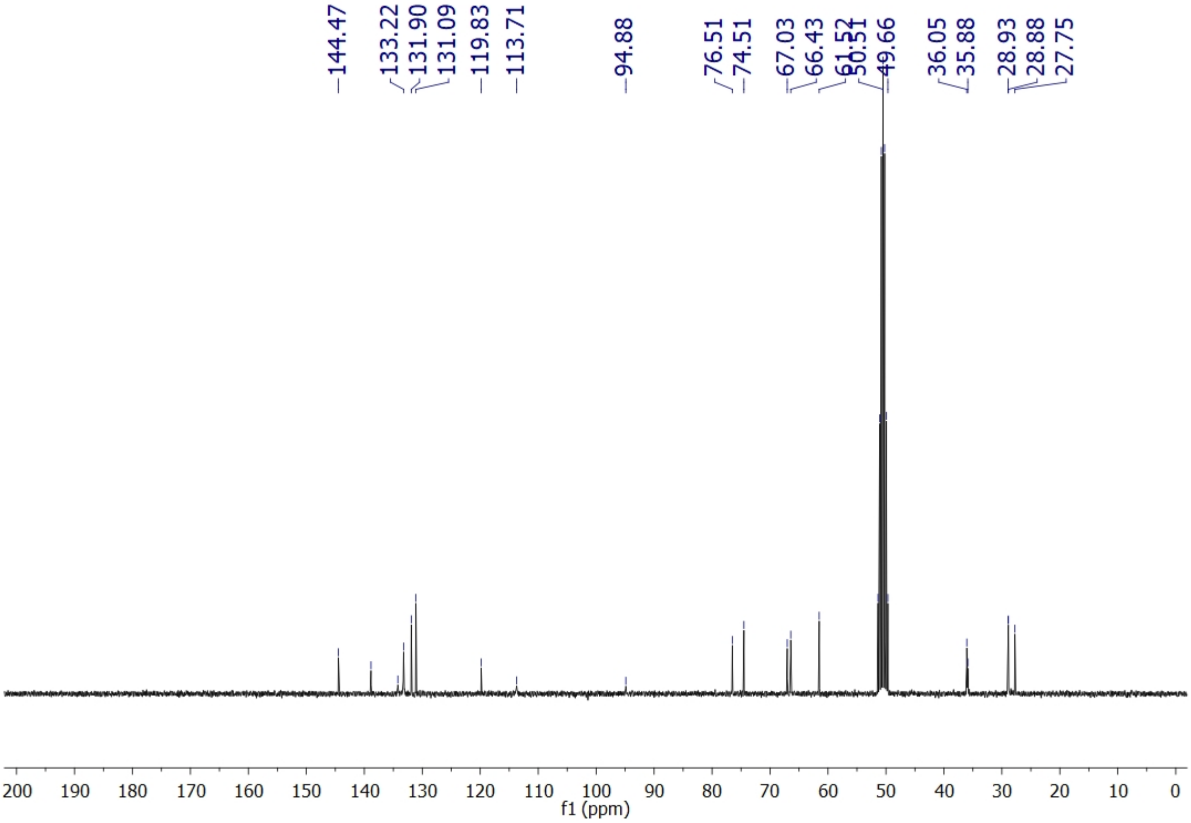
144.47 133.22 131.90 131.09 119.83 113.71 94.88 76.51 74.51 67.03 66.43 61.52 50.51 49.66 36.05 35.88 28.93 28.88 27.75
f1 (ppm)

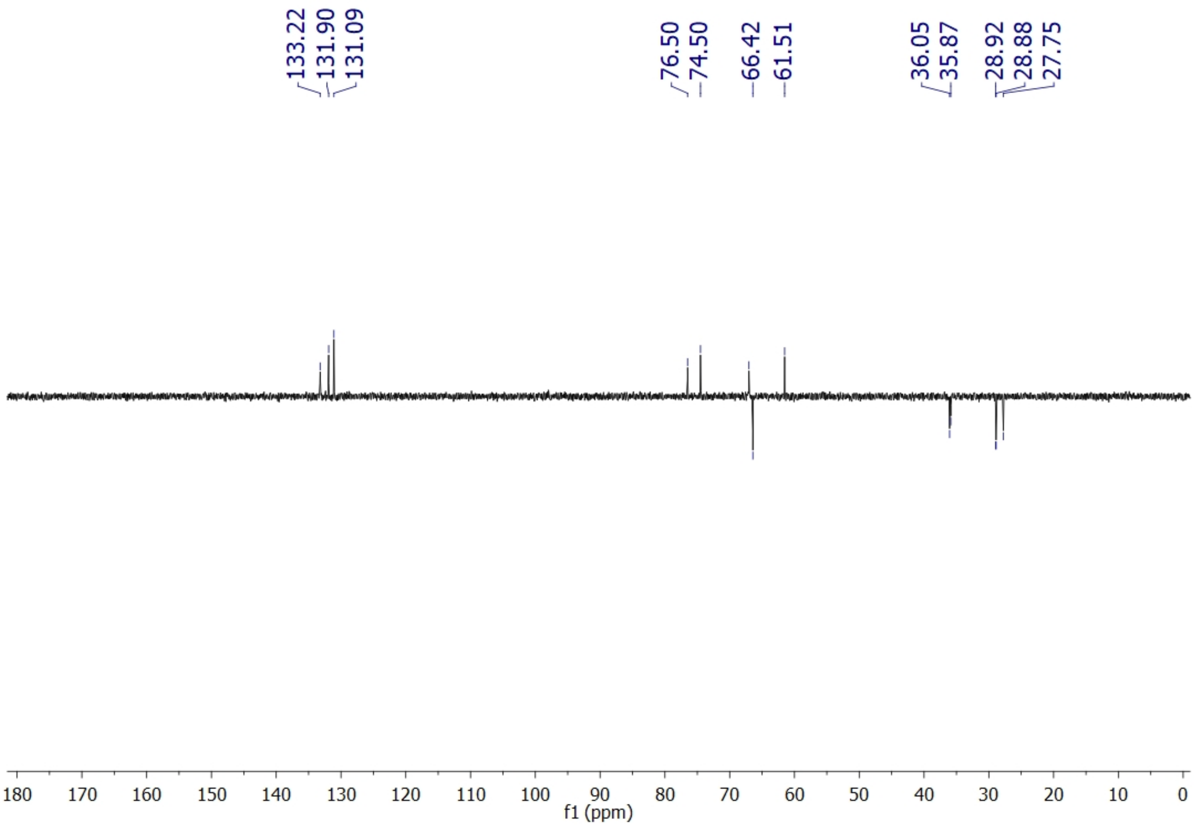

Figure S7. ^{1}H NMR and ^{13}C NMR spectra of 1-Cyclohexyl-2-phenyl-5-((1*R*,2*S*,3*R*)-1,2,3,4-tetrahydroxybutyl)-1*H*-pyrrole-3-carbonitrile.

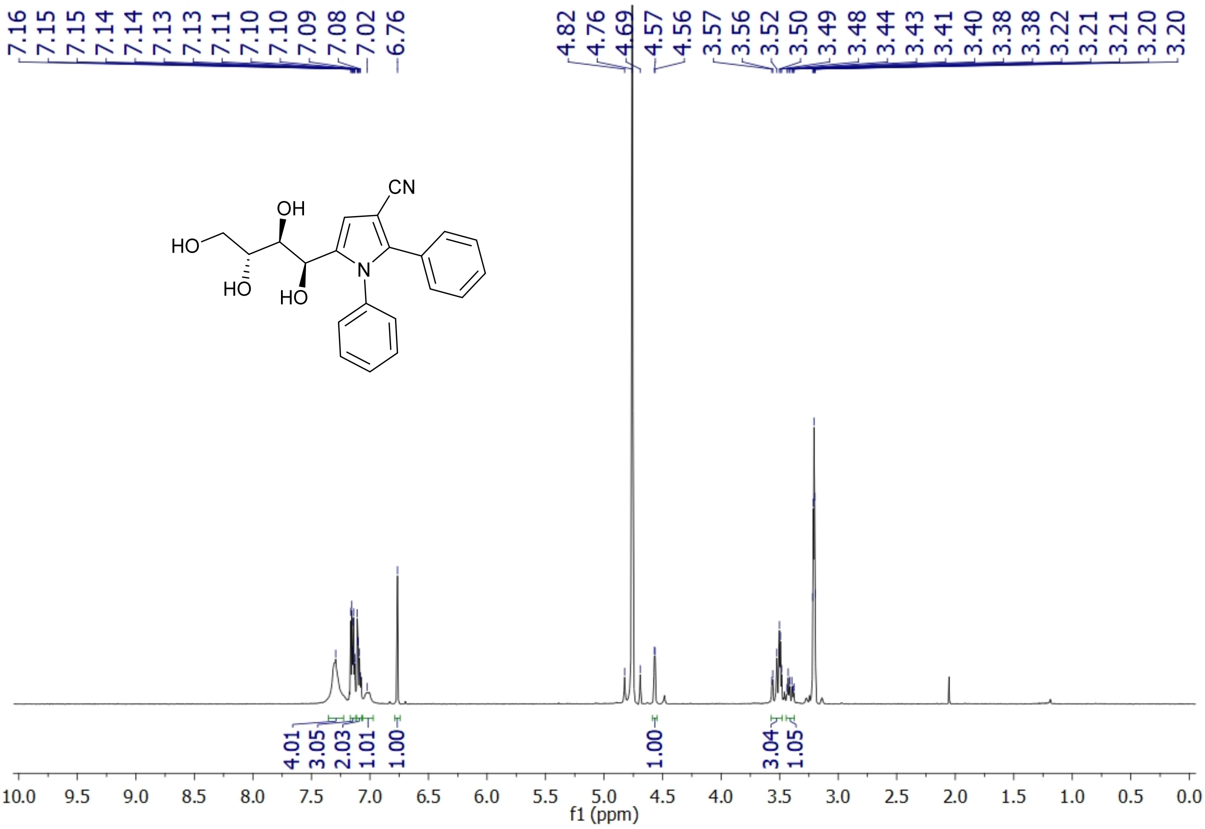
7.16 7.15 7.15 7.14 7.14 7.13 7.13 7.11 7.10 7.10 7.09 7.08 7.02 6.76
4.82 4.76 4.69 4.57 4.56 3.57 3.56 3.52 3.50 3.49 3.48 3.44 3.43 3.41 3.40 3.38 3.38 3.22 3.21 3.21 3.20 3.20
CN
OH
HO
HO
HO
N
4.01
3.05
2.03
1.01
1.00
1.00
3.04
1.05
10.0 9.5 9.0 8.5 8.0 7.5 7.0 6.5 6.0 5.5 5.0 4.5 4.0 3.5 3.0 2.5 2.0 1.5 1.0 0.5 0.0
f1 (ppm)

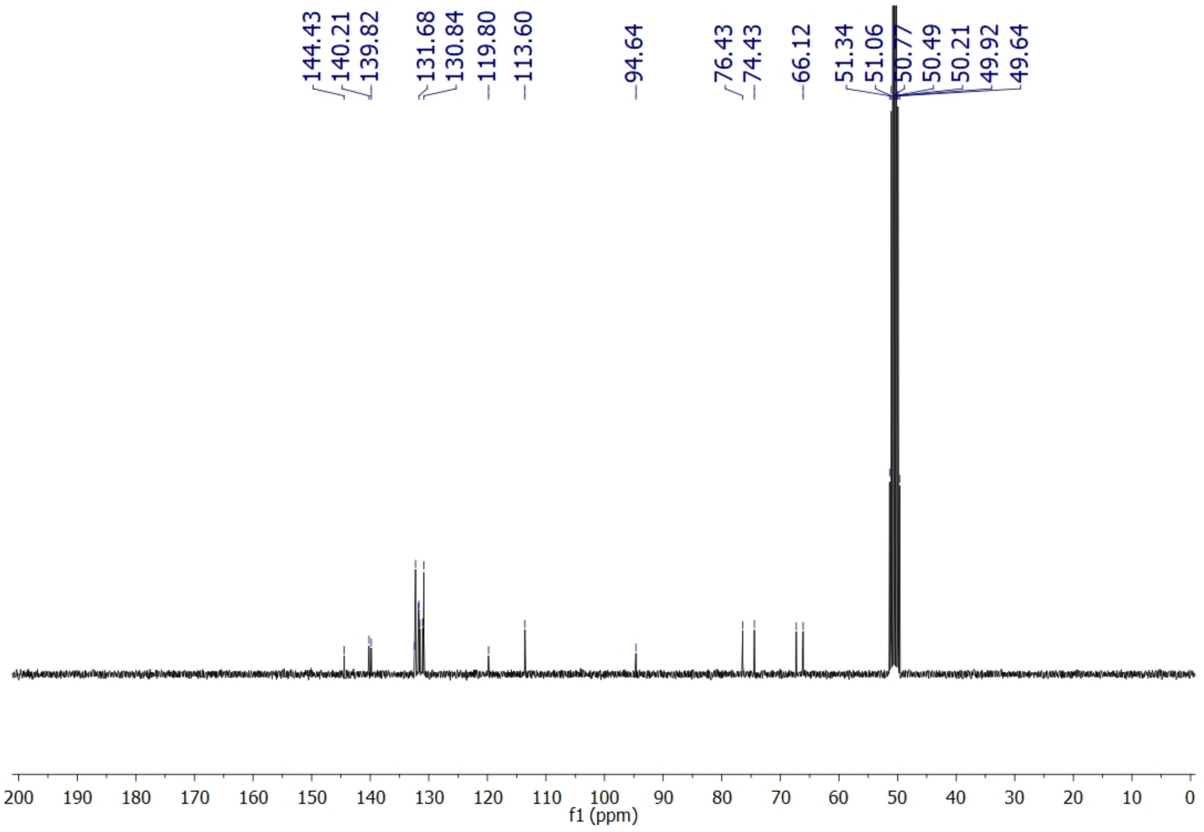
144.43 140.21 139.82 131.68 130.84 119.80 113.60
94.64
76.43 74.43 66.12 51.34 51.06 50.77 50.49 50.21 49.92 49.64
200 190 180 170 160 150 140 130 120 110 100 90 80 70 60 50 40 30 20 10 0
f1 (ppm)

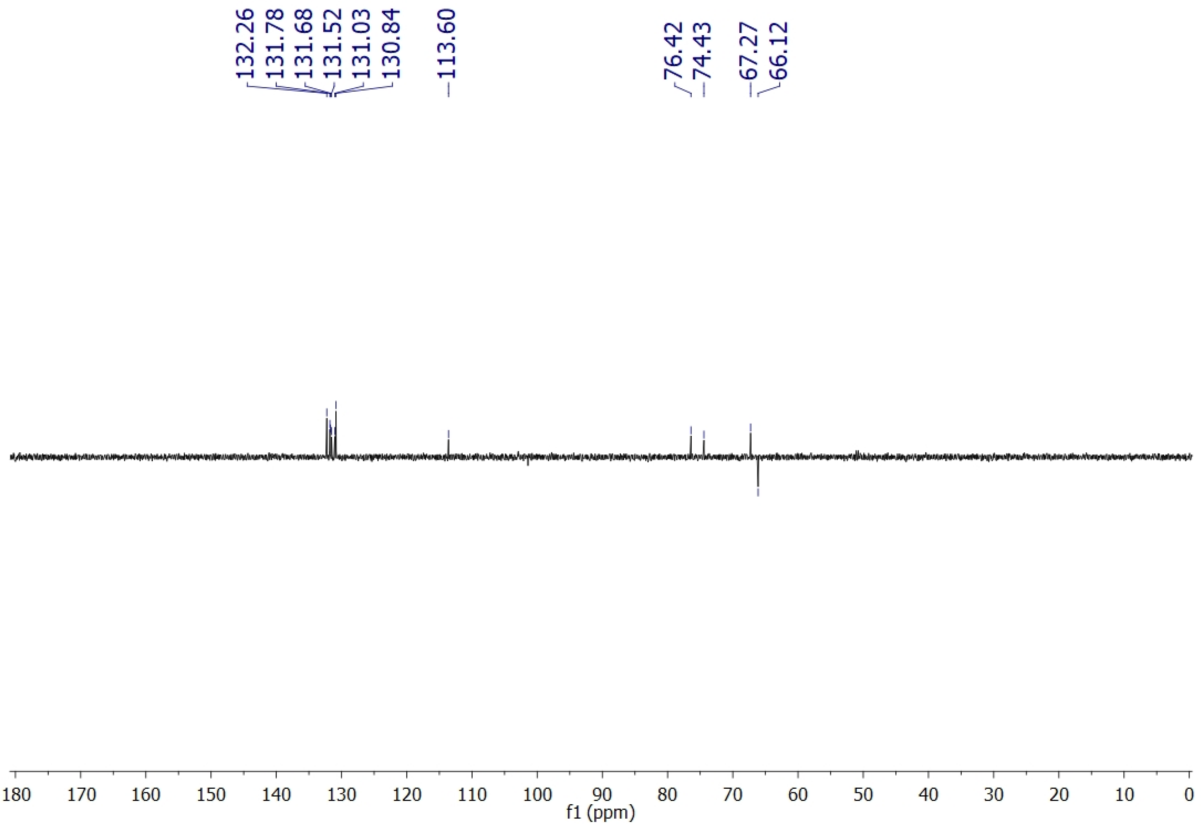

Figure S8. ^{1}H NMR and ^{13}C NMR spectra of 1,2-Diphenyl-5-((1*R*,2*S*,3*R*)-1,2,3,4-tetrahydroxybutyl)-1*H*-pyrrole-3-carbonitrile.

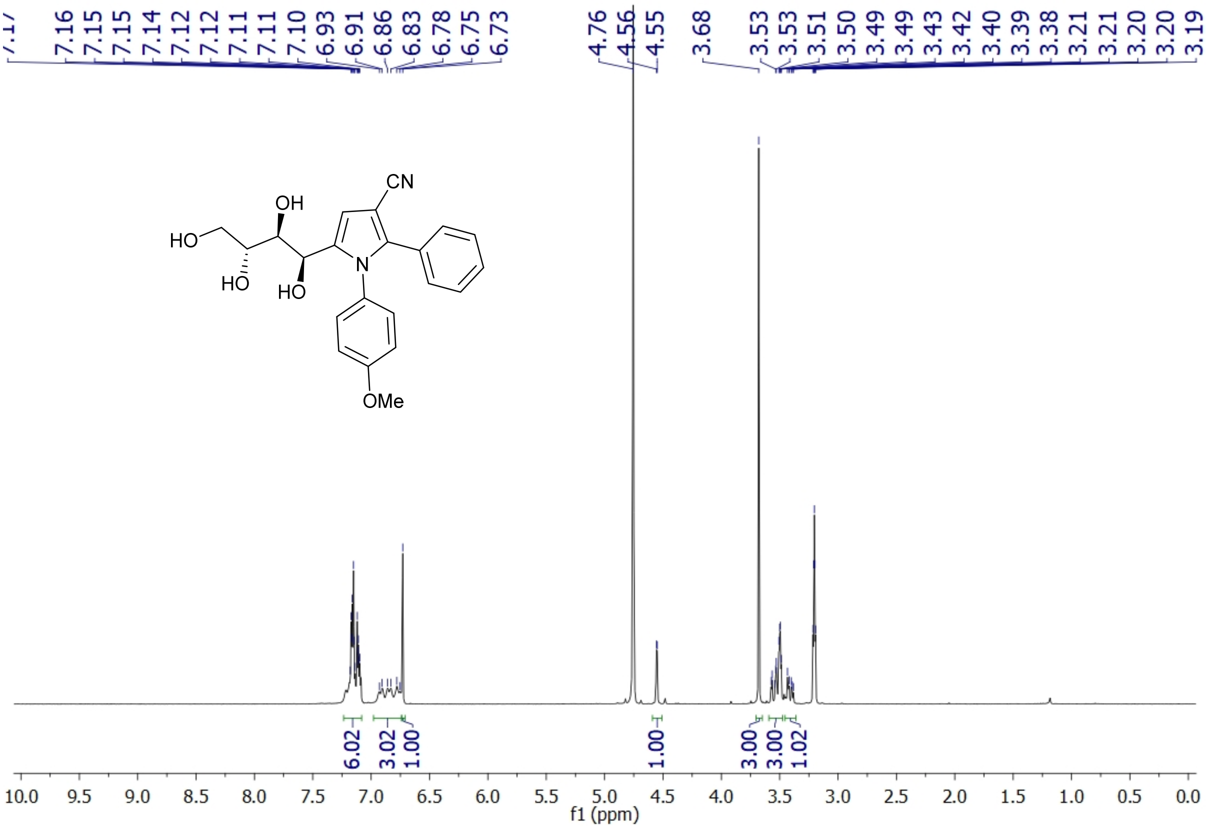
7.16
7.15
7.15
7.14
7.12
7.12
7.11
7.11
7.10
6.93
6.91
6.86
6.83
6.78
6.75
6.73
4.76
4.56
4.55
3.68
3.53
3.53
3.51
3.50
3.49
3.49
3.43
3.42
3.40
3.39
3.38
3.21
3.21
3.20
3.20
3.19
CN
OH
HO
HO
HO
N
OMe
6.02
3.02
1.00
1.00
3.00
3.00
1.02
10.0 9.5 9.0 8.5 8.0 7.5 7.0 6.5 6.0 5.5 5.0 4.5 4.0 3.5 3.0 2.5 2.0 1.5 1.0 0.5 0.0
f1 (ppm)

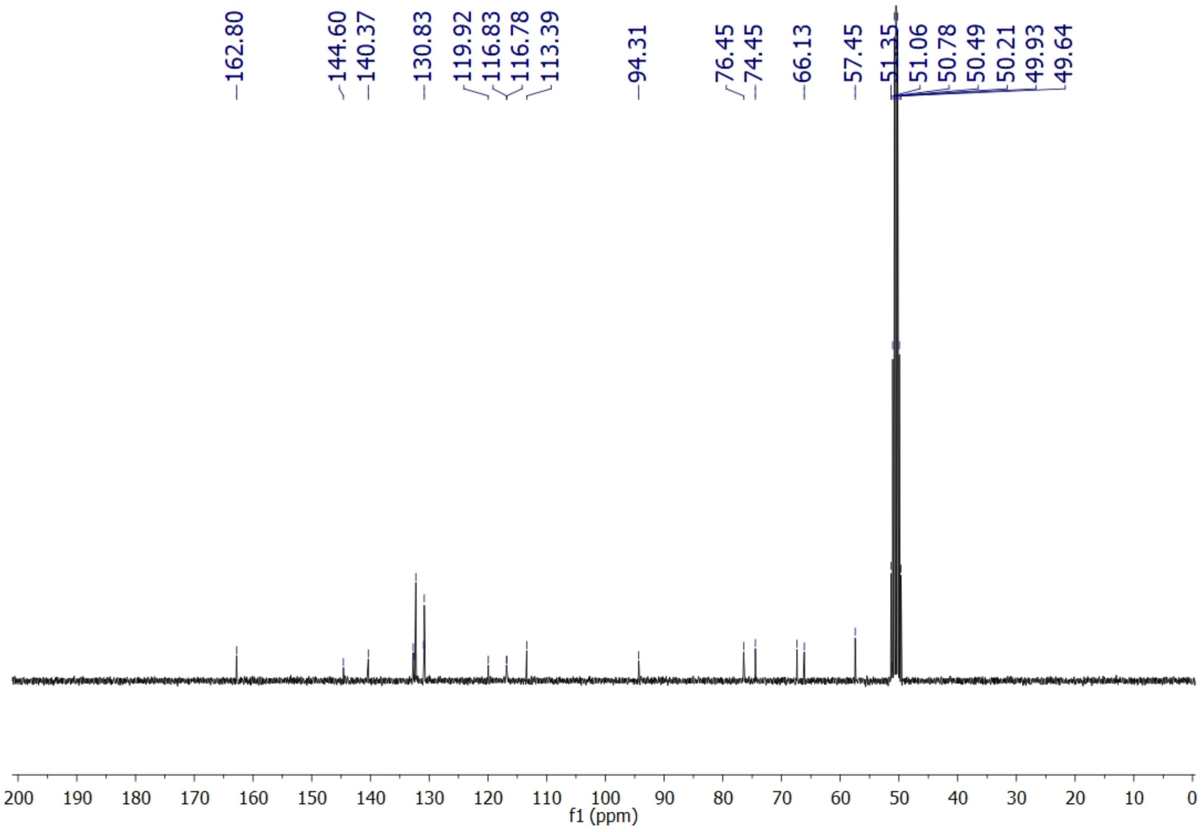
162.80
144.60
140.37
130.83
119.92
116.83
116.78
113.39
94.31
76.45
74.45
66.13
57.45
51.35
51.06
50.78
50.49
50.21
49.93
49.64
200 190 180 170 160 150 140 130 120 110 100 90 80 70 60 50 40 30 20 10 0
f1 (ppm)

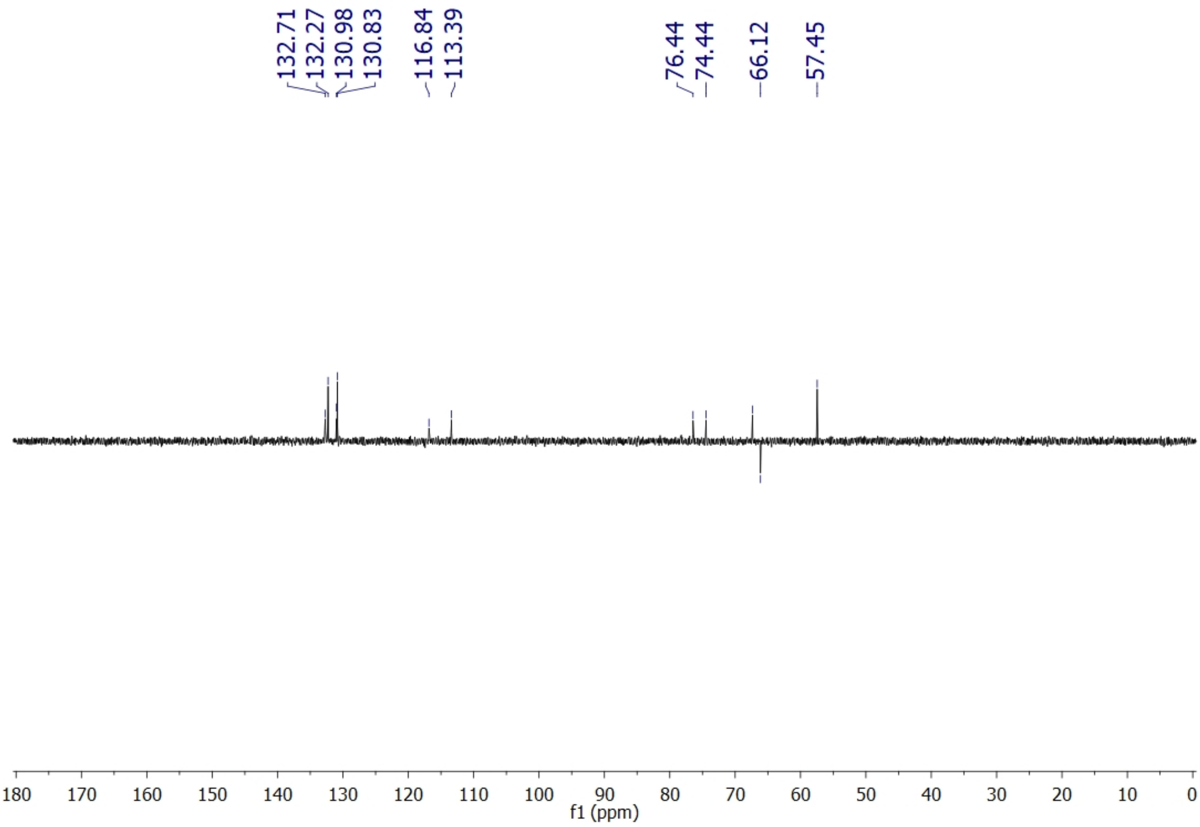

Figure S9. ^{1}H NMR and ^{13}C NMR spectra of 1-(4-Methoxyphenyl)-2-phenyl-5-((1*R*,2*S*,3*R*)-1,2,3,4-tetrahydroxybutyl)-1*H*-pyrrole-3-carbonitrile.

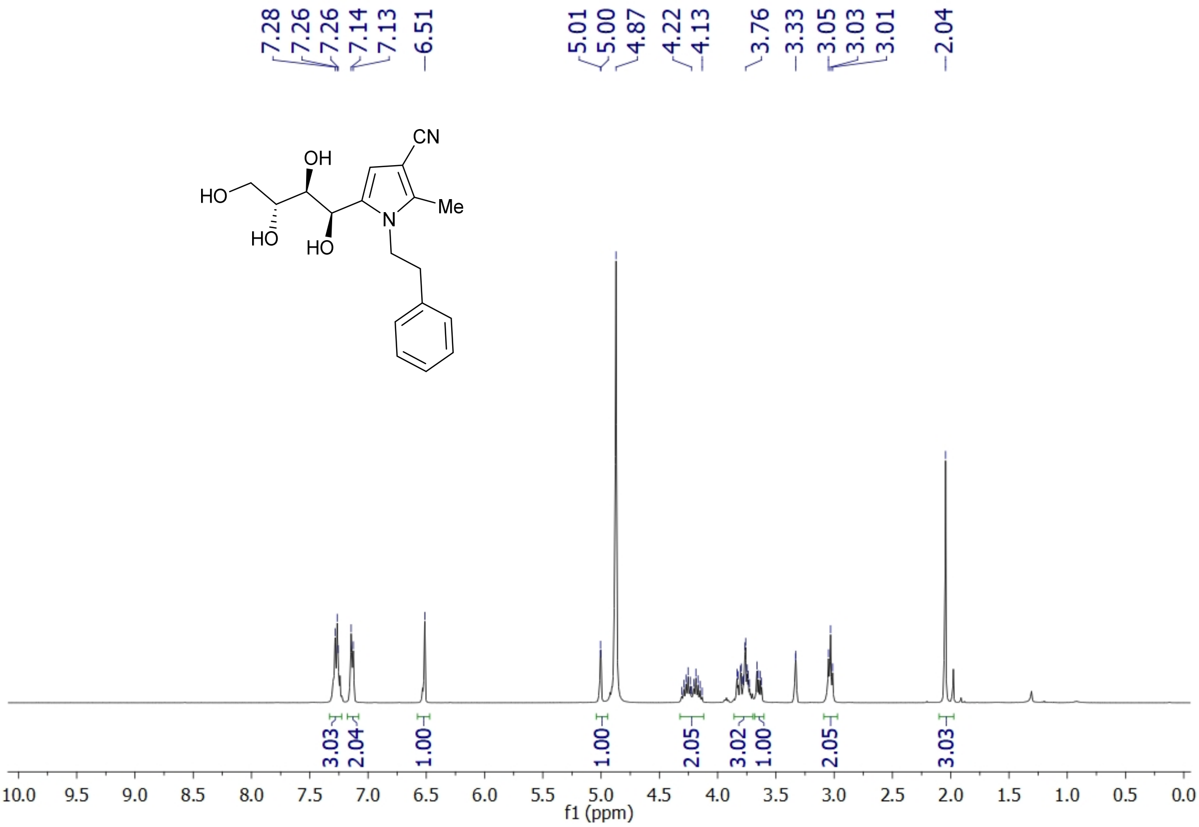
7.28
7.26
7.26
7.14
7.13
6.51
5.01
5.00
4.87
4.22
4.13
3.76
3.33
3.05
3.03
3.01
2.04
CN
OH
HO
Me
N
HO
HO
3.03
2.04
1.00
1.00
2.05
3.02
1.00
2.05
3.03
f1 (ppm)

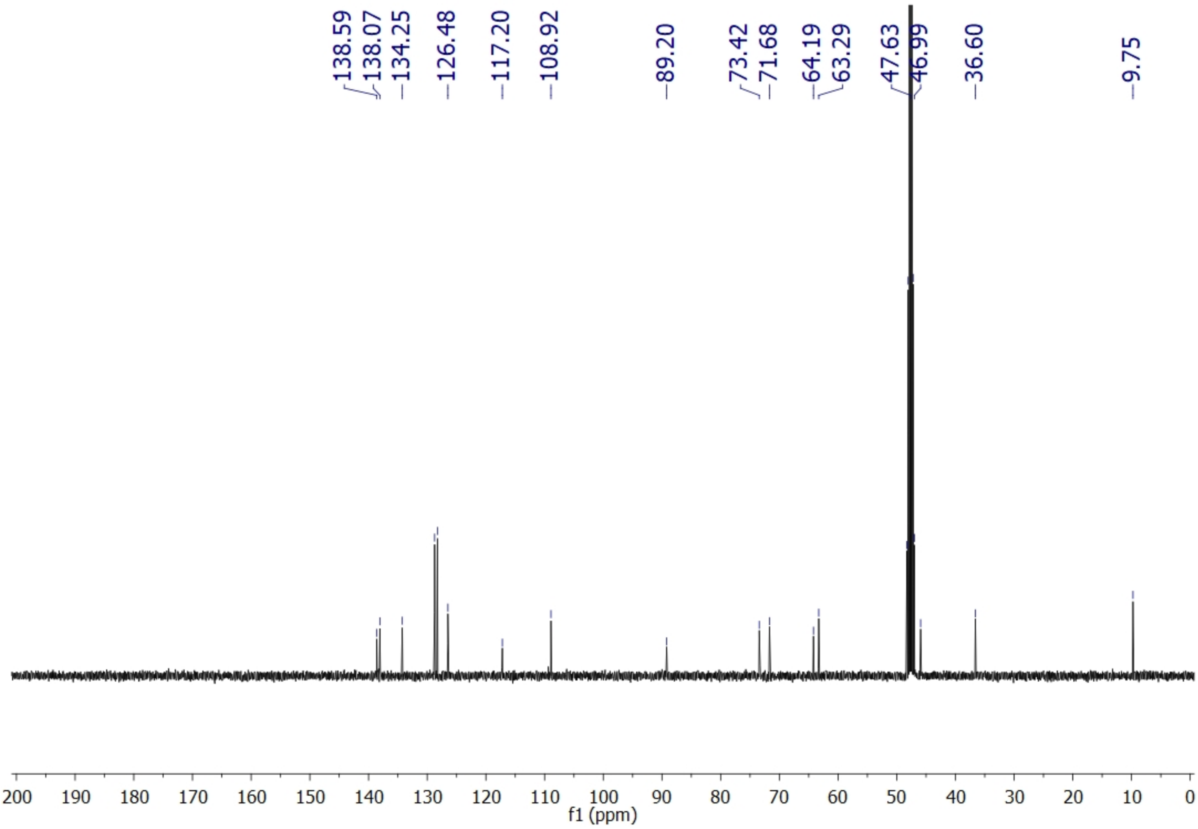
138.59
138.07
134.25
126.48
117.20
108.92
89.20
73.42
71.68
64.19
63.29
47.63
46.99
36.60
9.75
f1 (ppm)

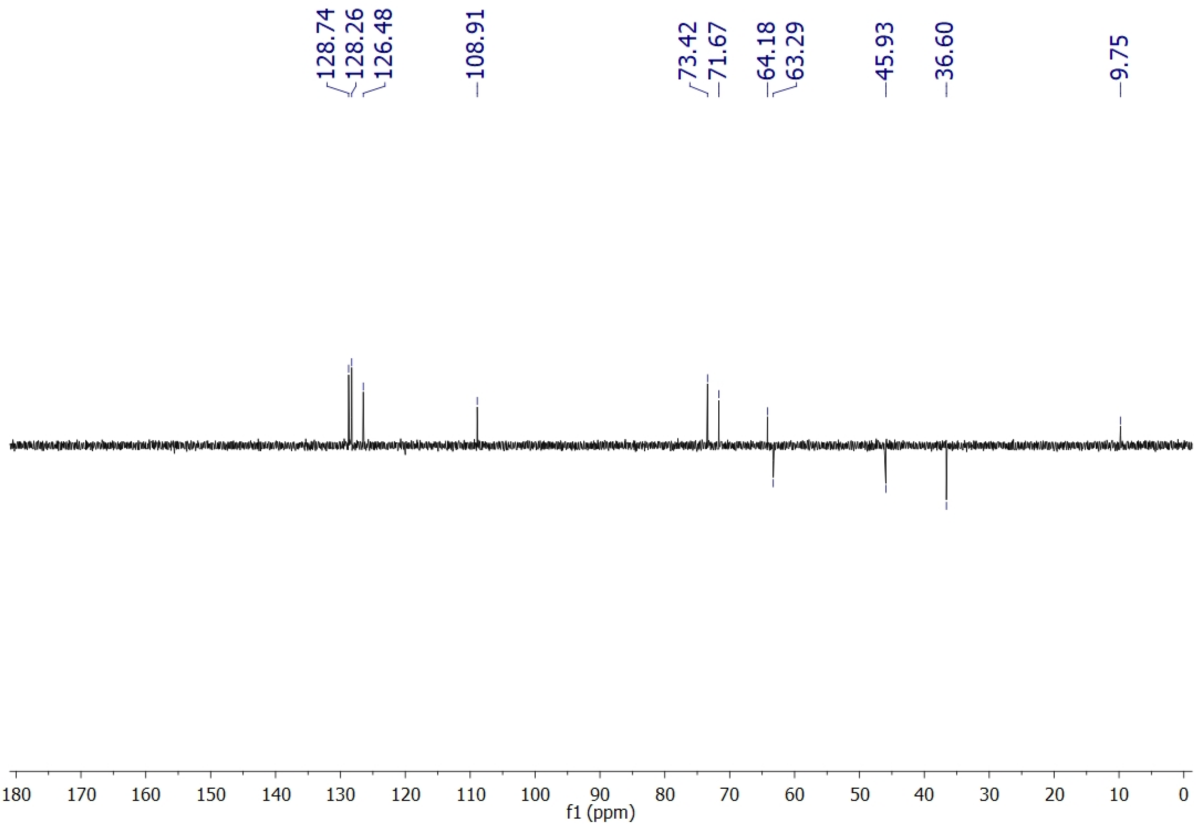

Figure S10. ^{1}H NMR and ^{13}C NMR spectra of 2-Methyl-1-phenethyl-5-((1*R*,2*S*,3*R*)-1,2,3,4-tetrahydroxybutyl)-1*H*-pyrrole-3-carbonitrile.

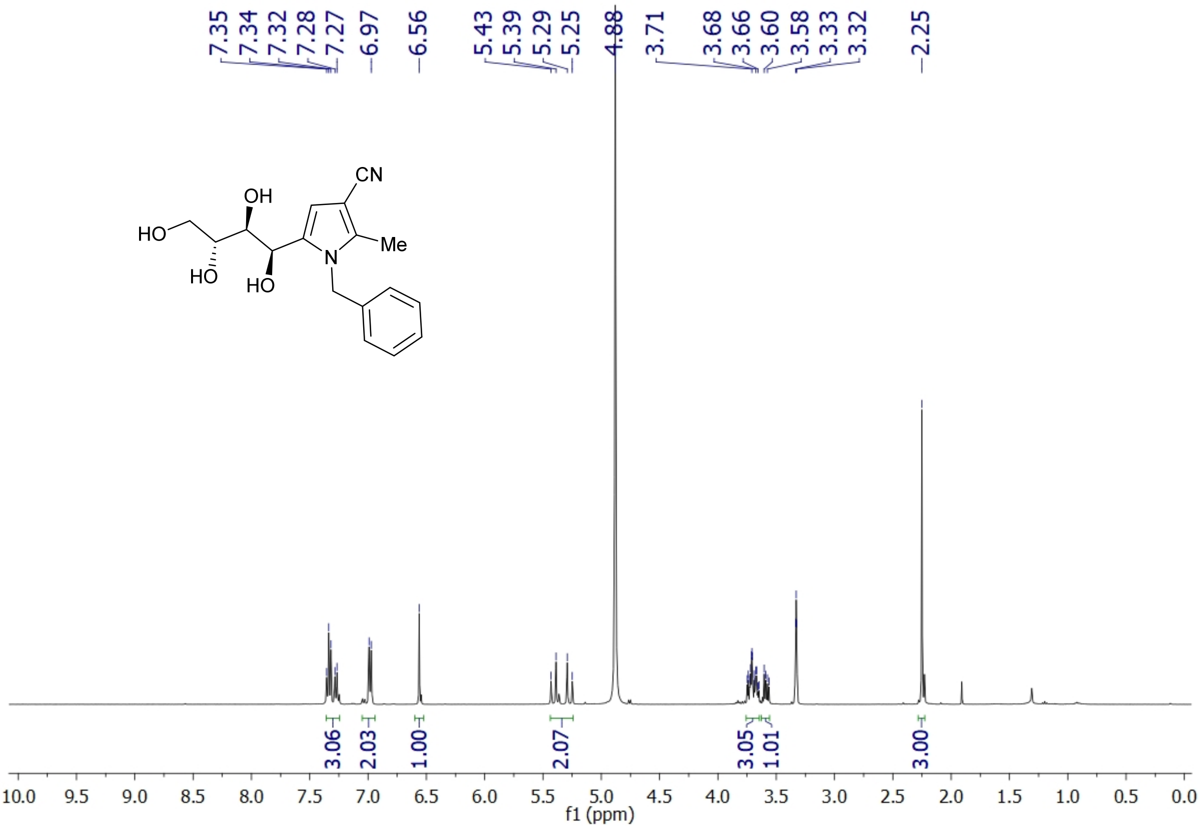
7.35
7.34
7.32
7.28
7.27
6.97
6.56
5.43
5.39
5.29
5.25
4.88
3.71
3.68
3.66
3.60
3.58
3.33
3.32
2.25
CN
OH
HO
Me
N
HO
HO
3.06
2.03
1.00
2.07
3.05
1.01
3.00
10.0 9.5 9.0 8.5 8.0 7.5 7.0 6.5 6.0 5.5 5.0 4.5 4.0 3.5 3.0 2.5 2.0 1.5 1.0 0.5 0.0
f1 (ppm)

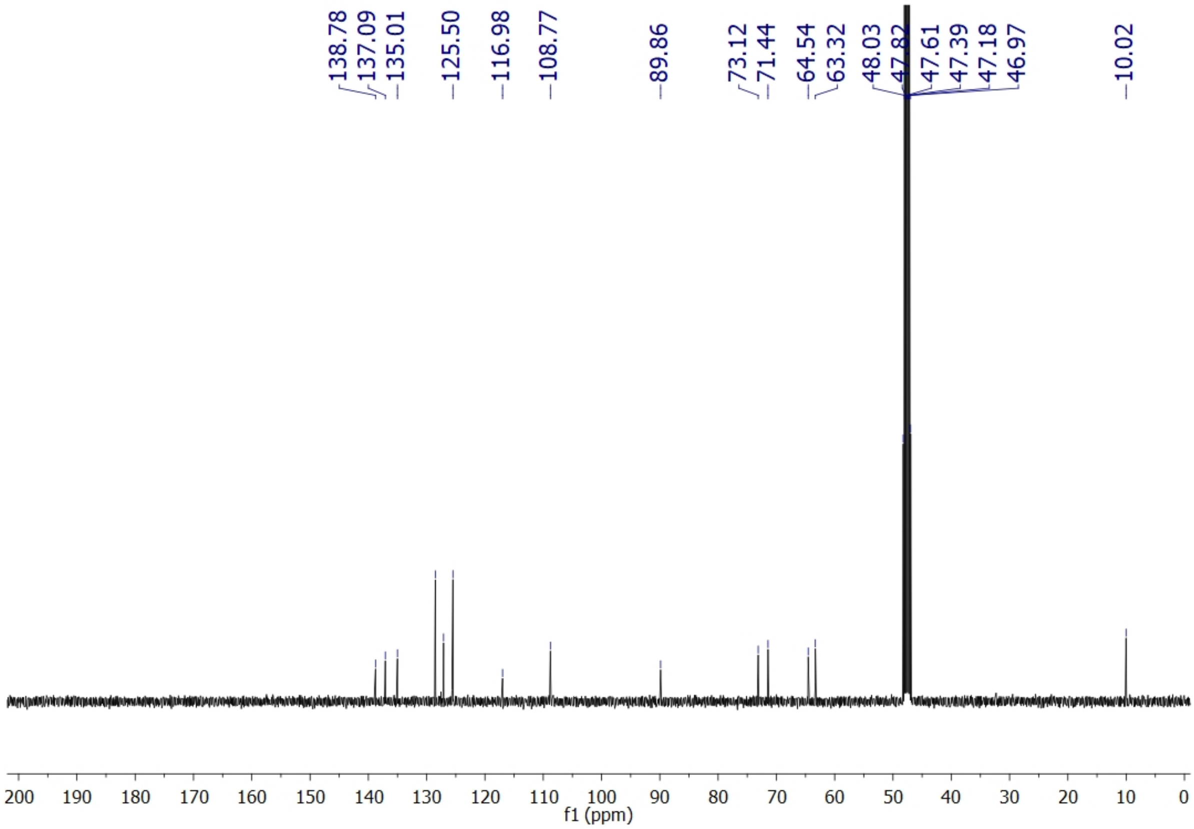
138.78
137.09
135.01
125.50
116.98
108.77
89.86
73.12
71.44
64.54
63.32
48.03
47.82
47.61
47.39
47.18
46.97
10.02
200 190 180 170 160 150 140 130 120 110 100 90 80 70 60 50 40 30 20 10 0
f1 (ppm)

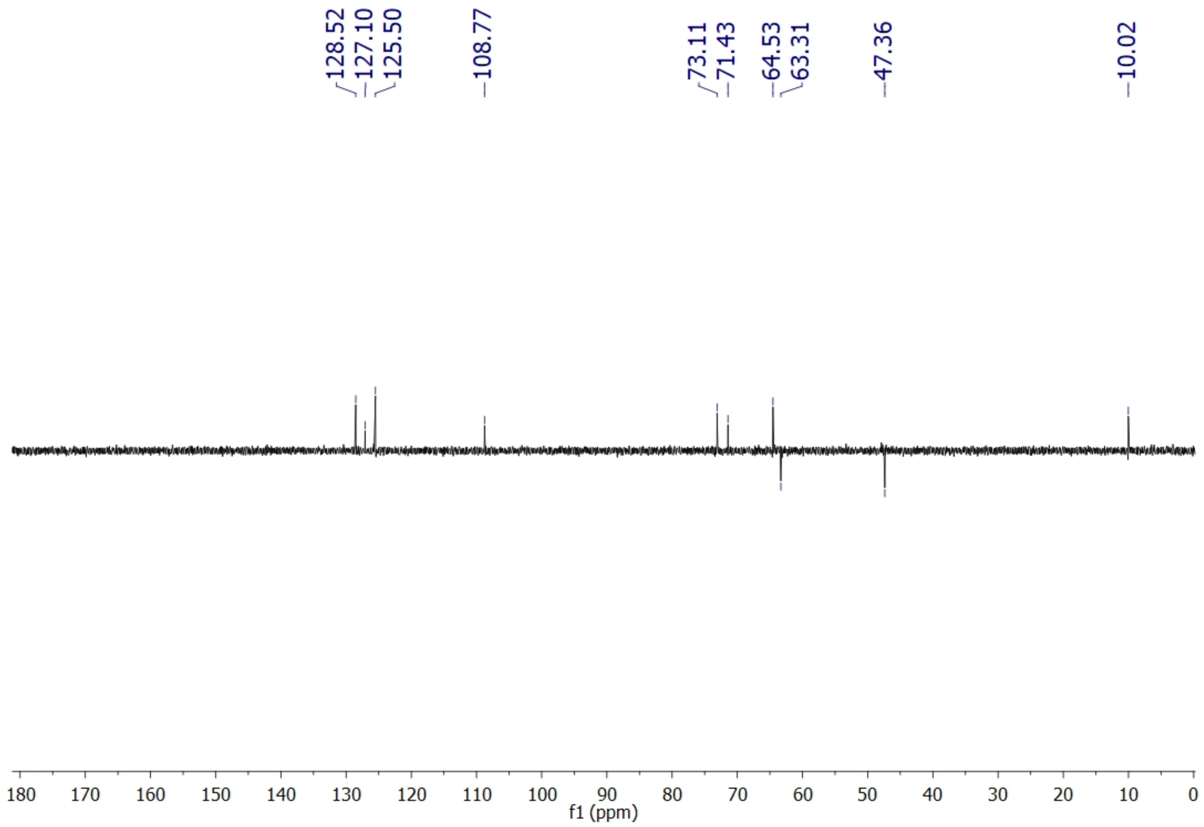

Figure S11. ^{1}H NMR and ^{13}C NMR spectra of 1-Benzyl-2-methyl-5-((1*R*,2*S*,3*R*)-1,2,3,4-tetrahydroxybutyl)-1*H*-pyrrole-3-carbonitrile.

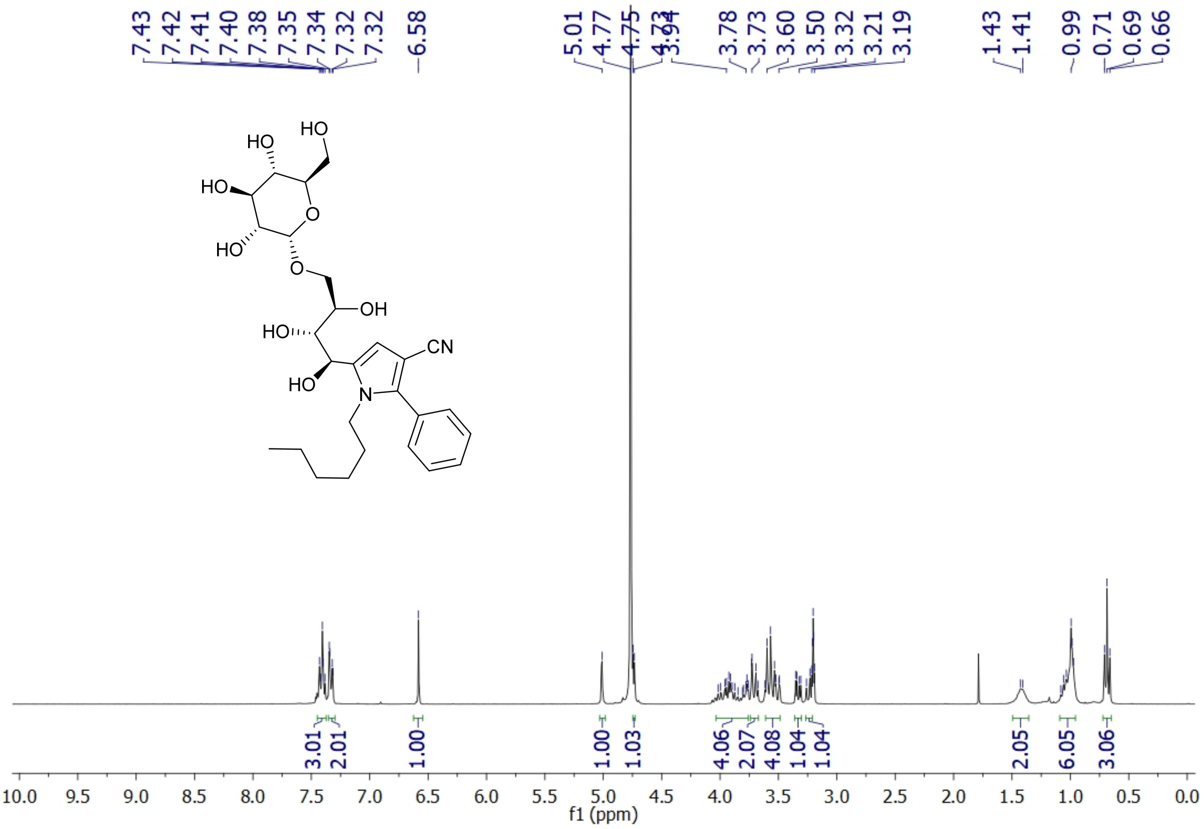
7.43 7.42 7.41 7.40 7.38 7.35 7.34 7.32 7.32 6.58
5.01 4.77 4.75 4.73 3.94 3.78 3.73 3.60 3.50 3.32 3.21 3.19
1.43 1.41 0.99 0.71 0.69 0.66
HO HO HO HO O O OH HO HO CN N
3.01 2.01 1.00 1.00 1.03 4.06 2.07 4.08 1.04 1.04 2.05 6.05 3.06
f1 (ppm)

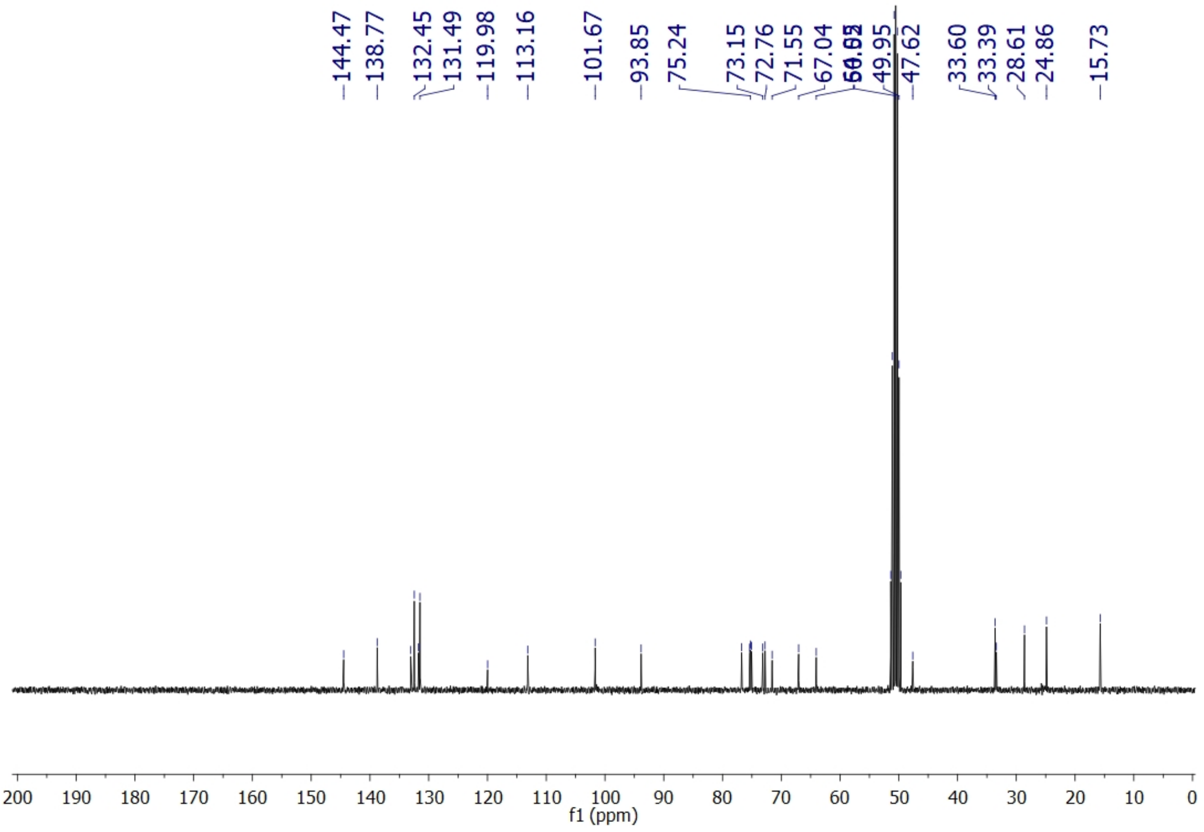
144.47 138.77 132.45 131.49 119.98 113.16 101.67 93.85 75.24 73.15 72.76 71.55 67.04 49.95 47.62 33.60 33.39 28.61 24.86 15.73
f1 (ppm)

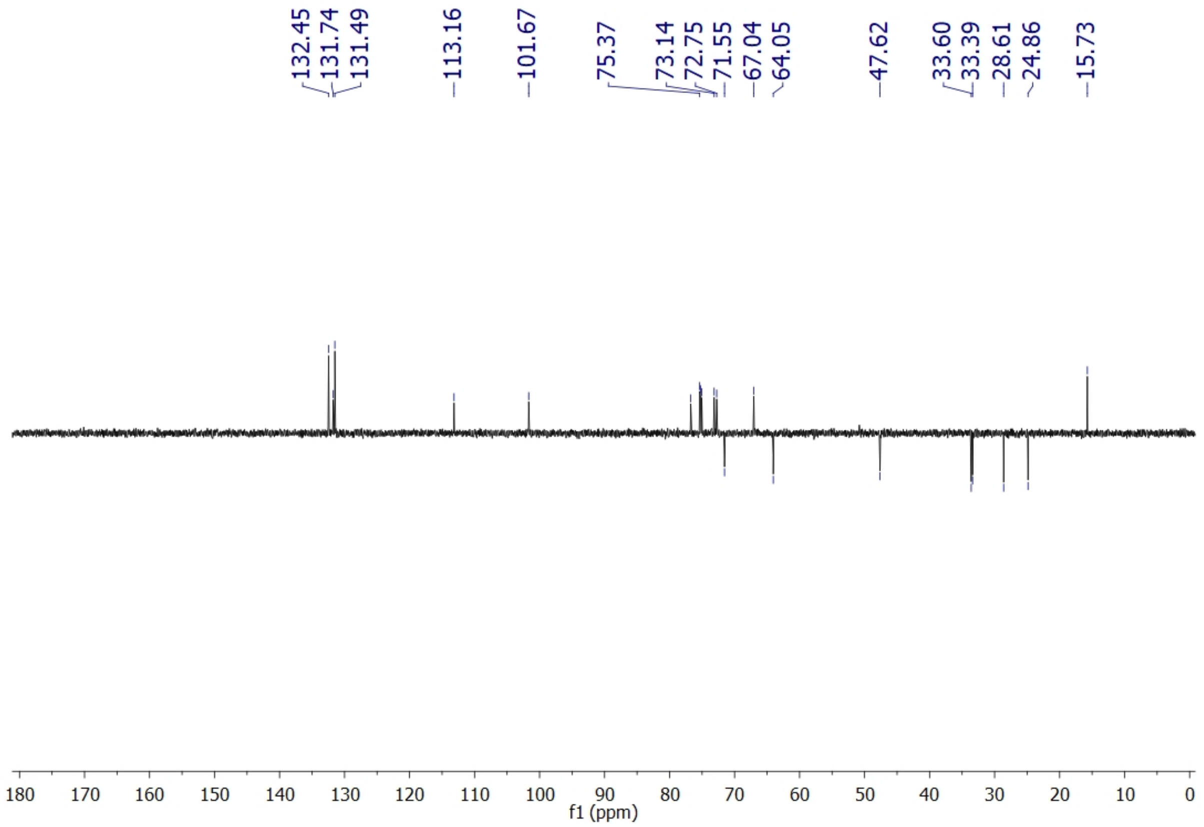

Figure S12. ^{1}H NMR and ^{13}C NMR spectra of 1-Hexyl-2-phenyl-5-((1*R*,2*S*,3*R*)-1,2,3-trihydroxy-4-(((2*S*,3*R*,4*S*,5*S*,6*R*)-3,4,5-trihydroxy-6-(hydroxymethyl)tetrahydro-2*H*-pyran-2-yl)oxy)butyl)-1*H*-pyrrole-3-carbonitrile.

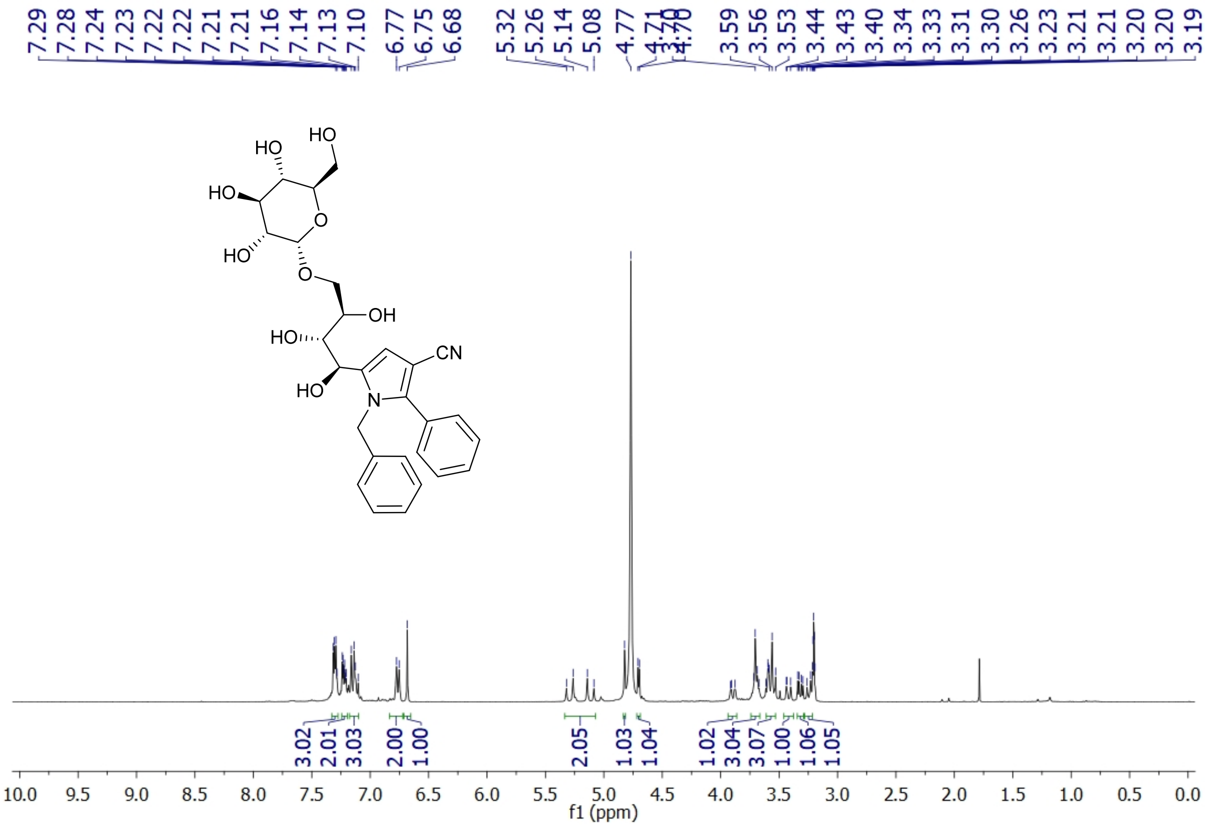
7.29 7.28 7.24 7.23 7.22 7.22 7.21 7.21 7.16 7.14 7.13 7.10
6.77 6.75 6.68
5.32 5.26 5.14 5.08
4.77 4.71 4.70
3.59 3.56 3.53 3.44 3.43 3.40 3.34 3.33 3.31 3.30 3.26 3.23 3.21 3.21 3.20 3.20 3.19
HO
HO
HO
HO
O
O
OH
HO
HO
N
CN
3.02 2.01 3.03 2.00 1.00
2.05
1.03 1.04
1.02 3.04 3.07 1.00 1.06 1.05
10.0 9.5 9.0 8.5 8.0 7.5 7.0 6.5 6.0 5.5 5.0 4.5 4.0 3.5 3.0 2.5 2.0 1.5 1.0 0.5 0.0
f1 (ppm)

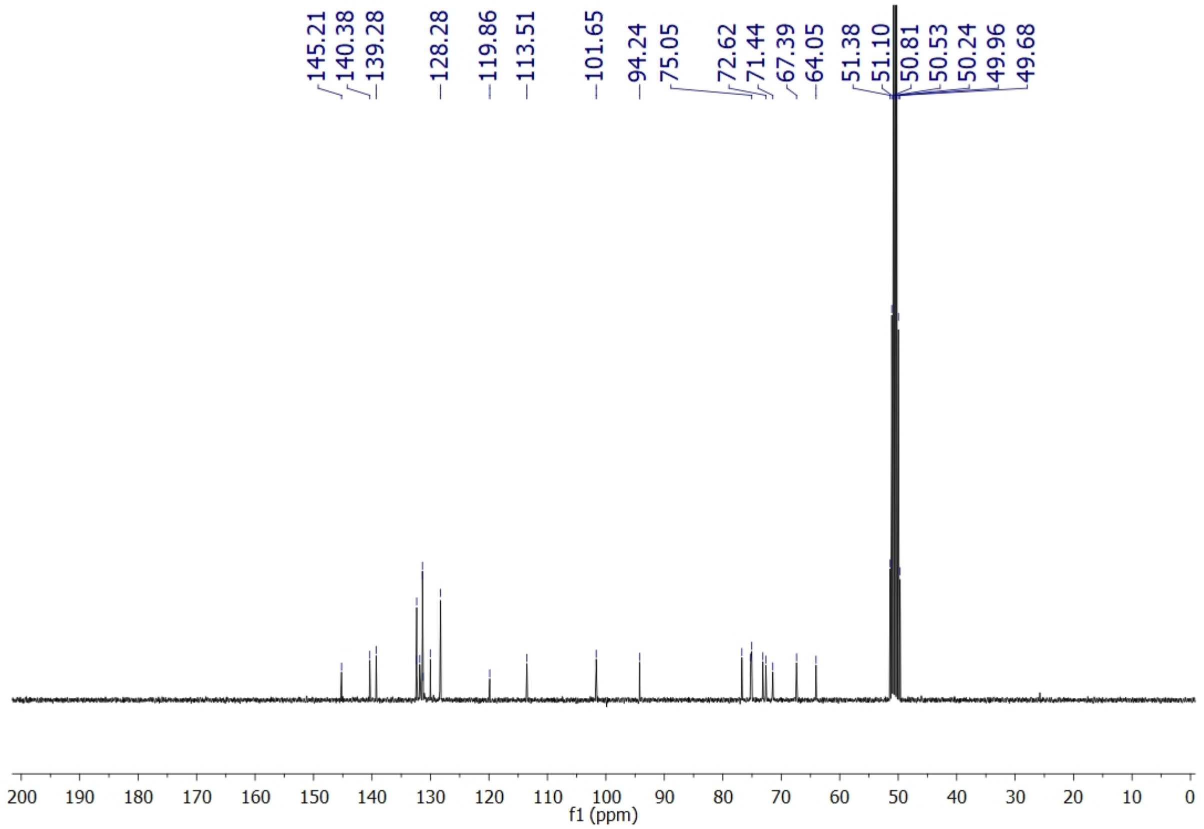
145.21 140.38 139.28
128.28
119.86
113.51
101.65
94.24
75.05
72.62 71.44 67.39 64.05
51.38 51.10 50.81 50.53 50.24 49.96 49.68
200 190 180 170 160 150 140 130 120 110 100 90 80 70 60 50 40 30 20 10 0
f1 (ppm)

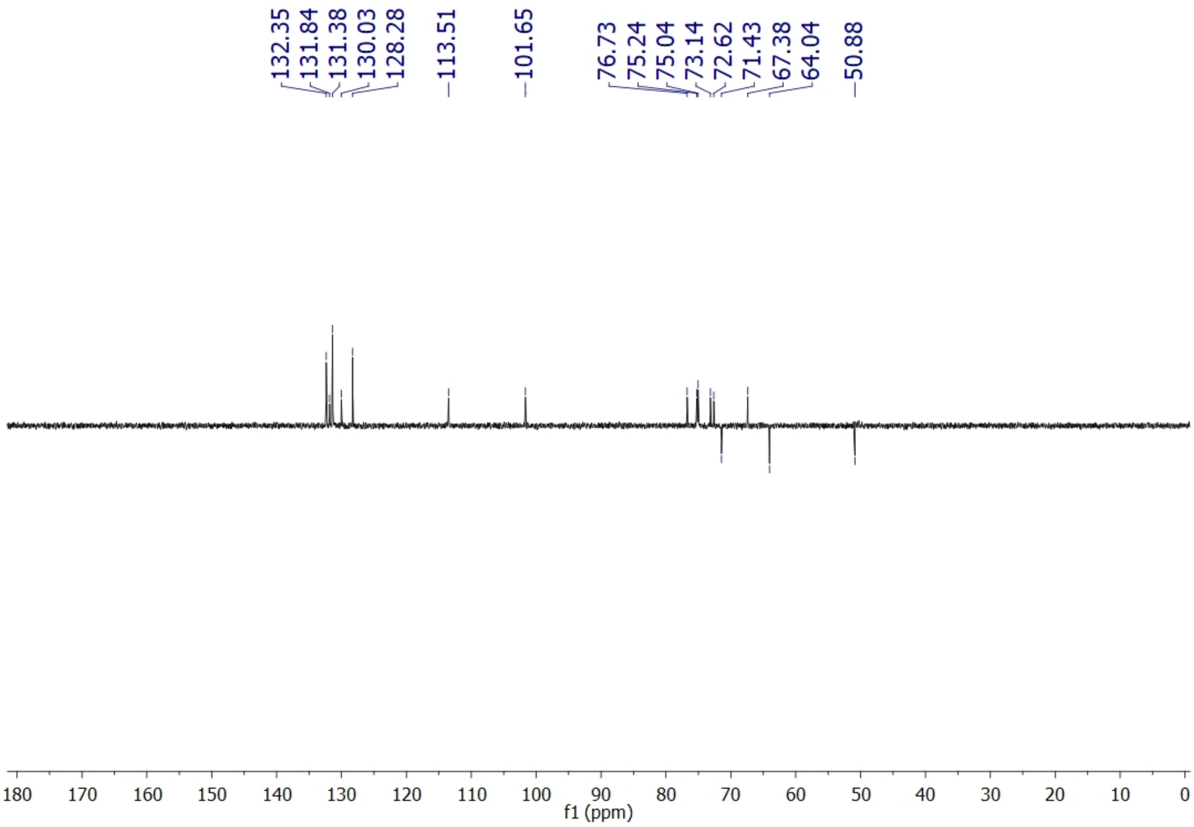

Figure S13. ^{1}H NMR and ^{13}C NMR spectra of 1-Benzyl-2-phenyl-5-((1*R*,2*S*,3*R*)-1,2,3-trihydroxy-4-(((2*S*,3*R*,4*S*,5*S*,6*R*)-3,4,5-trihydroxy-6-(hydroxymethyl)tetrahydro-2*H*-pyran-2-yl)oxy)butyl)-1*H*-pyrrole-3-carbonitrile.

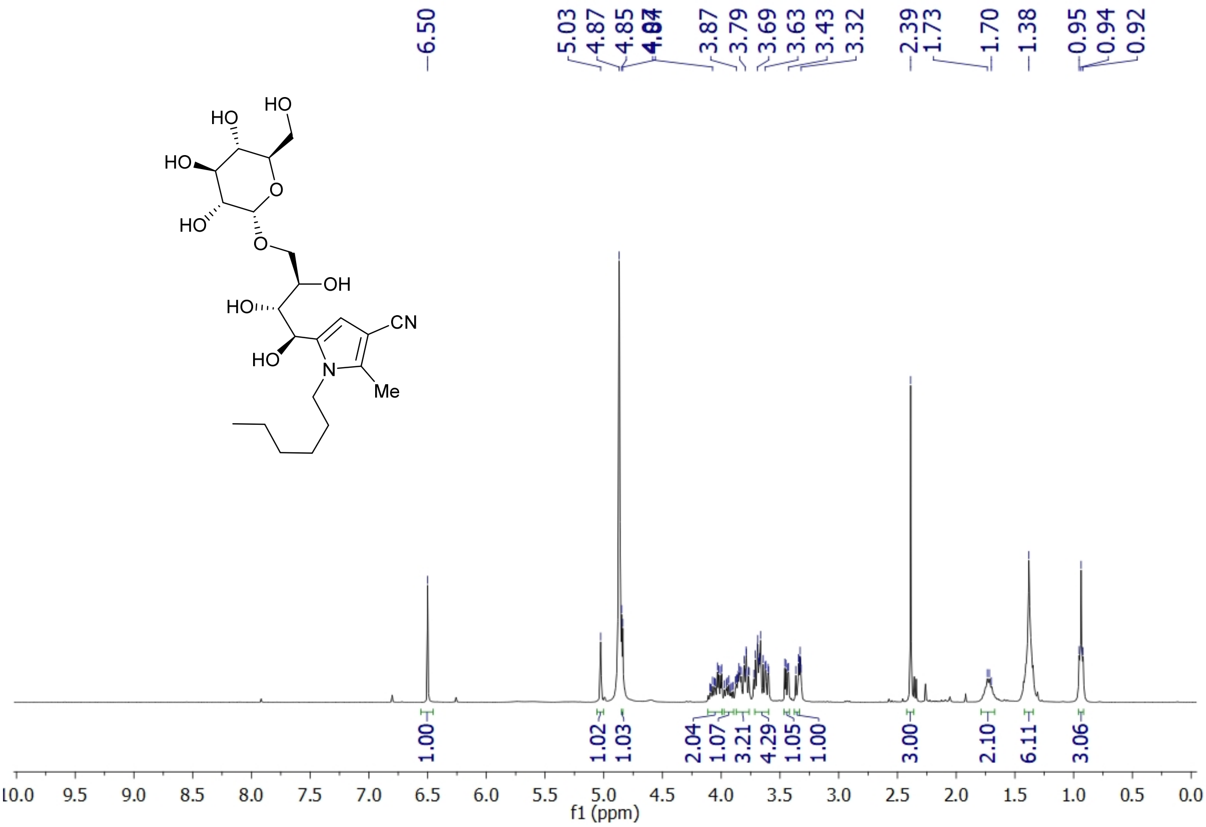
HO
HO
HO
HO
O
O
OH
HO
CN
HO
N
Me
6.50
5.03
4.87
4.85
3.87
3.79
3.69
3.63
3.43
3.32
2.39
1.73
1.70
1.38
0.95
0.94
0.92
1.00
1.02
1.03
2.04
1.07
3.21
4.29
1.05
1.00
3.00
2.10
6.11
3.06
f1 (ppm)

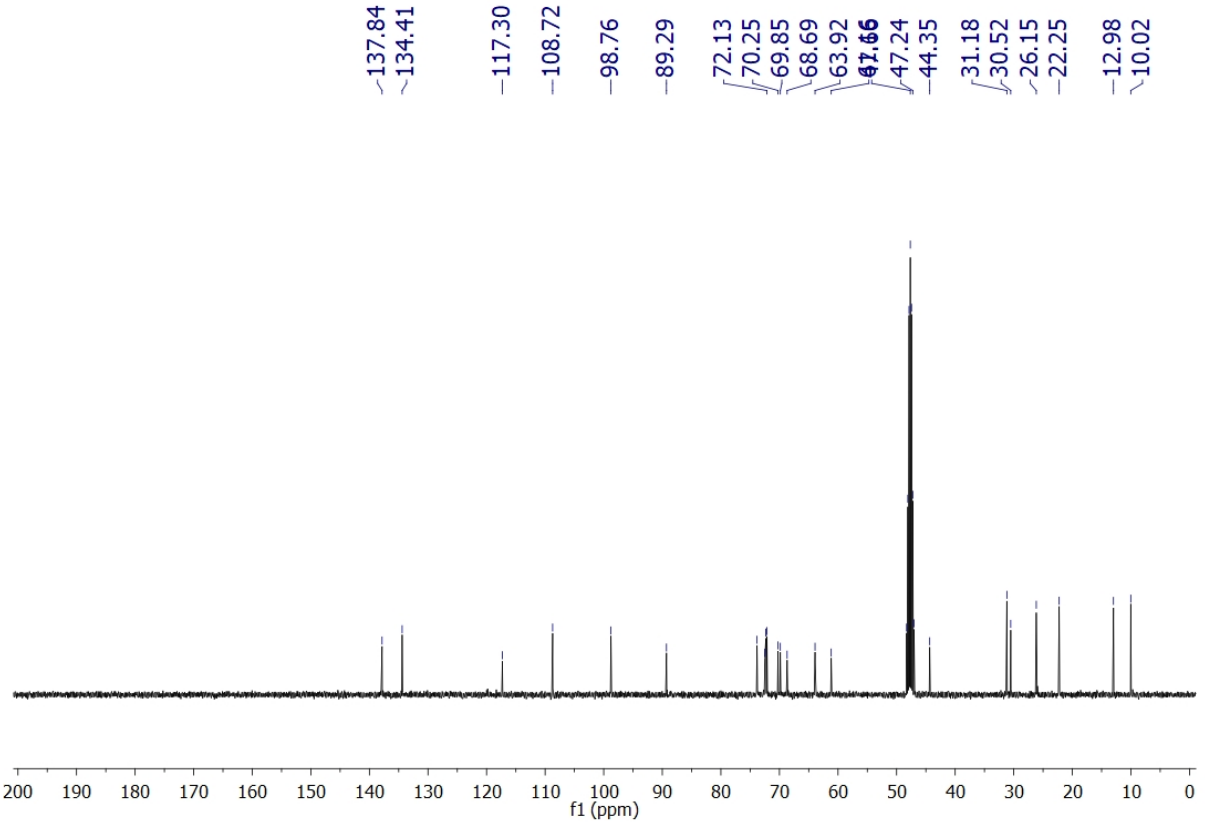
137.84
134.41
117.30
108.72
98.76
89.29
72.13
70.25
69.85
68.69
63.92
47.24
44.35
31.18
30.52
26.15
22.25
12.98
10.02
f1 (ppm)

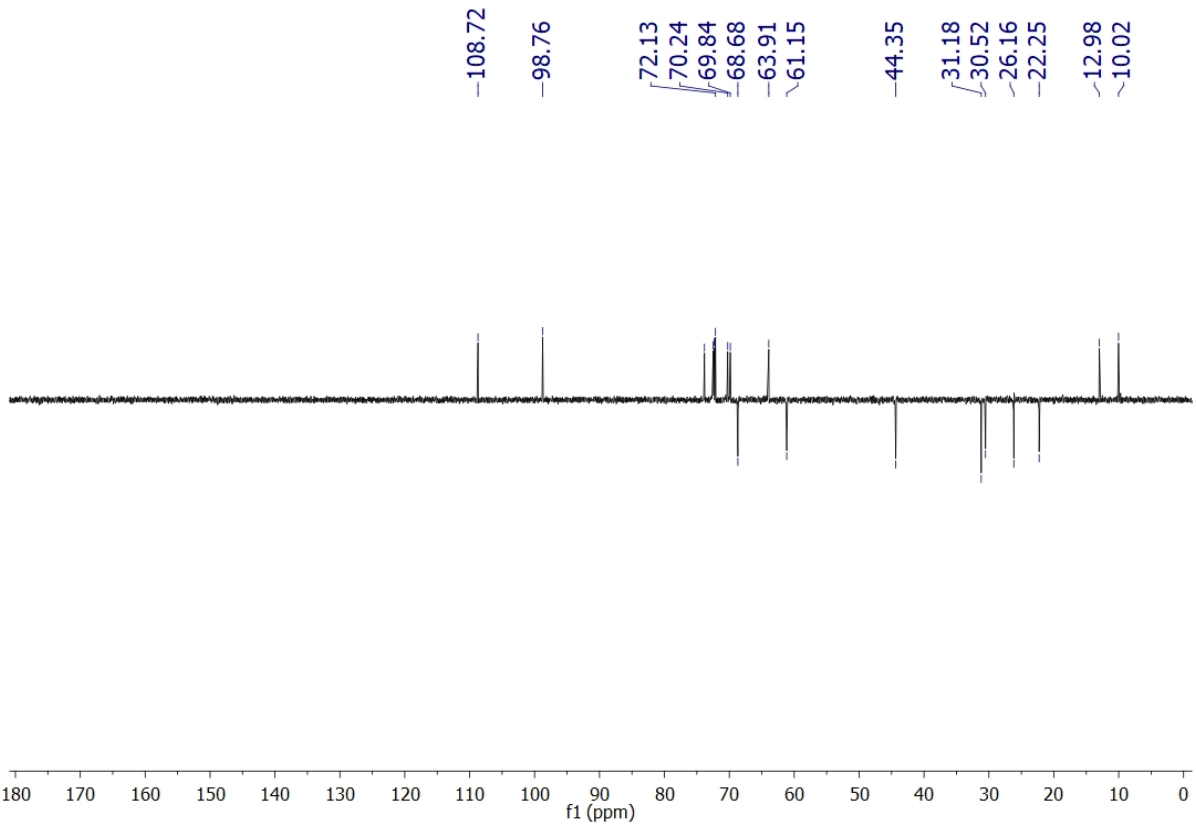

Figure S14. ^{1}H NMR and ^{13}C NMR spectra of 1-Hexyl-2-methyl-5-((1*R*,2*S*,3*R*)-1,2,3-trihydroxy-4-(((2*S*,3*R*,4*S*,5*S*,6*R*)-3,4,5-trihydroxy-6-(hydroxymethyl)tetrahydro-2H-pyran-2-yl)oxy)butyl)-1*H*-pyrrole-3-carbonitrile.

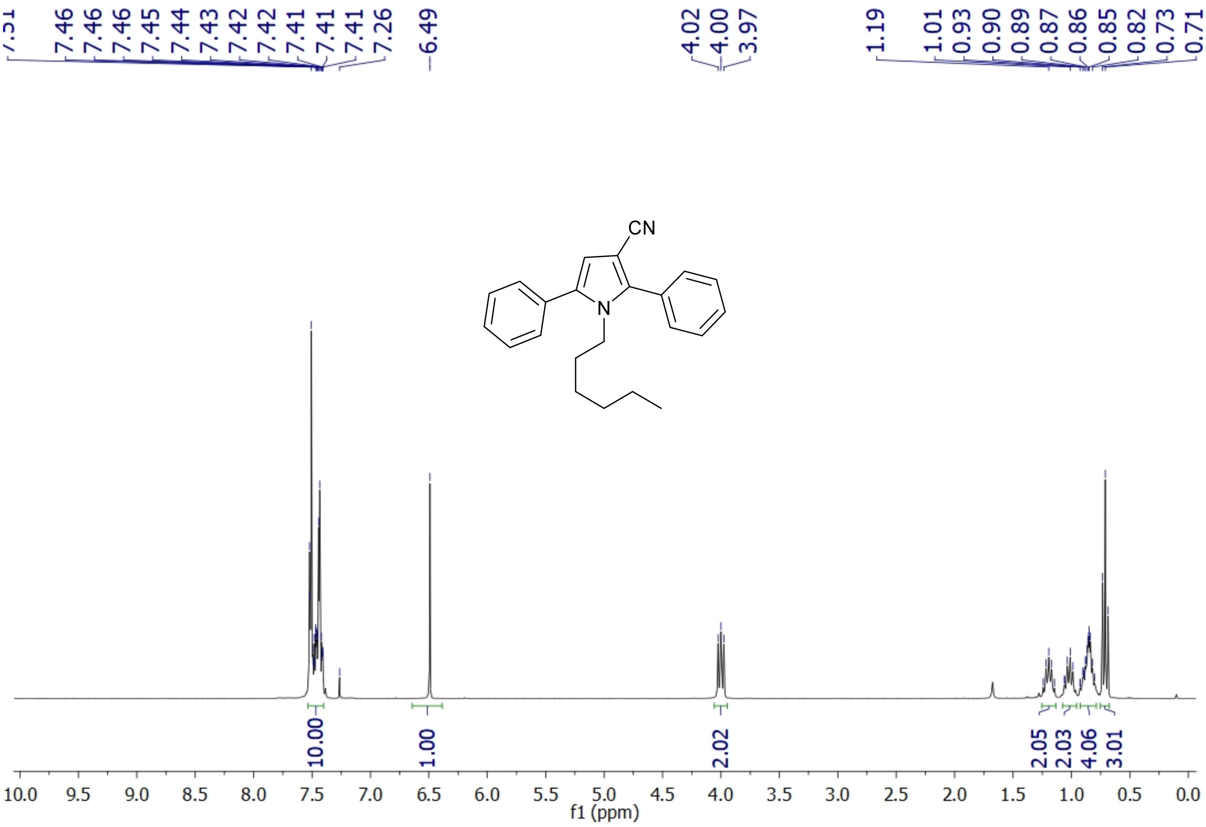
7.46
7.46
7.46
7.45
7.44
7.43
7.42
7.42
7.41
7.41
7.41
7.26
6.49
4.02
4.00
3.97
1.19
1.01
0.93
0.90
0.89
0.87
0.86
0.85
0.82
0.73
0.71
CN
N
10.00
1.00
2.02
2.05
2.03
4.06
3.01
10.0 9.5 9.0 8.5 8.0 7.5 7.0 6.5 6.0 5.5 5.0 4.5 4.0 3.5 3.0 2.5 2.0 1.5 1.0 0.5 0.0
f1 (ppm)

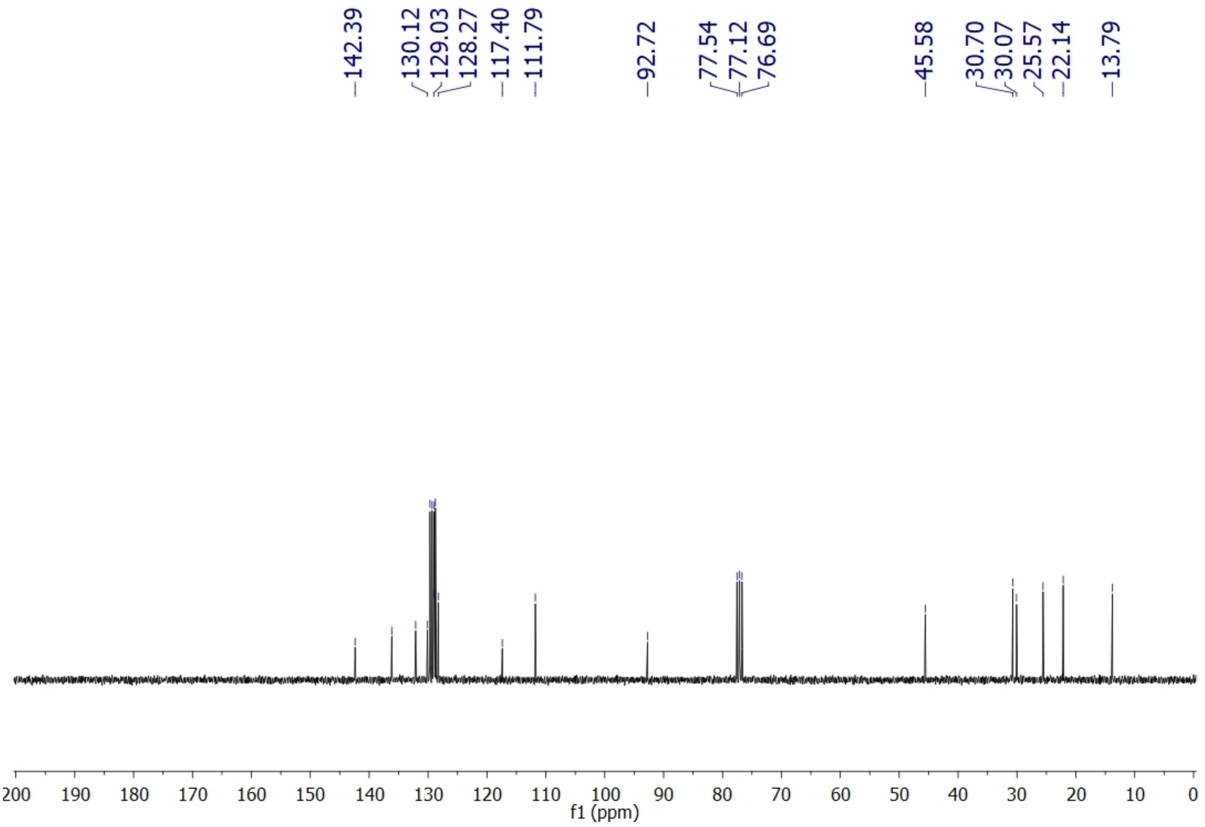
142.39
130.12
129.03
128.27
117.40
111.79
92.72
77.54
77.12
76.69
45.58
30.70
30.07
25.57
22.14
13.79
200 190 180 170 160 150 140 130 120 110 100 90 80 70 60 50 40 30 20 10 0
f1 (ppm)

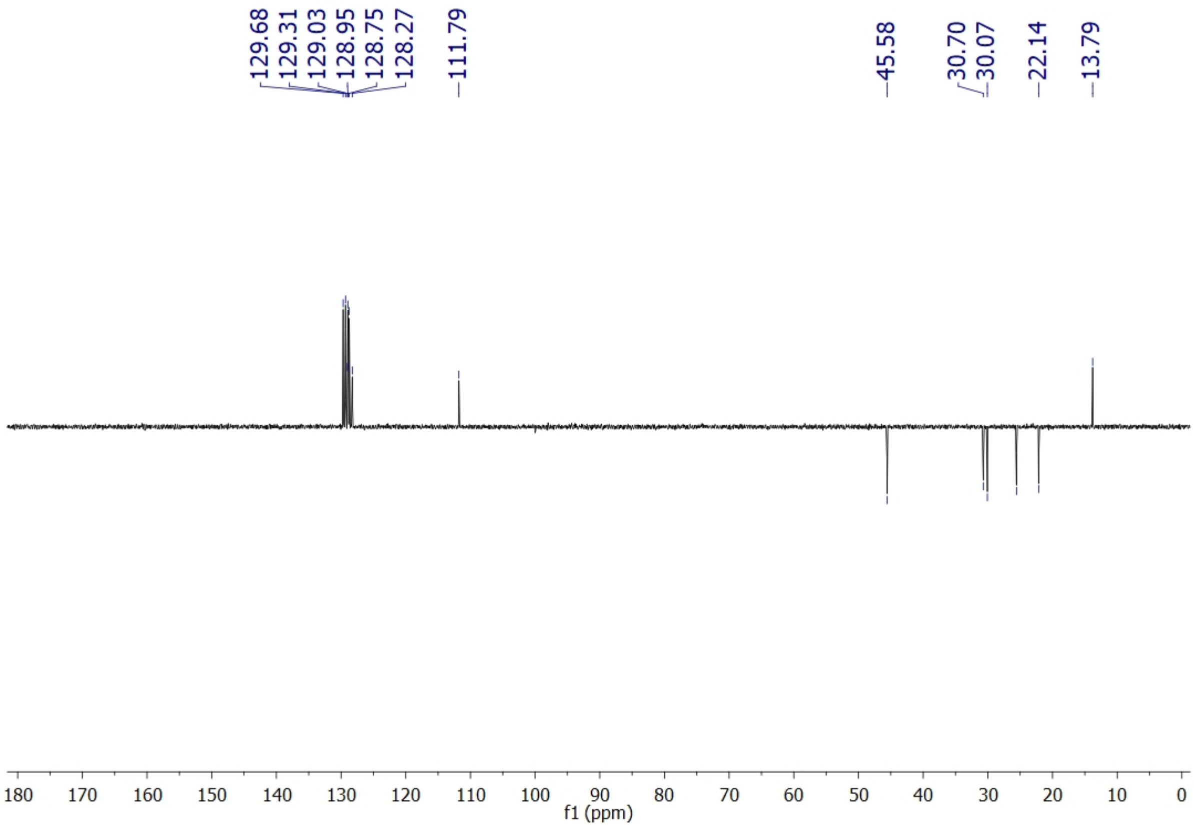

Figure S15. ^{1}H NMR and ^{13}C NMR spectra of 1-Hexyl-2,5-diphenyl-1*H*-pyrrole-3-carbonitrile.

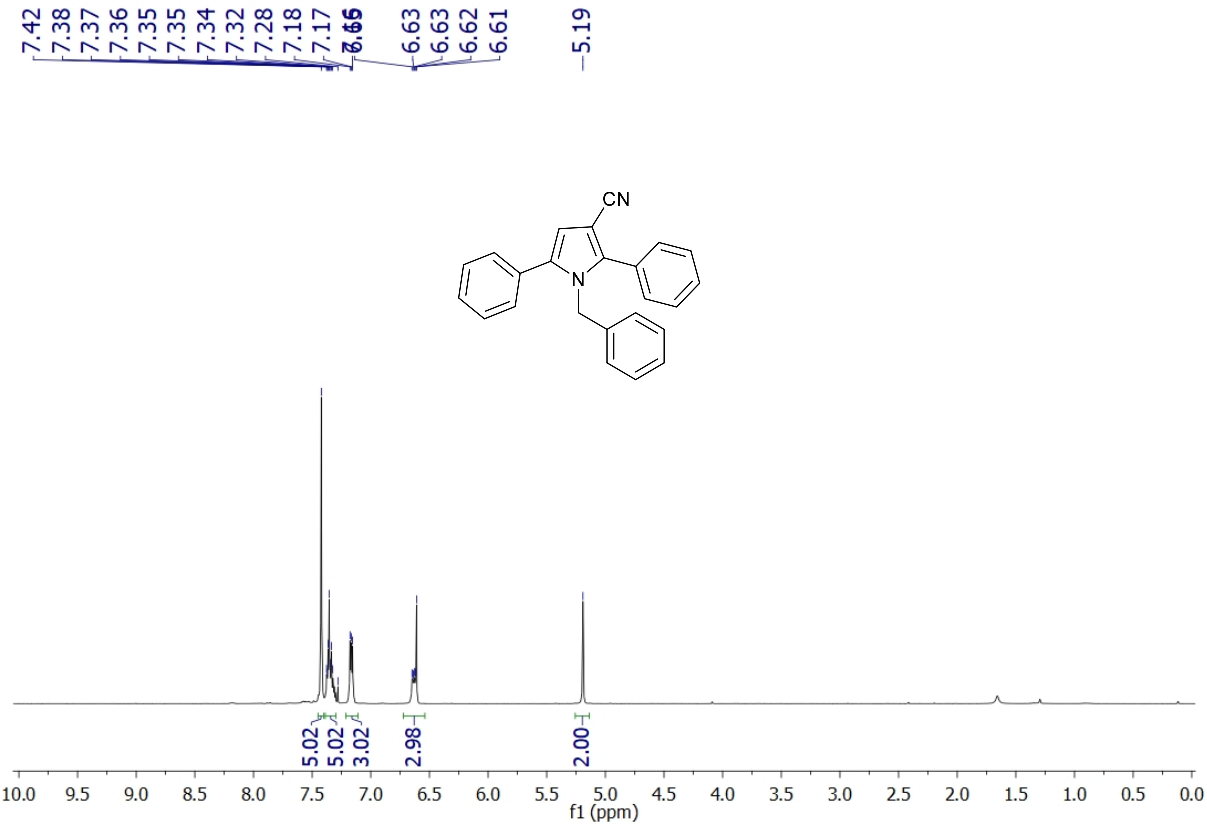
7.42
7.38
7.37
7.36
7.35
7.35
7.34
7.32
7.28
7.18
7.17
6.63
6.63
6.62
6.61
5.19
CN
N
5.02
5.02
3.02
2.98
2.00
10.0 9.5 9.0 8.5 8.0 7.5 7.0 6.5 6.0 5.5 5.0 4.5 4.0 3.5 3.0 2.5 2.0 1.5 1.0 0.5 0.0
f1 (ppm)

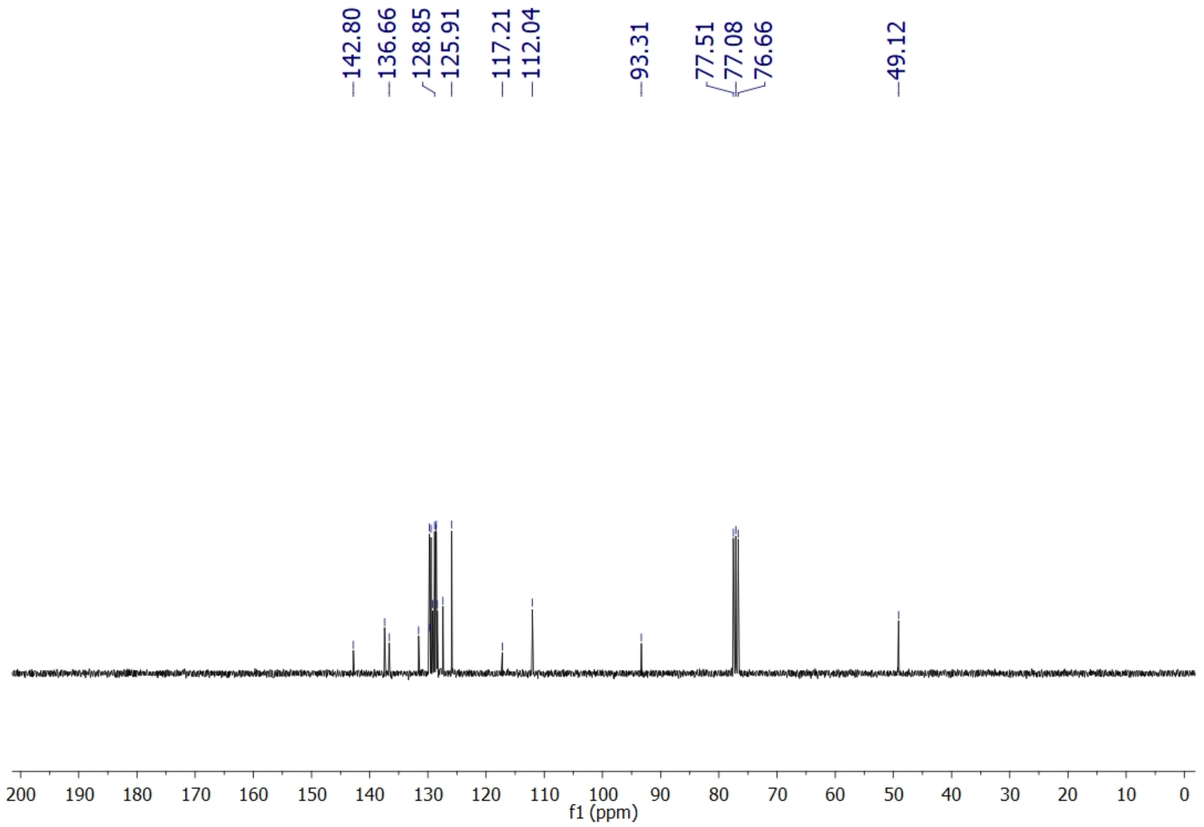
142.80
136.66
128.85
125.91
117.21
112.04
93.31
77.51
77.08
76.66
49.12
200 190 180 170 160 150 140 130 120 110 100 90 80 70 60 50 40 30 20 10 0
f1 (ppm)

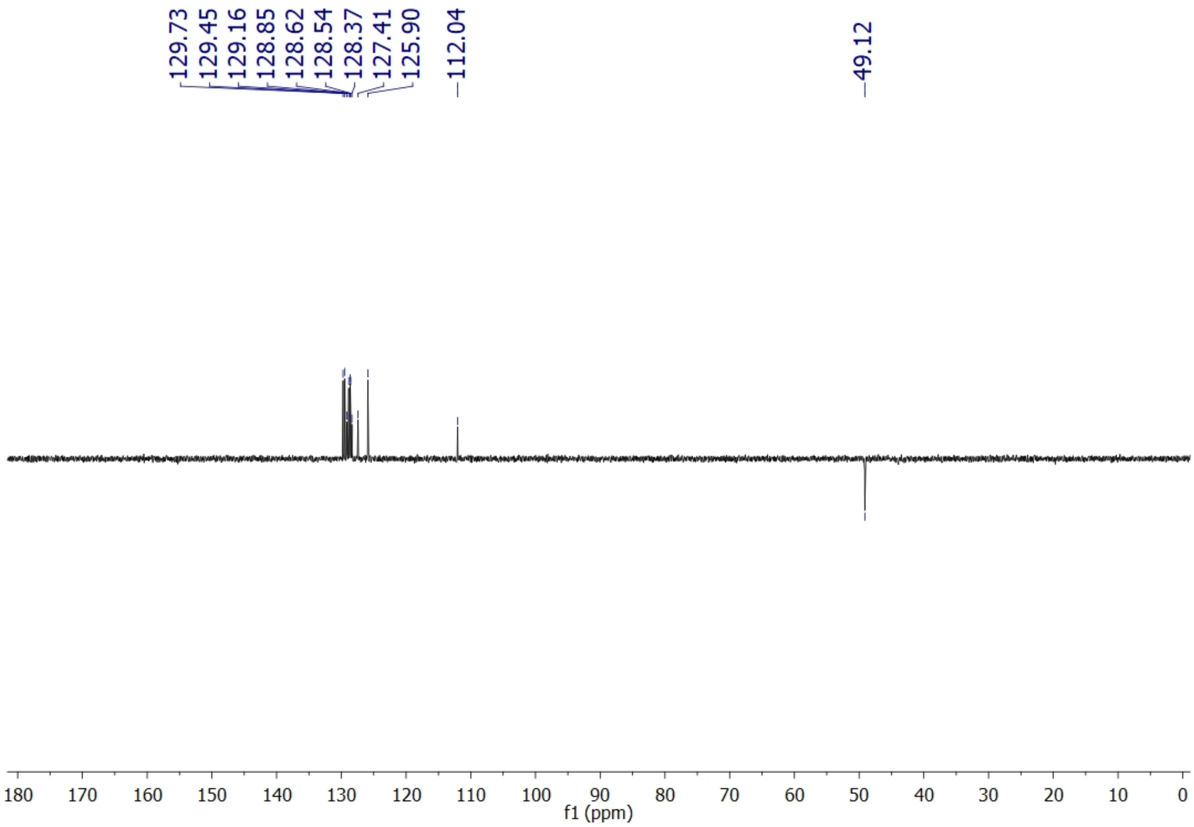

Figure S16. ^{1}H NMR and ^{13}C NMR spectra of 1-Benzyl-2,5-diphenyl-1*H*-pyrrole-3-carbonitrile.

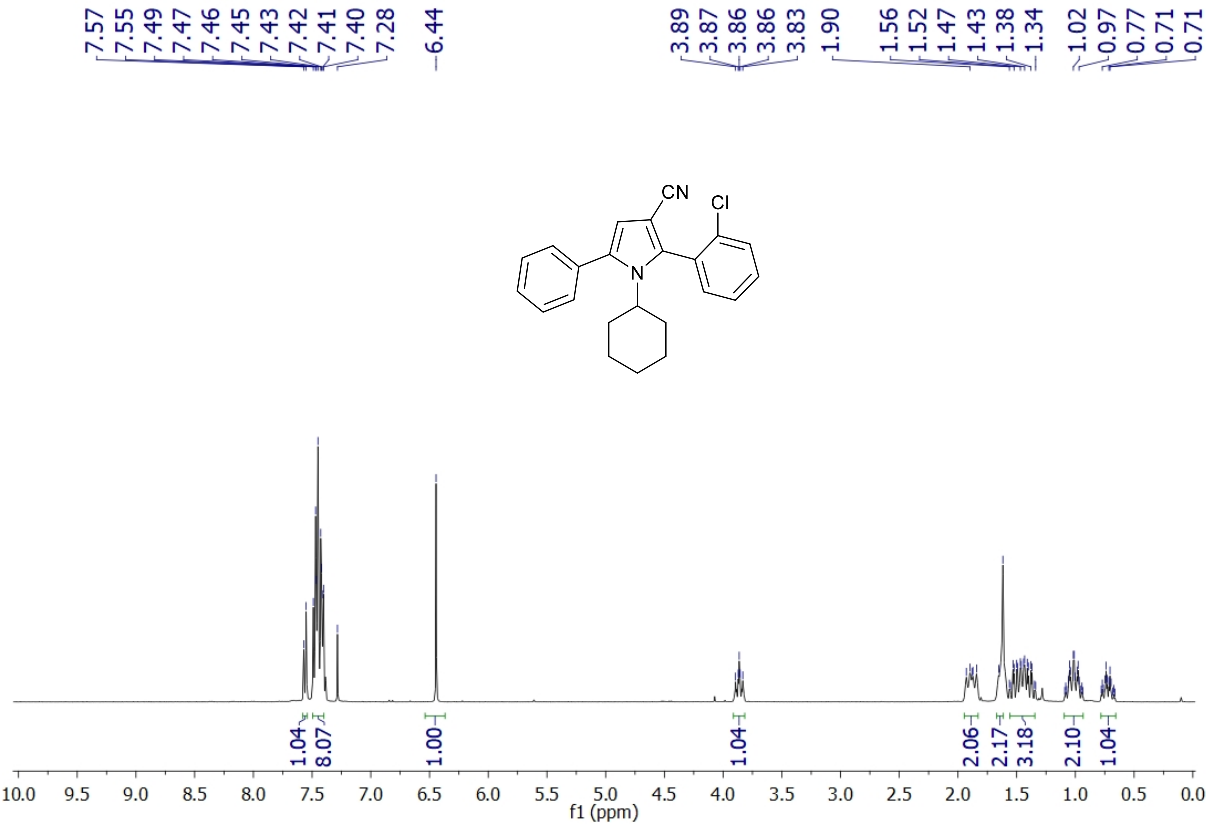
7.57 7.55 7.49 7.47 7.46 7.45 7.43 7.42 7.41 7.40 7.28
6.44
3.89 3.87 3.86 3.86 3.83
1.90
1.56 1.52 1.47 1.43 1.38 1.34
1.02 0.97 0.77 0.71 0.71
CN
Cl
N
1.04 8.07 1.00 1.04 2.06 2.17 3.18 2.10 1.04
10.0 9.5 9.0 8.5 8.0 7.5 7.0 6.5 6.0 5.5 5.0 4.5 4.0 3.5 3.0 2.5 2.0 1.5 1.0 0.5 0.0
f1 (ppm)

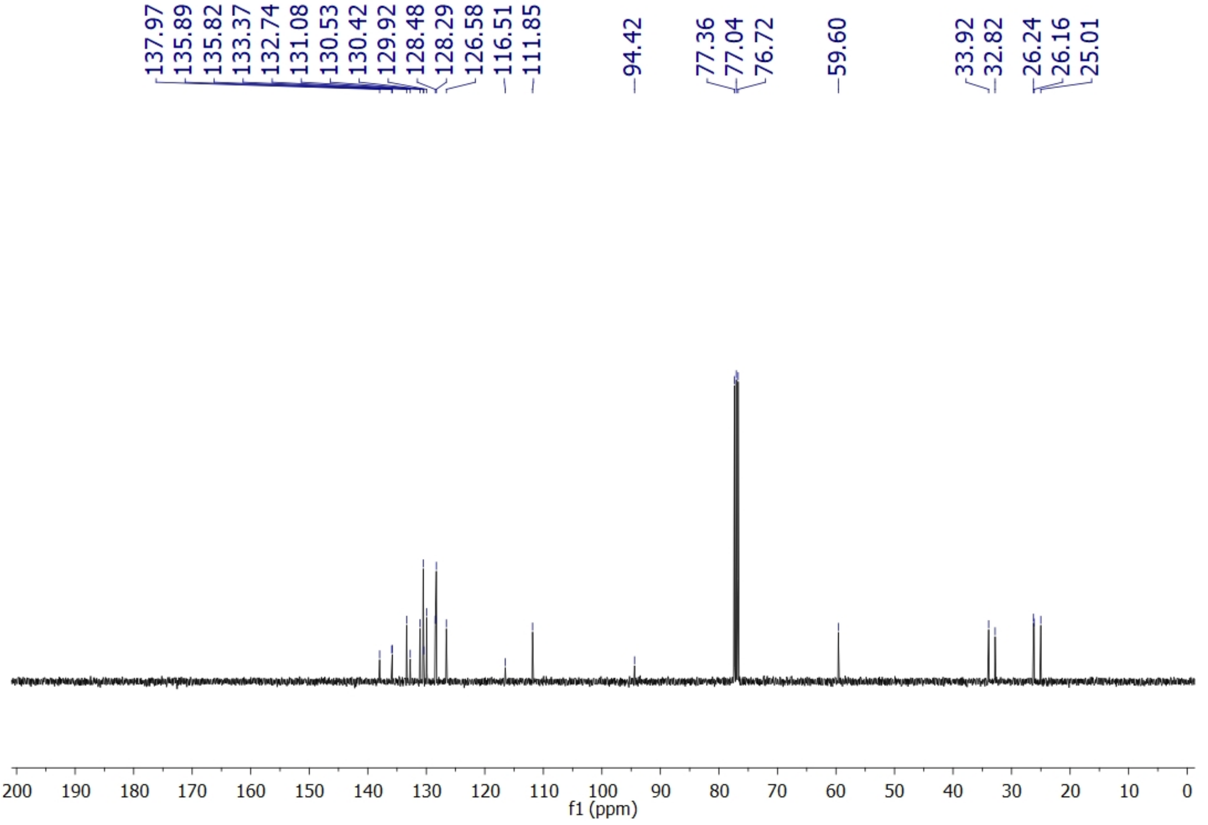
137.97 135.89 135.82 133.37 132.74 131.08 130.53 130.42 129.92 128.48 128.29 126.58 116.51 111.85
94.42
77.36 77.04 76.72
59.60
33.92 32.82 26.24 26.16 25.01
200 190 180 170 160 150 140 130 120 110 100 90 80 70 60 50 40 30 20 10 0
f1 (ppm)

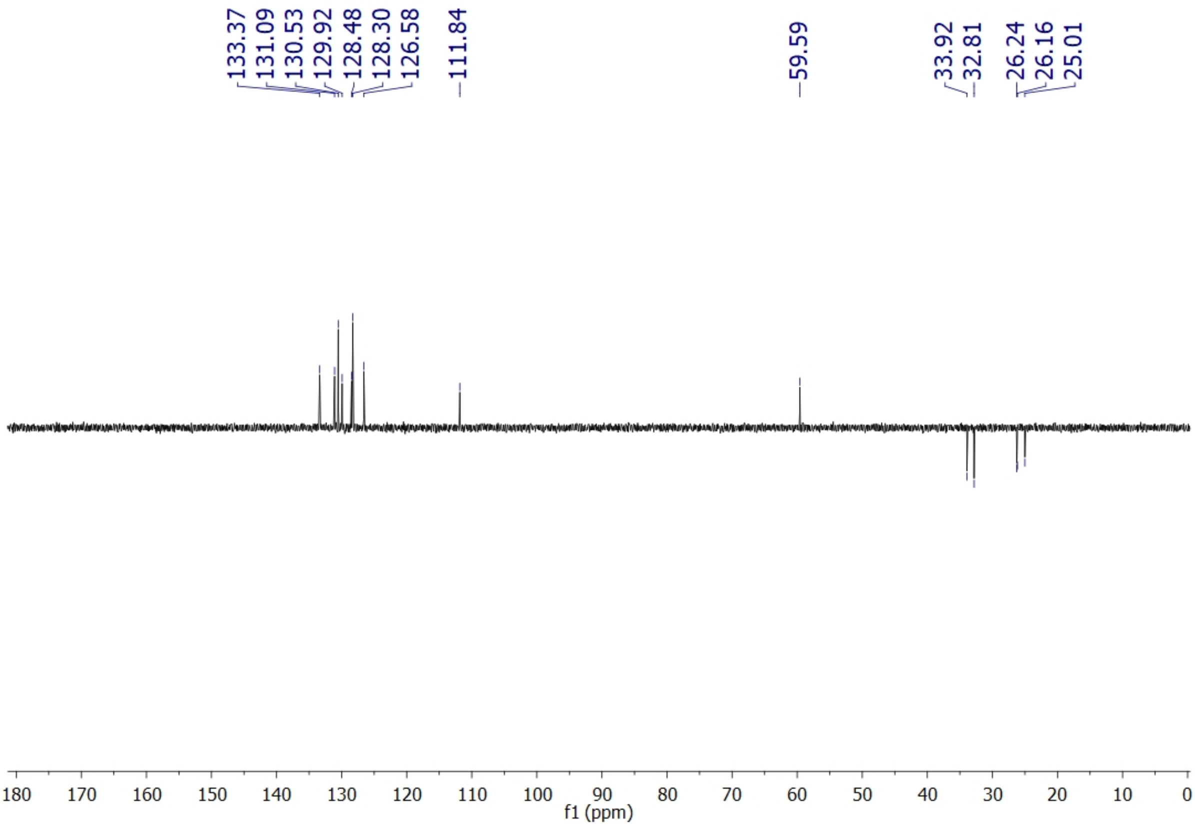

Figure S17. ^{1}H NMR and ^{13}C NMR spectra of 2-(2-Chlorophenyl)-1-cyclohexyl-5-phenyl-1*H*-pyrrole-3-carbonitrile.

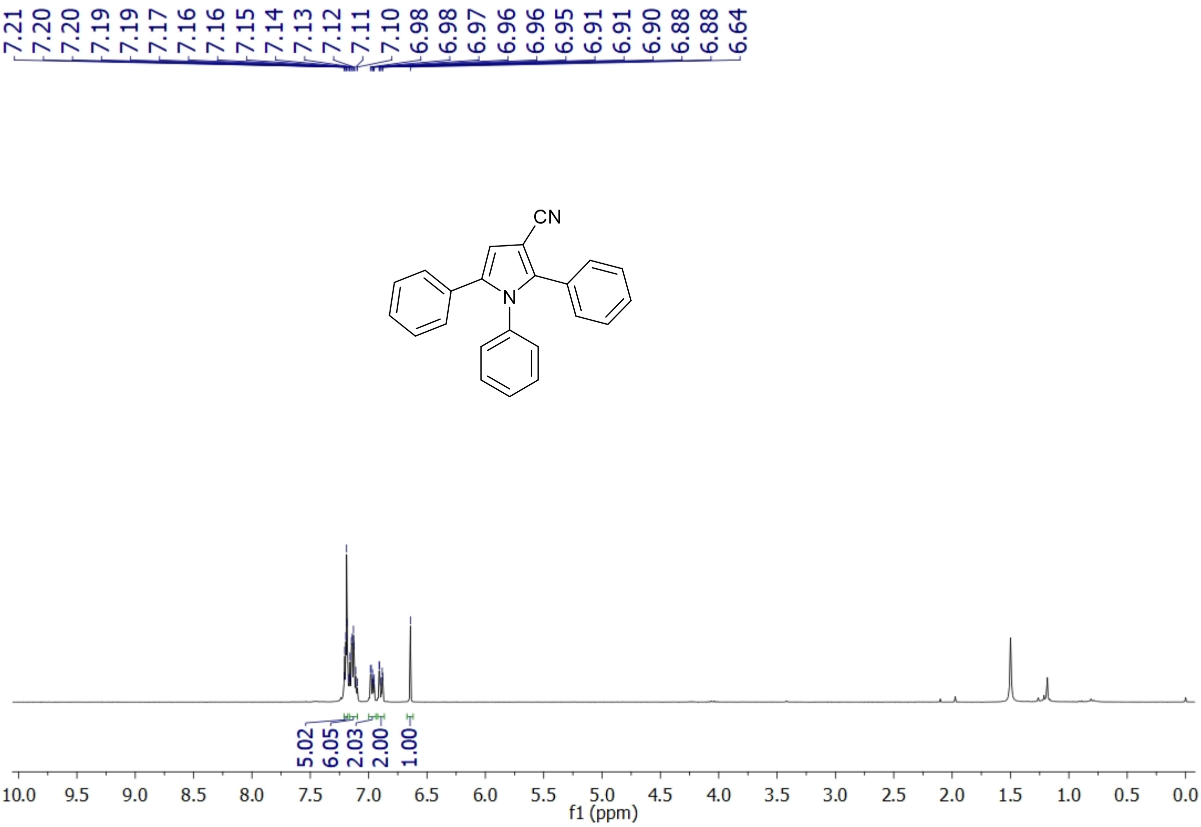
7.21 7.20 7.20 7.19 7.19 7.17 7.16 7.16 7.15 7.14 7.13 7.12 7.11 7.10 6.98 6.98 6.97 6.96 6.96 6.95 6.91 6.91 6.90 6.88 6.88 6.64
CN
N
5.02 6.05 2.03 2.00 1.00
10.0 9.5 9.0 8.5 8.0 7.5 7.0 6.5 6.0 5.5 5.0 4.5 4.0 3.5 3.0 2.5 2.0 1.5 1.0 0.5 0.0
f1 (ppm)

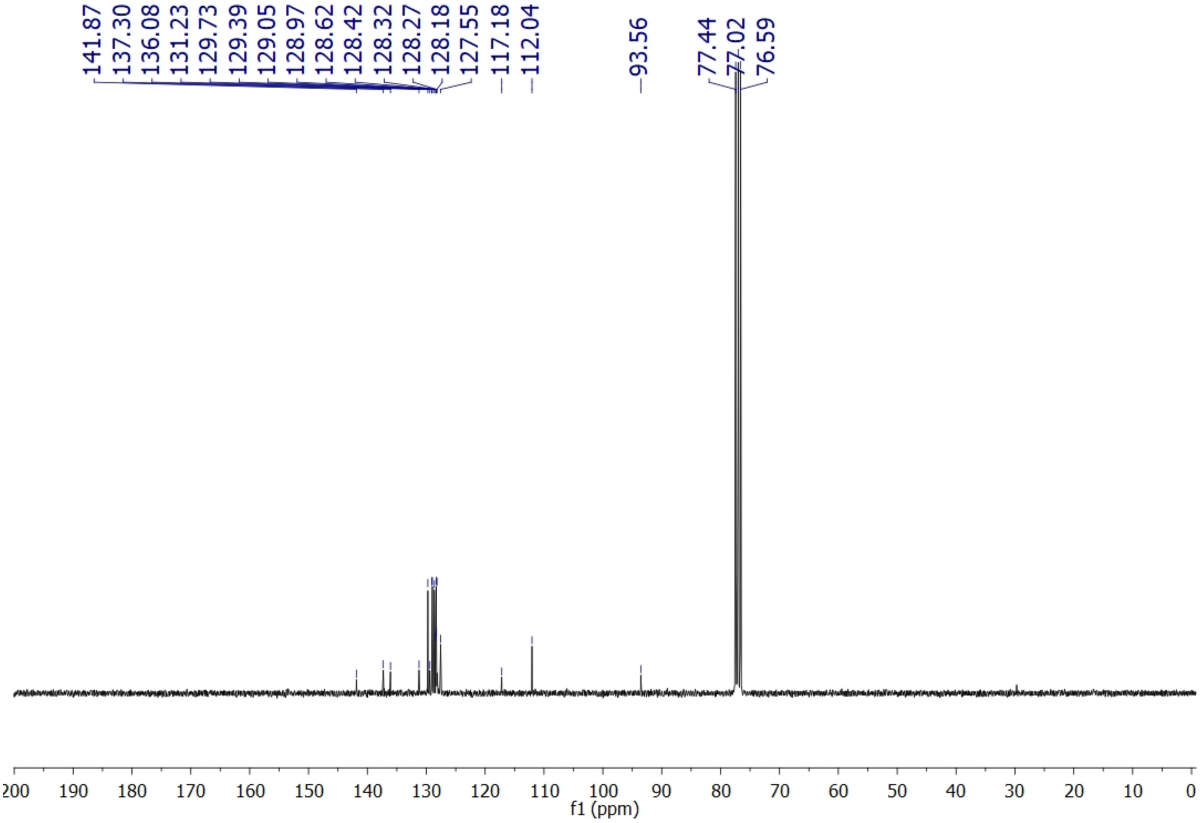
141.87 137.30 136.08 131.23 129.73 129.39 129.05 128.97 128.62 128.42 128.32 128.27 128.18 127.55 117.18 112.04
93.56
77.44 77.02 76.59
200 190 180 170 160 150 140 130 120 110 100 90 80 70 60 50 40 30 20 10 0
f1 (ppm)

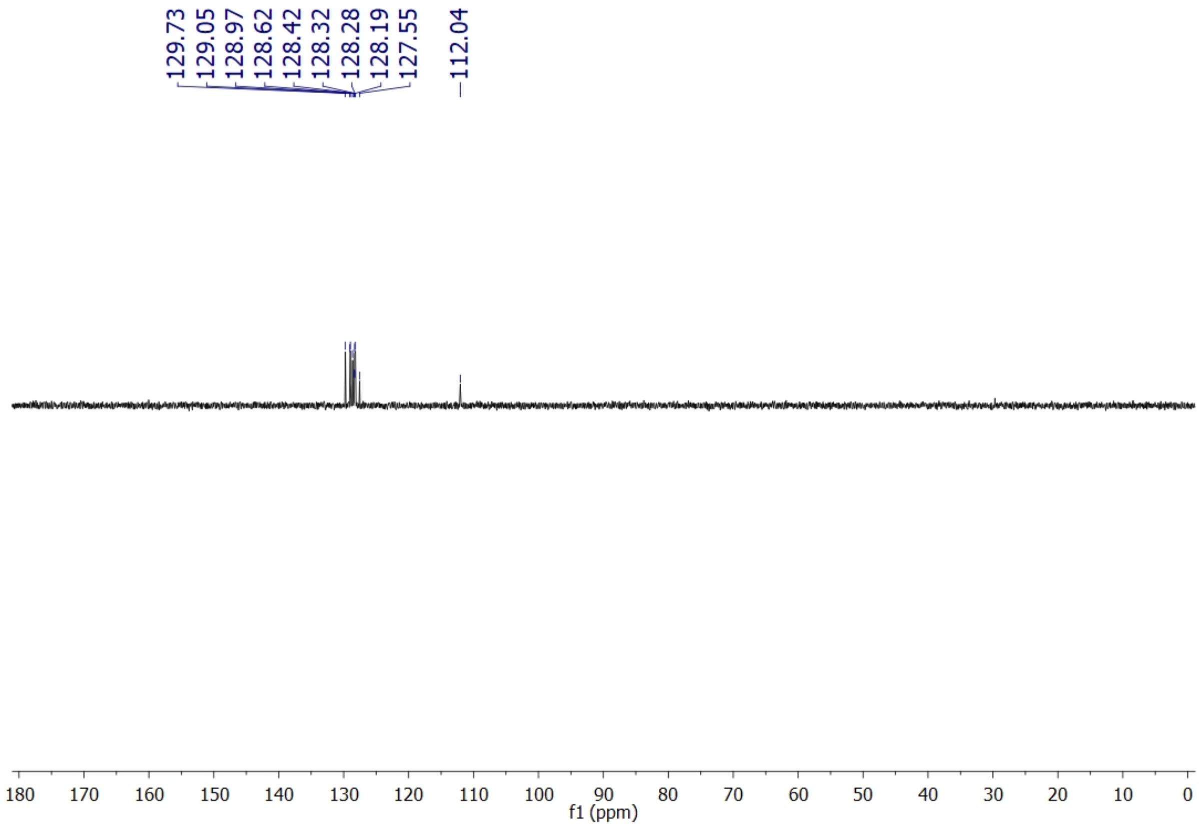

Figure S18. ^{1}H NMR and ^{13}C NMR spectra of 1,2,5-Triphenyl-1*H*-pyrrole-3-carbonitrile.

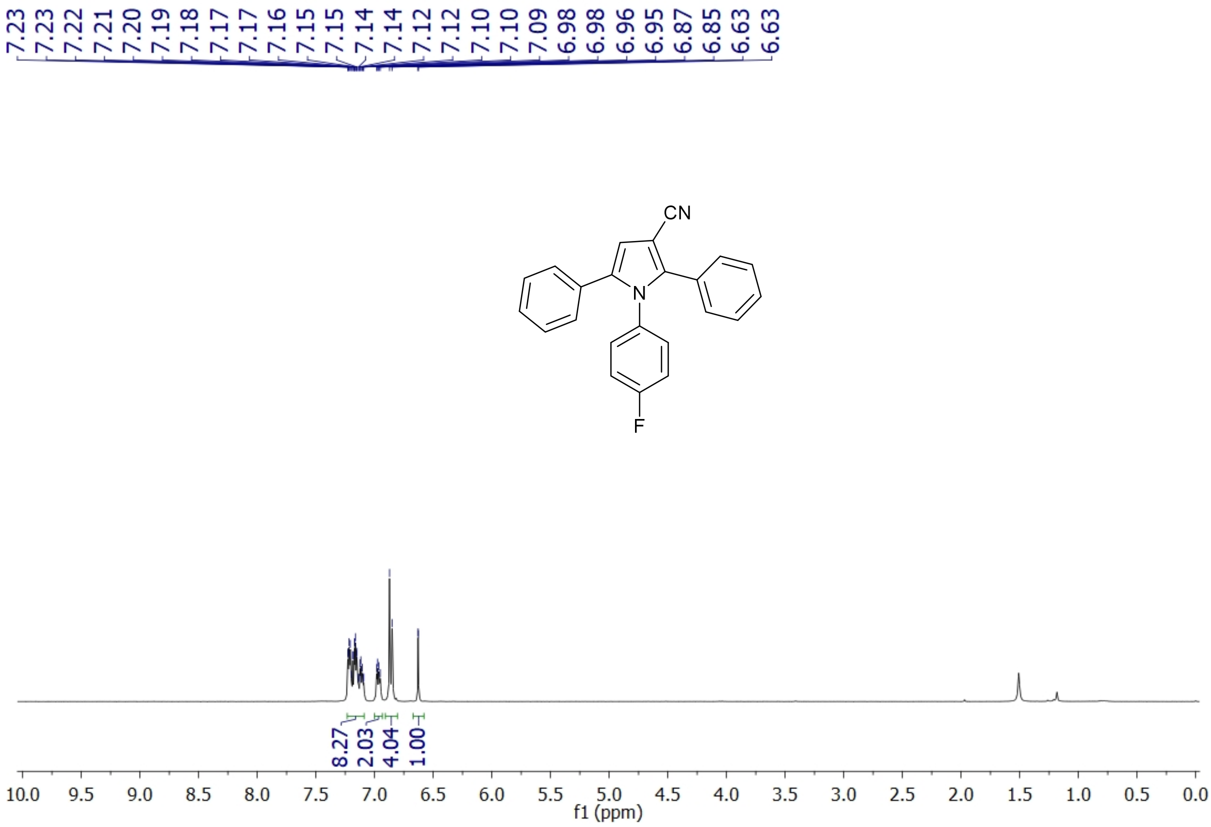
7.23 7.23 7.22 7.21 7.20 7.19 7.18 7.17 7.17 7.16 7.15 7.15 7.15 7.14 7.14 7.12 7.12 7.10 7.10 7.09 6.98 6.98 6.96 6.95 6.87 6.85 6.63 6.63
CN
N
F
8.27 2.03 4.04 1.00
10.0 9.5 9.0 8.5 8.0 7.5 7.0 6.5 6.0 5.5 5.0 4.5 4.0 3.5 3.0 2.5 2.0 1.5 1.0 0.5 0.0
f1 (ppm)

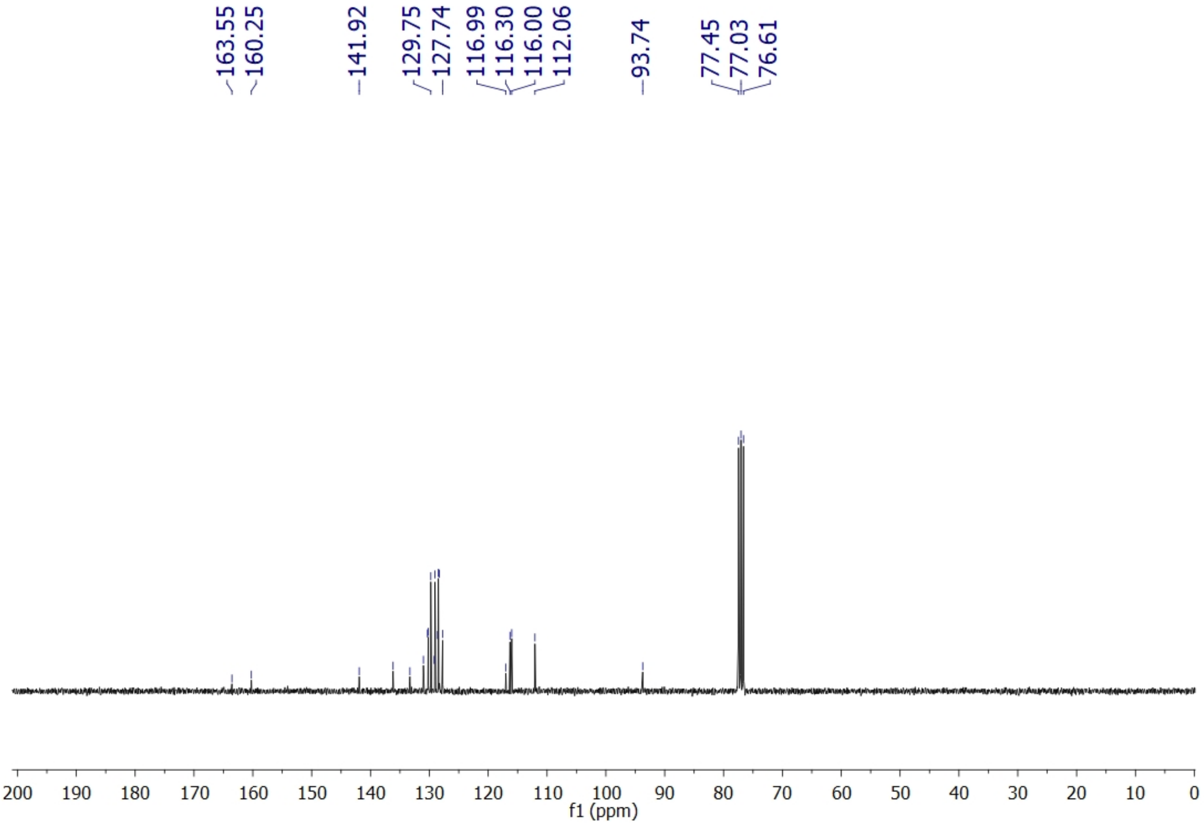
163.55 160.25 141.92 129.75 127.74 116.99 116.30 116.00 112.06 93.74 77.45 77.03 76.61
200 190 180 170 160 150 140 130 120 110 100 90 80 70 60 50 40 30 20 10 0
f1 (ppm)

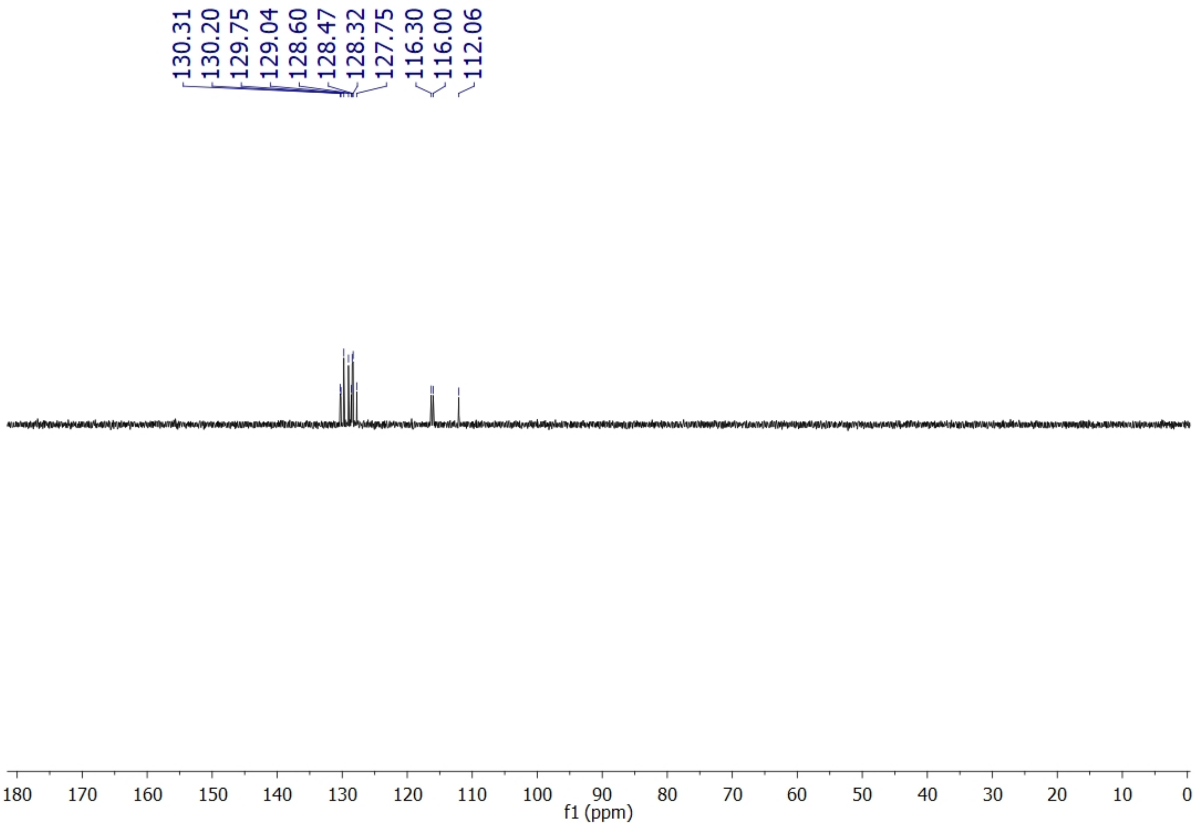

Figure S19. ^{1}H NMR and ^{13}C NMR spectra of 1-(4-Fluorophenyl)-2,5-diphenyl-1*H*-pyrrole-3-carbonitrile.

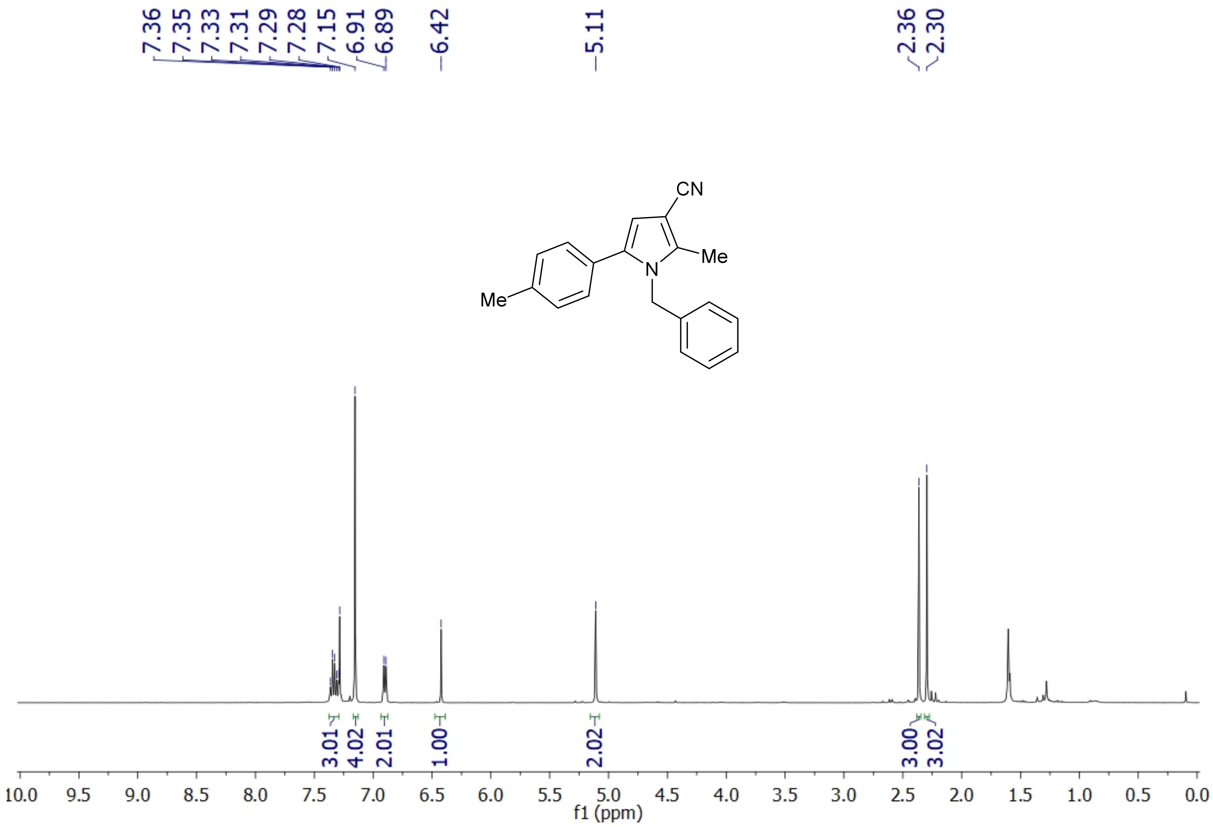
7.36
7.35
7.33
7.31
7.29
7.28
7.15
6.91
6.89
6.42
5.11
2.36
2.30
CN
Me
N
Me
3.01
4.02
2.01
1.00
2.02
3.00
3.02
f1 (ppm)

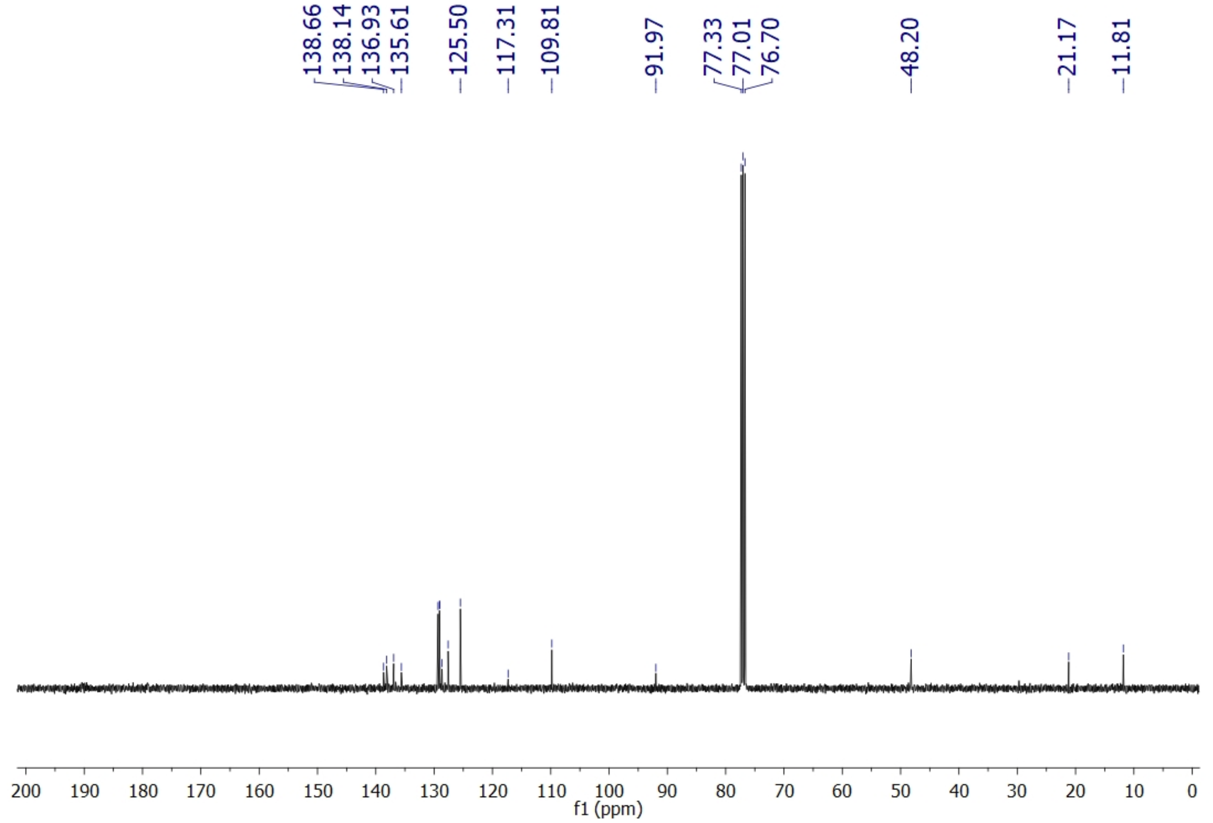
138.66
138.14
136.93
135.61
125.50
117.31
109.81
91.97
77.33
77.01
76.70
48.20
21.17
11.81
f1 (ppm)

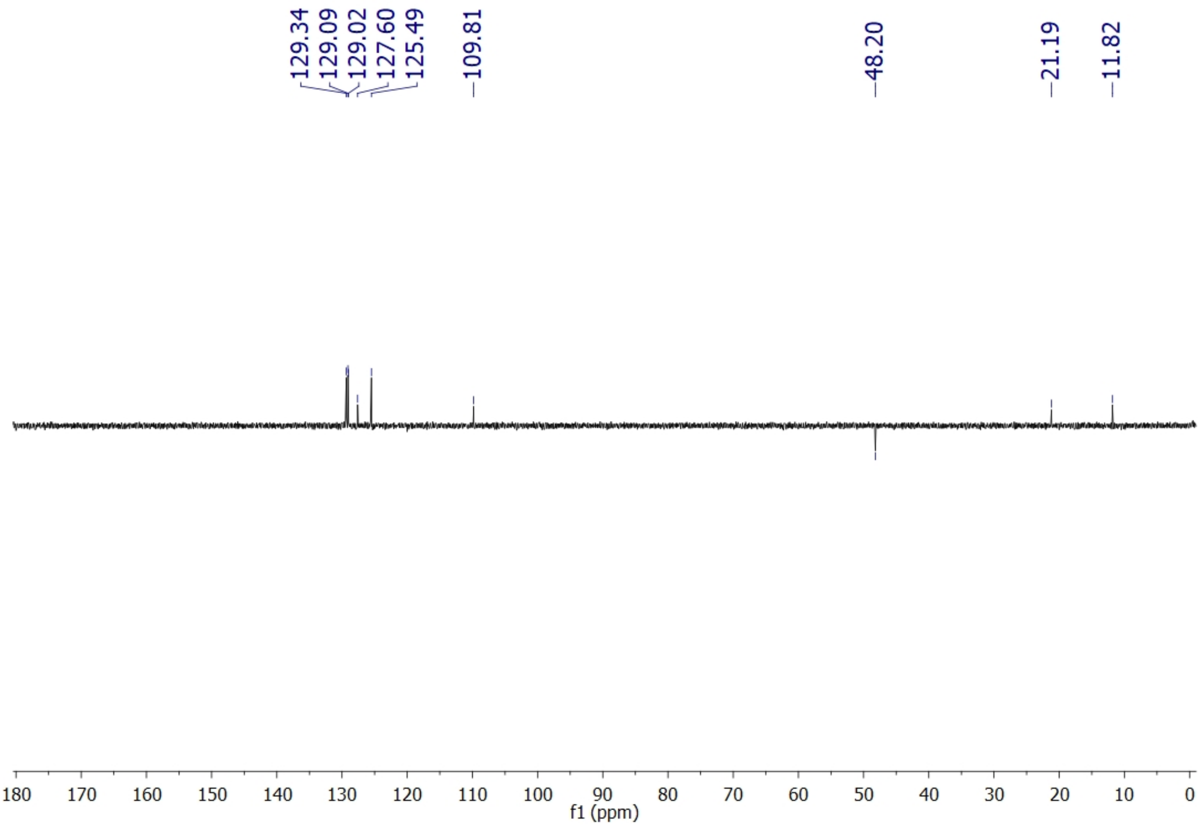

Figure S20. ^{1}H NMR and ^{13}C NMR spectra of 1-Benzyl-2-methyl-5-(p-tolyl)-1*H*-pyrrole-3-carbonitrile.

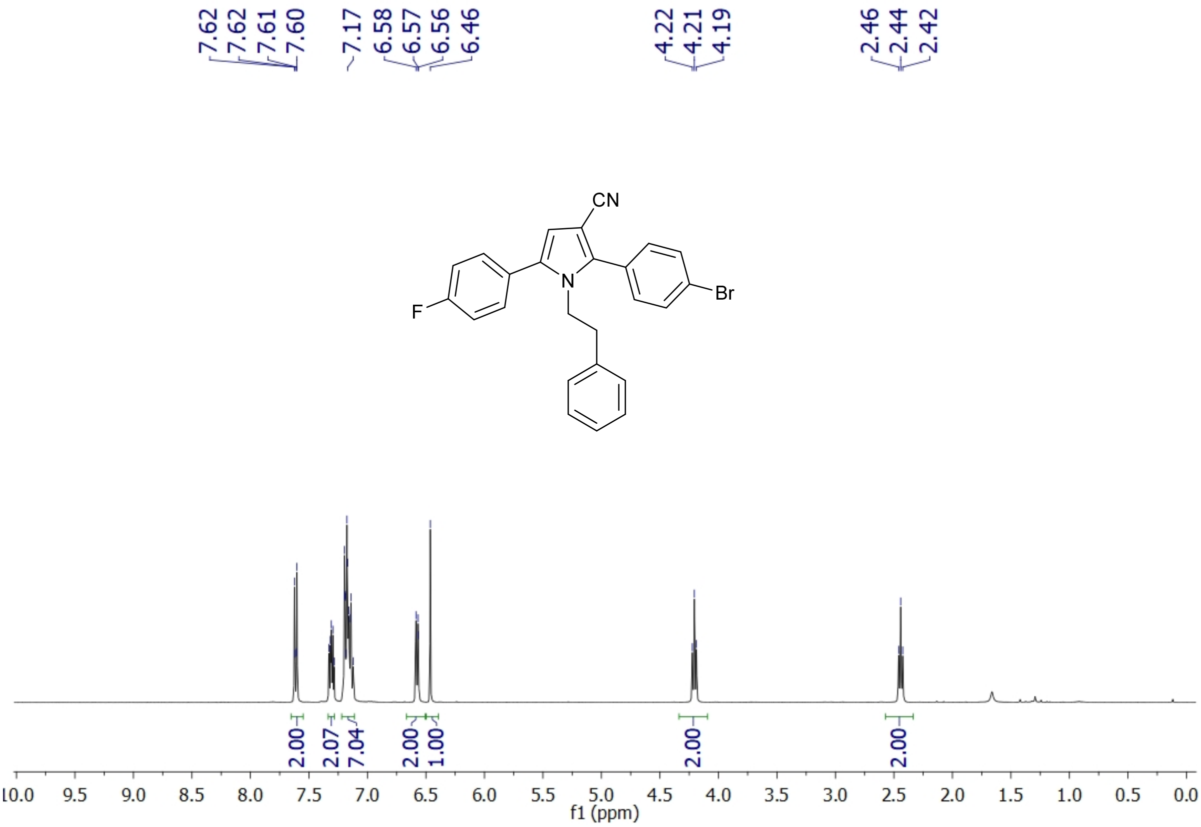
7.62
7.62
7.61
7.60
7.17
6.58
6.57
6.56
6.46
4.22
4.21
4.19
2.46
2.44
2.42
CN
F
N
Br
2.00
2.07
7.04
2.00
1.00
2.00
2.00
f1 (ppm)

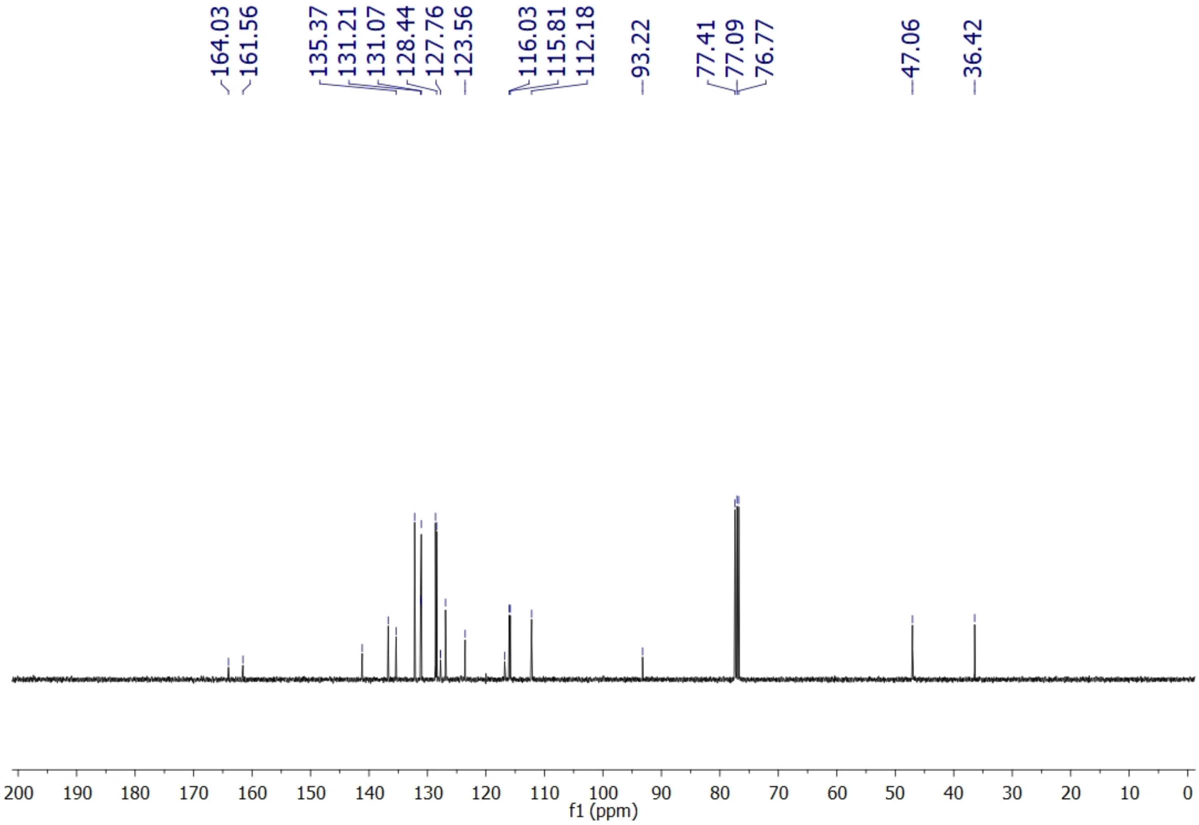
164.03
161.56
135.37
131.21
131.07
128.44
127.76
123.56
116.03
115.81
112.18
93.22
77.41
77.09
76.77
47.06
36.42
f1 (ppm)

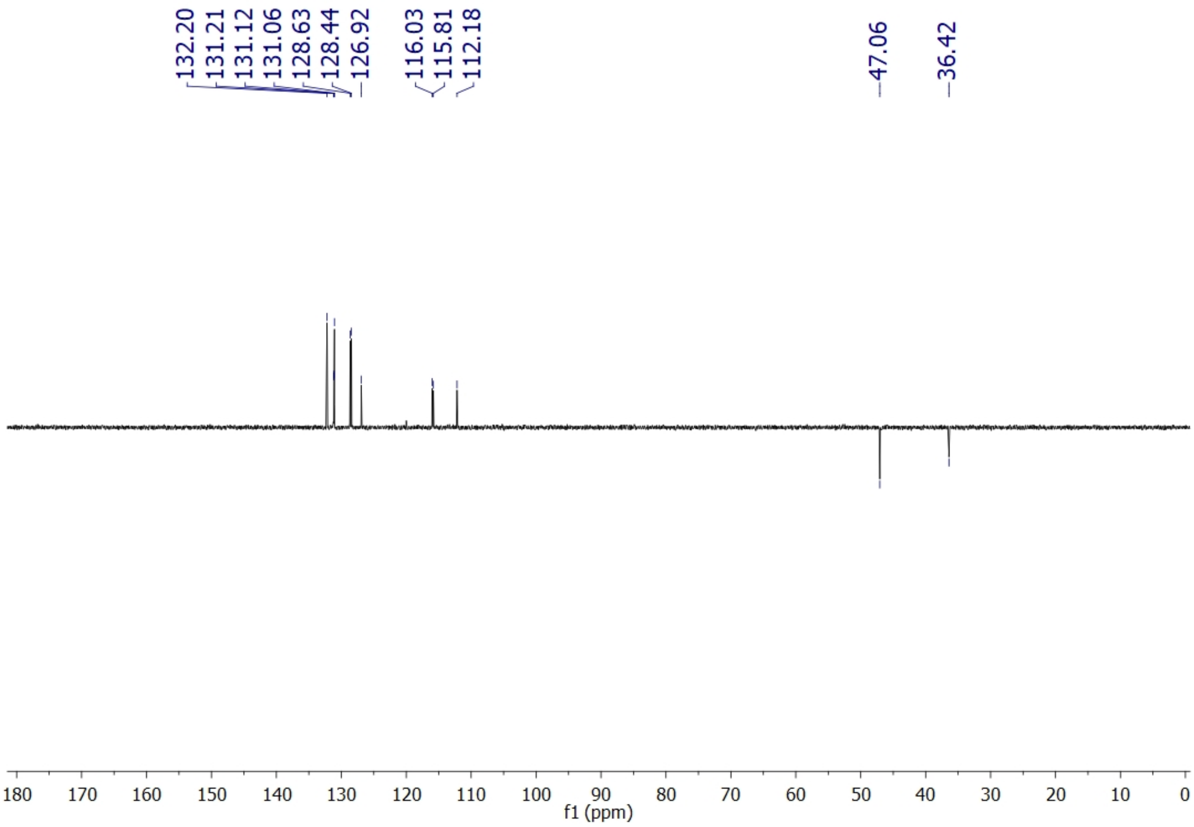

Figure S21. ^{1}H NMR and ^{13}C NMR spectra of 2-(4-Bromophenyl)-5-(4-fluorophenyl)-1-phenethyl-1*H*-pyrrole-3-carbonitrile.

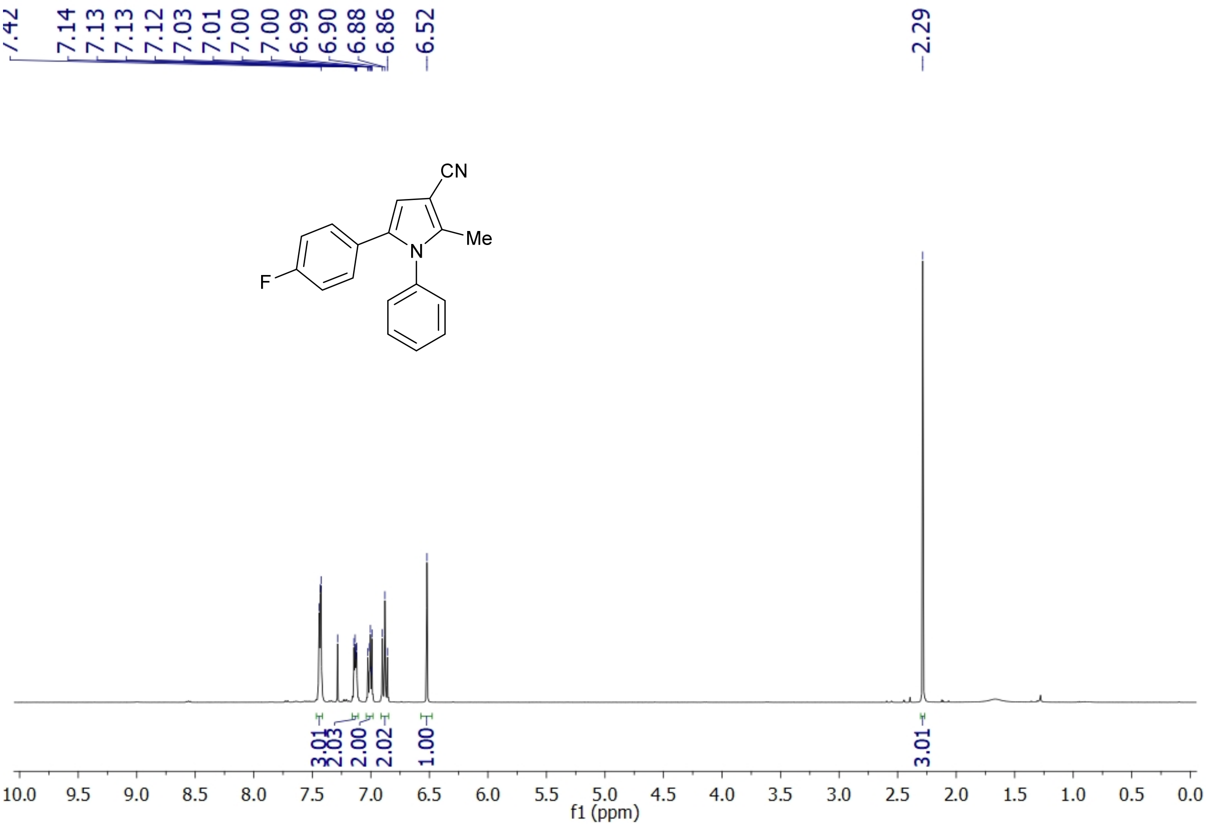
7.14
7.13
7.13
7.12
7.03
7.01
7.00
7.00
6.99
6.90
6.88
6.86
6.52
2.29
CN
Me
N
F
3.01
3.03
2.00
2.02
1.00
3.01
10.0 9.5 9.0 8.5 8.0 7.5 7.0 6.5 6.0 5.5 5.0 4.5 4.0 3.5 3.0 2.5 2.0 1.5 1.0 0.5 0.0
f1 (ppm)

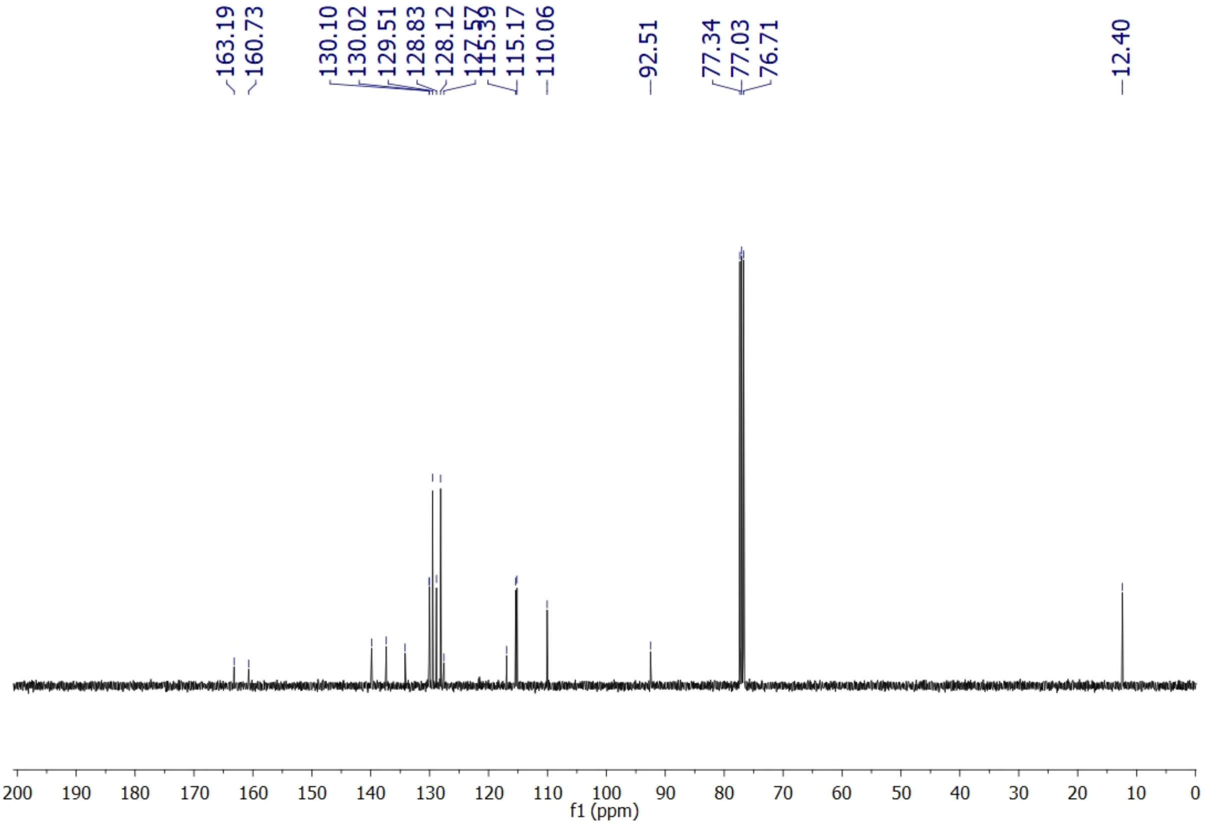
163.19
160.73
130.10
130.02
129.51
128.83
128.12
127.57
115.39
115.17
110.06
92.51
77.34
77.03
76.71
12.40
200 190 180 170 160 150 140 130 120 110 100 90 80 70 60 50 40 30 20 10 0
f1 (ppm)

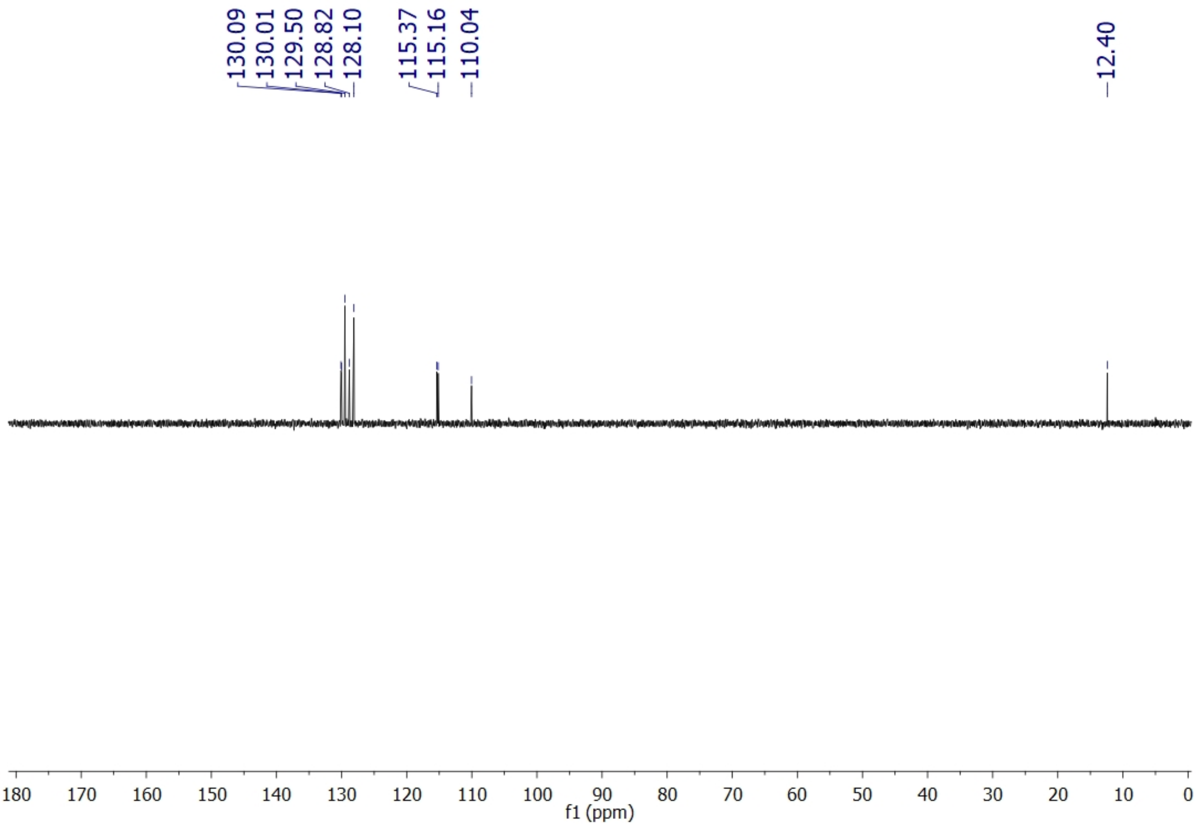

Figure S22. ^{1}H NMR and ^{13}C NMR spectra of 5-(4-Fluorophenyl)-2-methyl-1-phenyl-1*H*-pyrrole-3-carbonitrile.

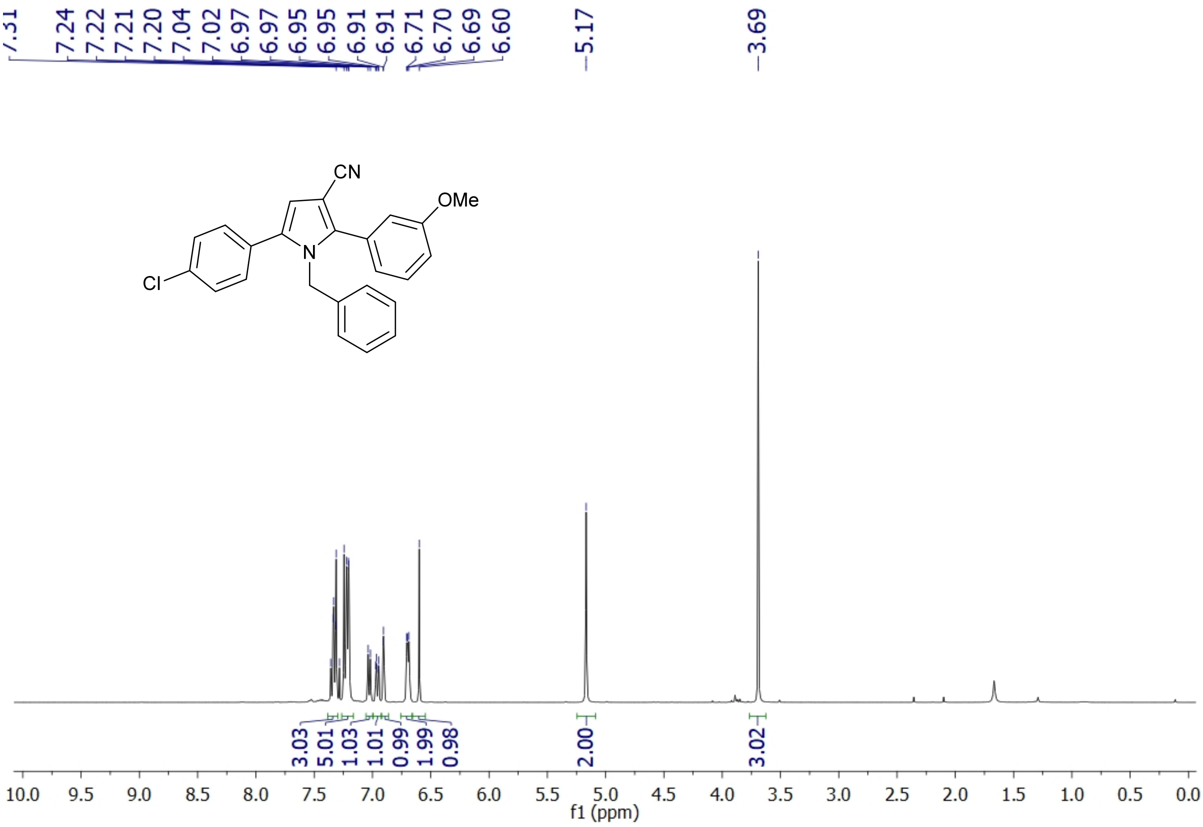

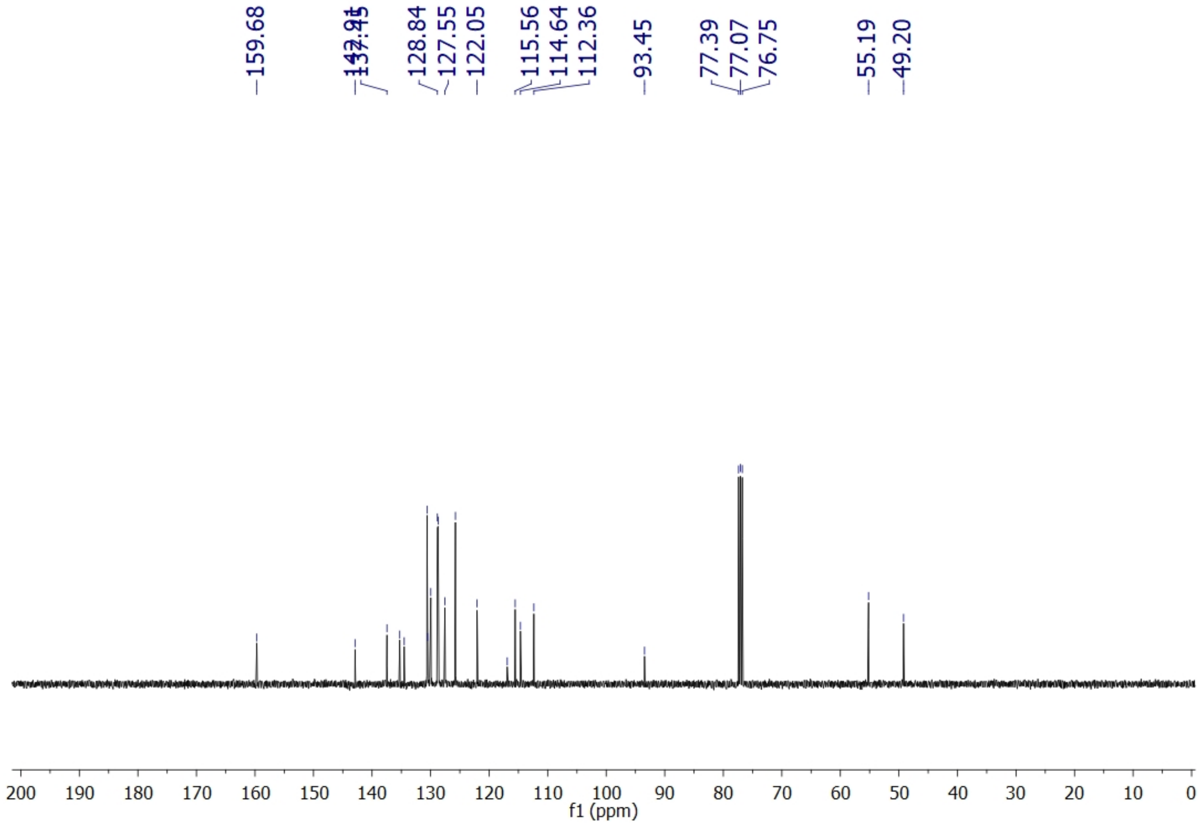

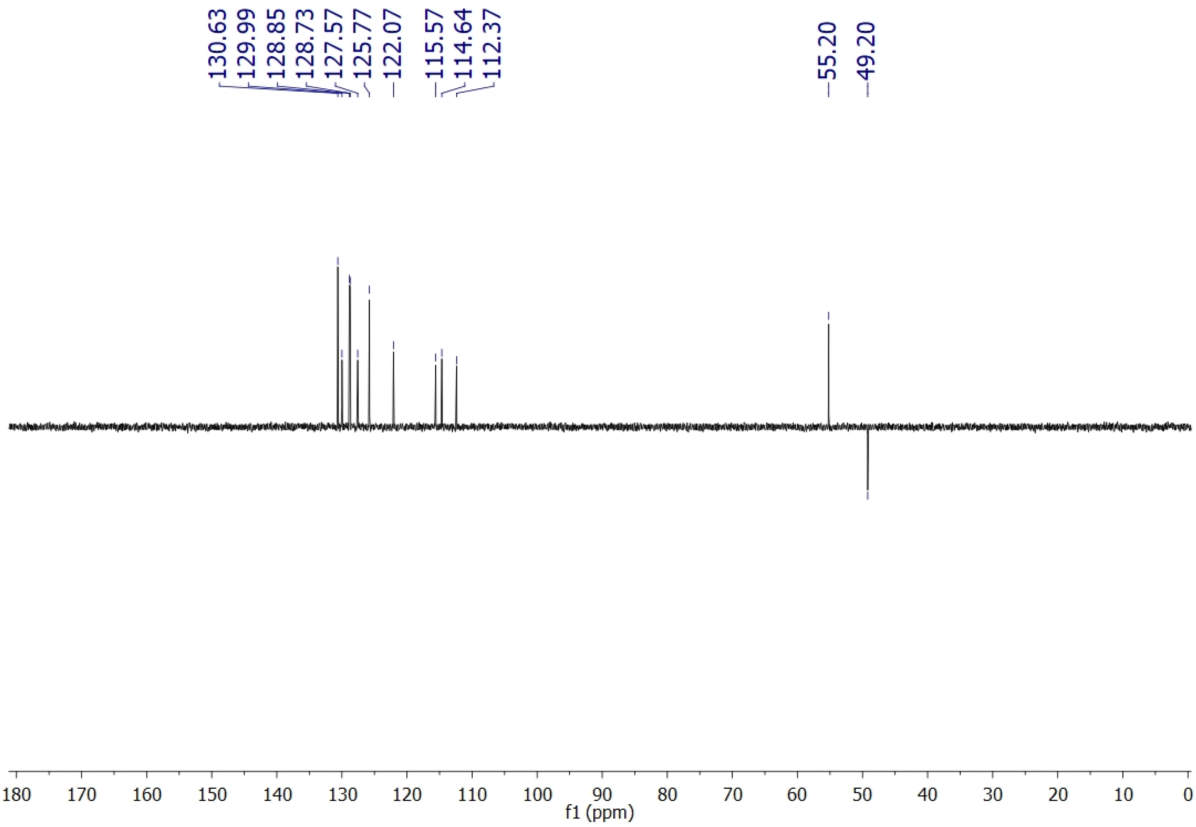

Figure S23. ^{1}H NMR and ^{13}C NMR spectra of 1-Benzyl-5-(4-chlorophenyl)-2-(3-methoxyphenyl)-1*H*-pyrrole-3-carbonitrile.

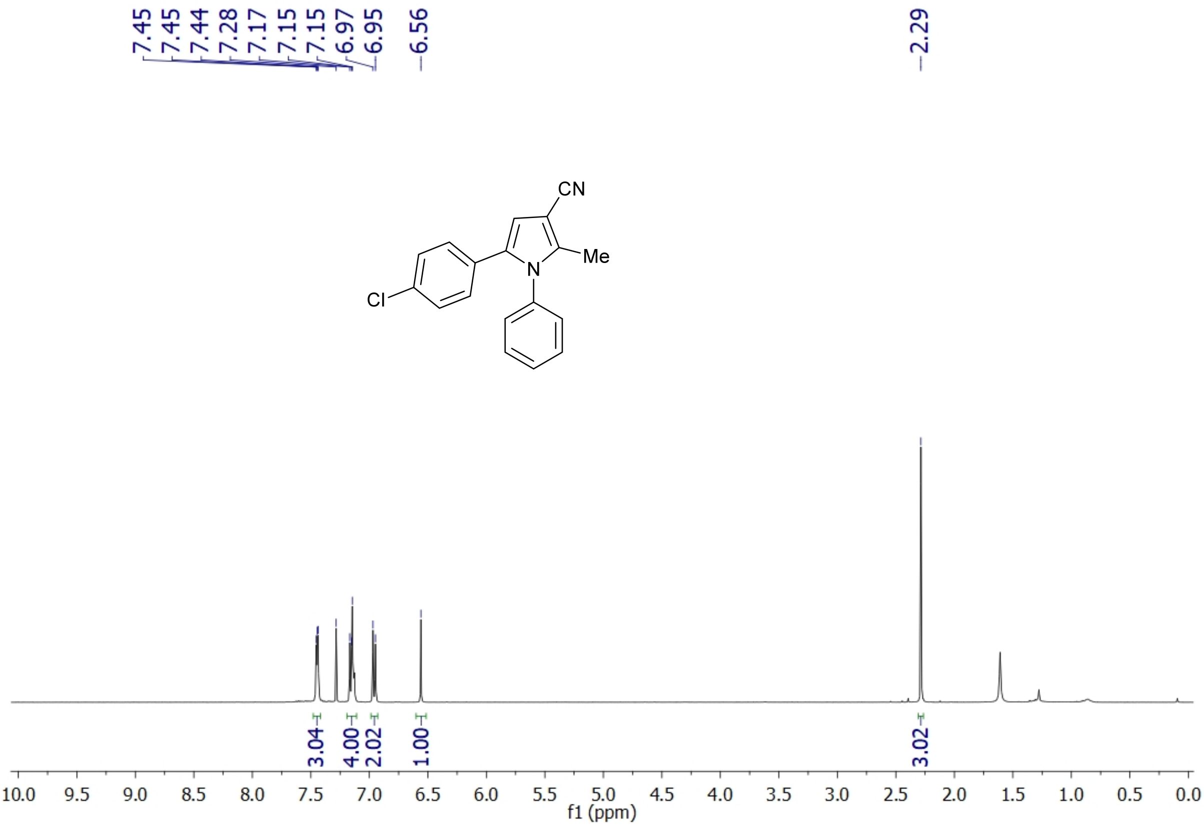
7.45
7.45
7.44
7.28
7.17
7.15
7.15
6.97
6.95
6.56
2.29
CN
Me
N
Cl
3.04
4.00
2.02
1.00
3.02
f1 (ppm)

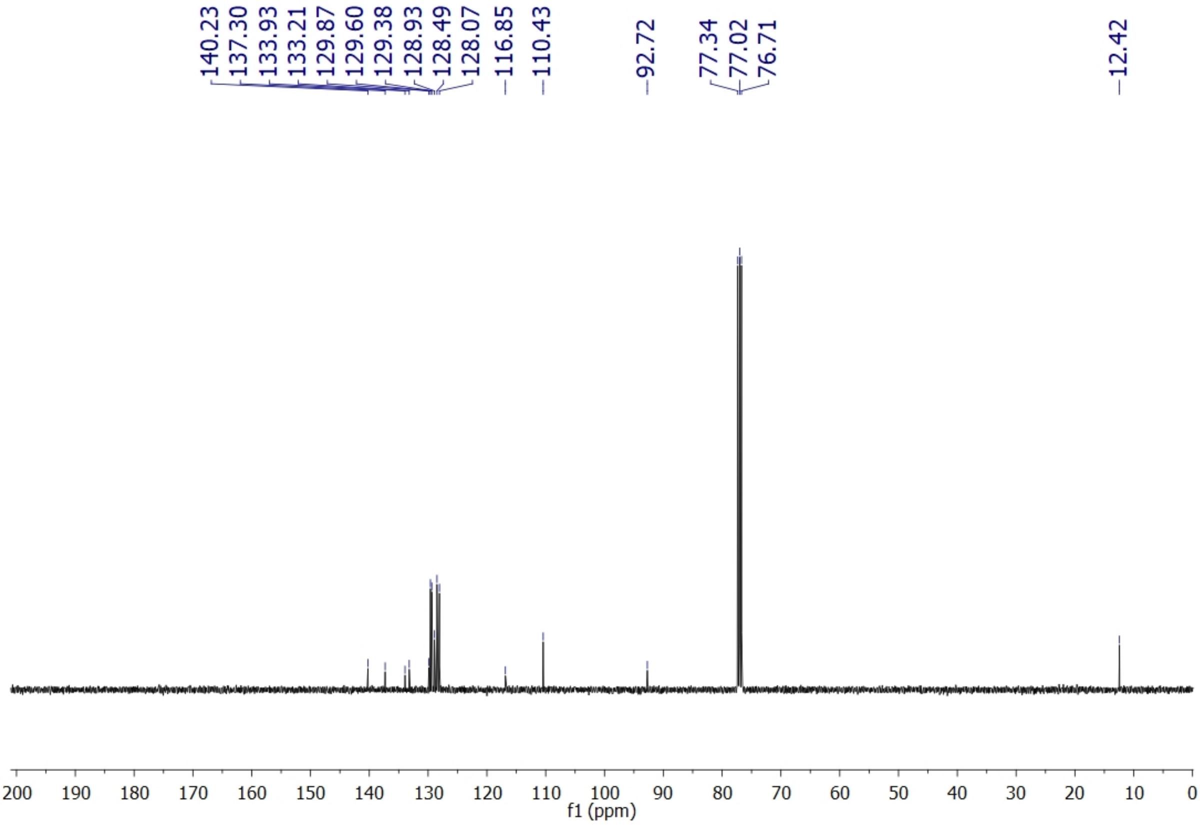
140.23
137.30
133.93
133.21
129.87
129.60
129.38
128.93
128.49
128.07
116.85
110.43
92.72
77.34
77.02
76.71
12.42
f1 (ppm)

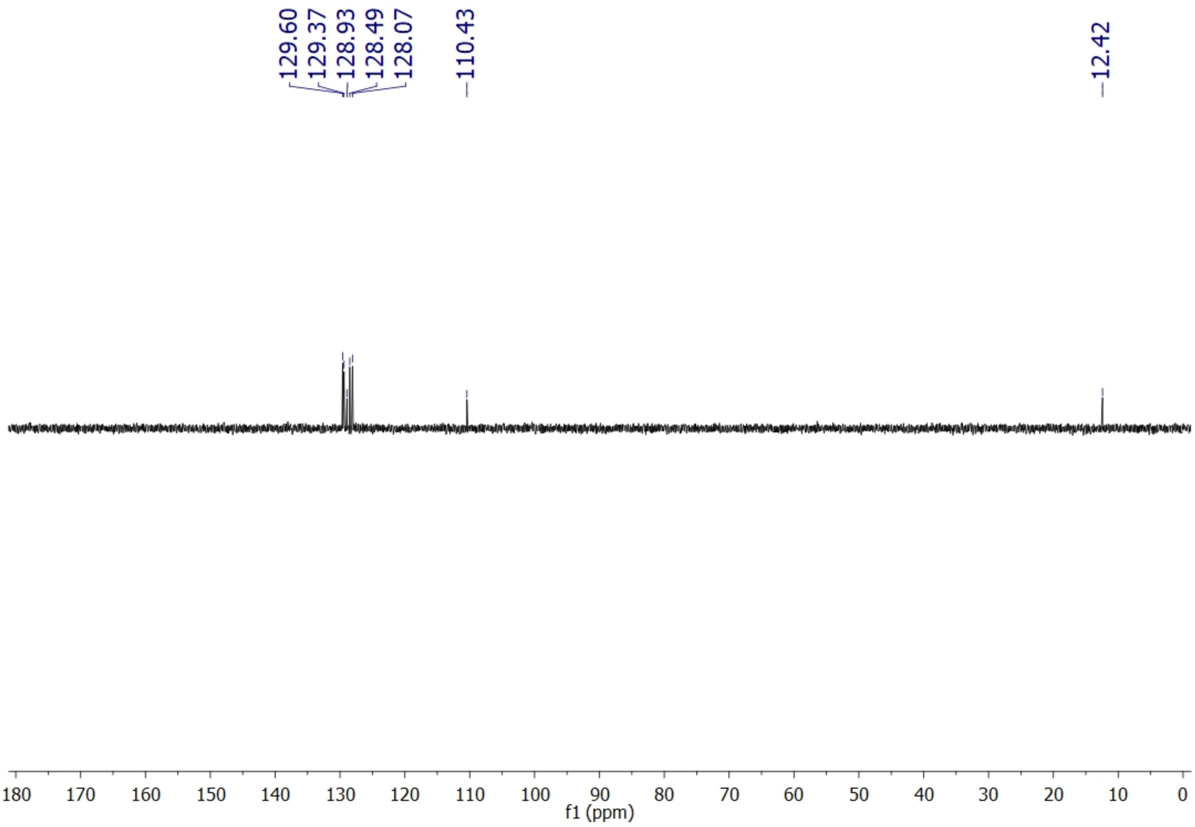

Figure S24. ^1H NMR and ^{13}C NMR spectra of 5-(4-Chlorophenyl)-2-methyl-1-phenyl-1*H*-pyrrole-3-carbonitrile.

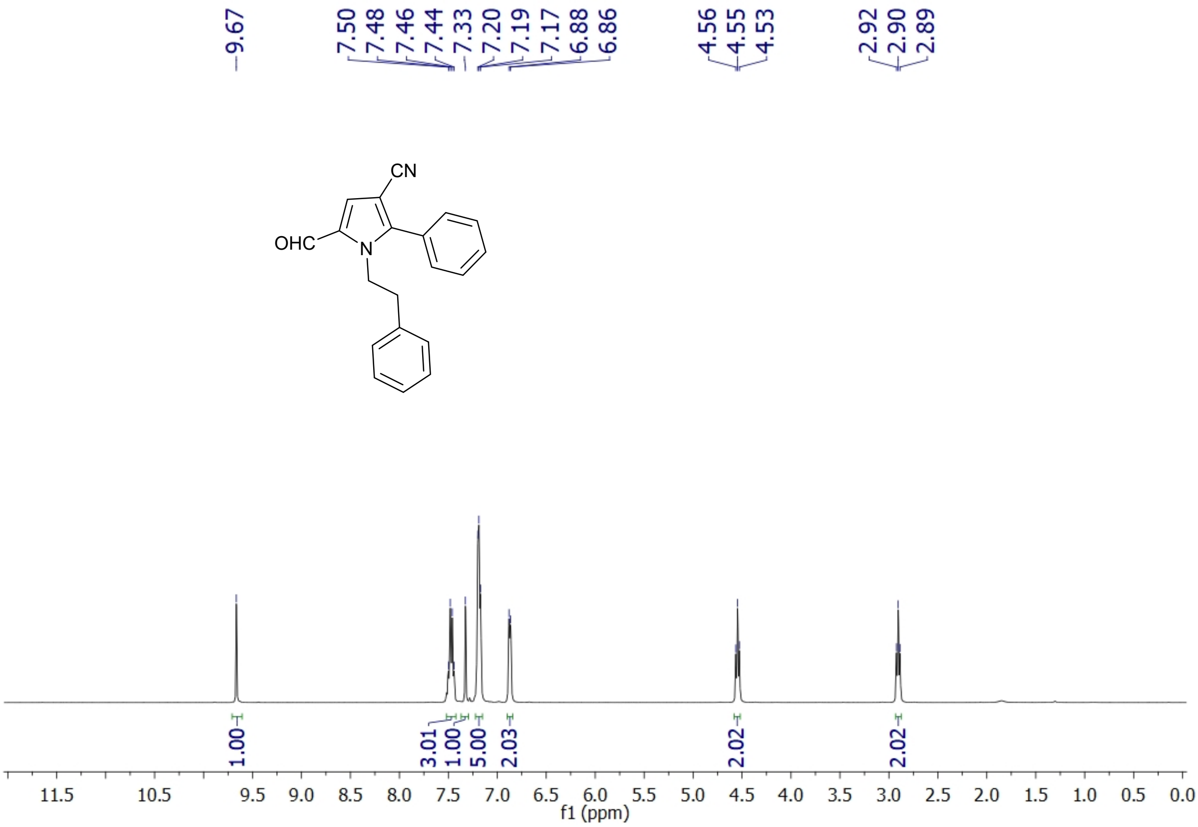
9.67
7.50 7.48 7.46 7.44 7.33 7.20 7.19 7.17 6.88 6.86
4.56 4.55 4.53
2.92 2.90 2.89
CN
OHC
N
1.00
3.01 1.00 5.00 2.03
2.02
2.02
11.5 10.5 9.5 9.0 8.5 8.0 7.5 7.0 6.5 6.0 5.5 5.0 4.5 4.0 3.5 3.0 2.5 2.0 1.5 1.0 0.5 0.0
f1 (ppm)

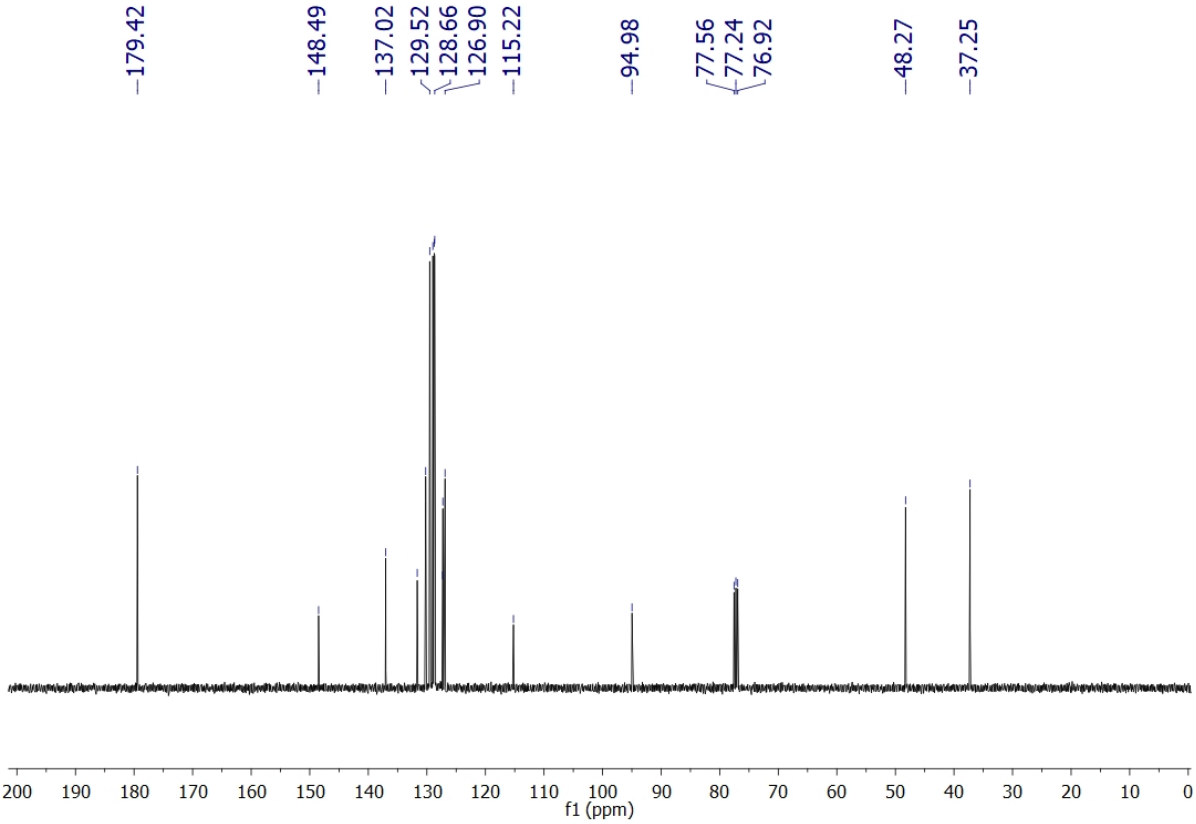
179.42
148.49
137.02 129.52 128.66 126.90 115.22
94.98
77.56 77.24 76.92
48.27
37.25
200 190 180 170 160 150 140 130 120 110 100 90 80 70 60 50 40 30 20 10 0
f1 (ppm)

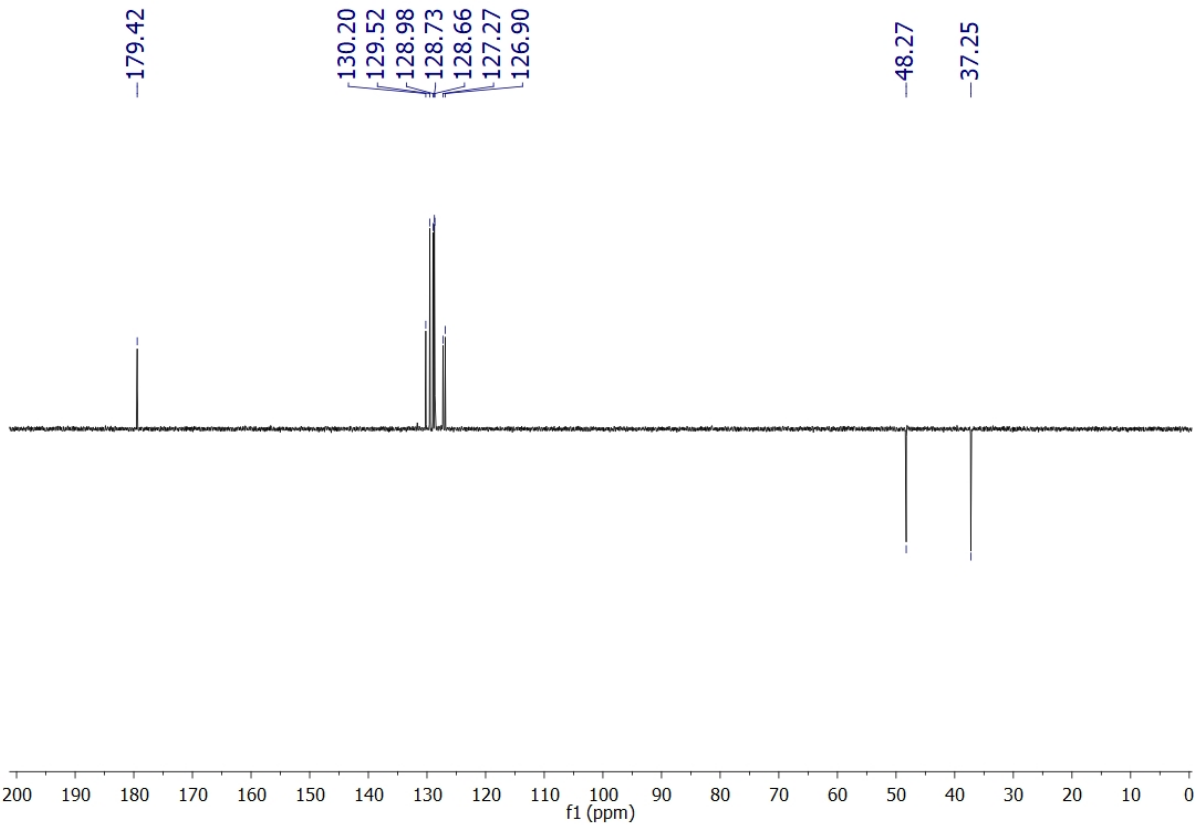

Figure S25. ^{1}H NMR and ^{13}C NMR spectra of 5-Formyl-1-phenethyl-2-phenyl-1*H*-pyrrole-3-carbonitrile

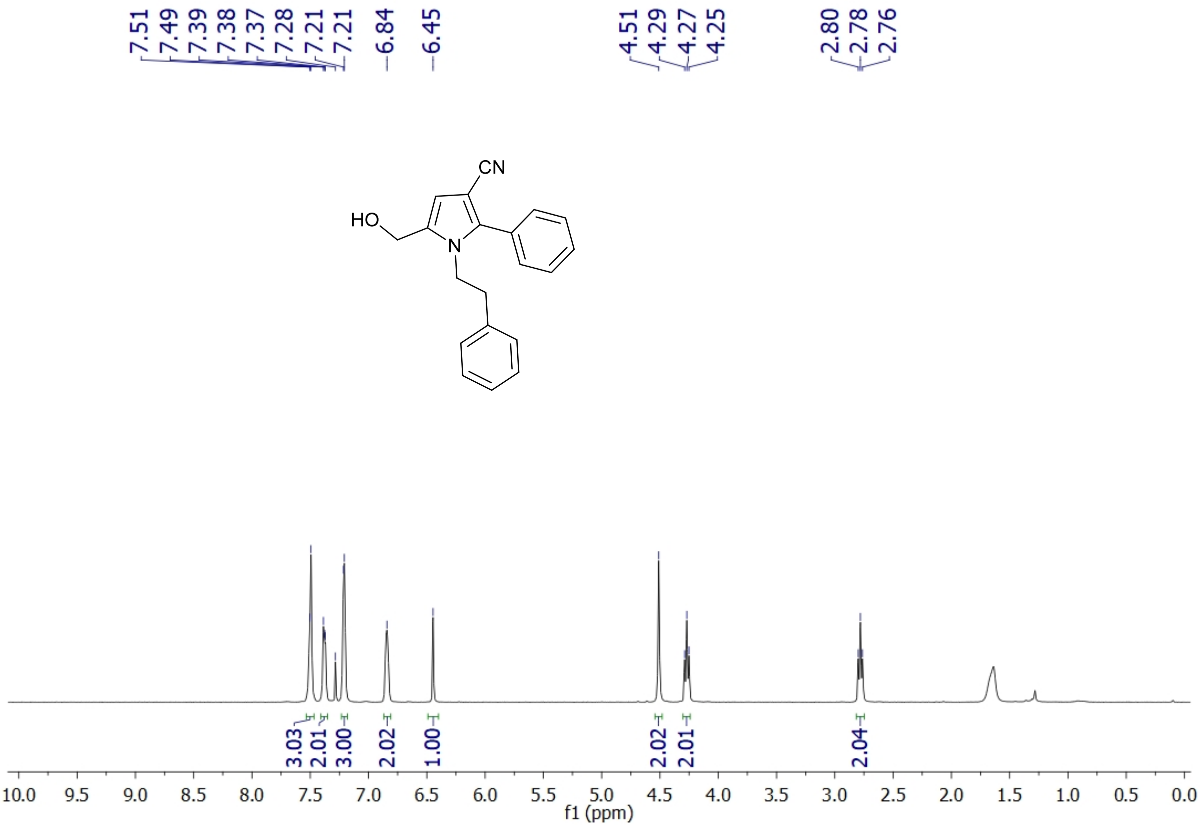
7.51
7.49
7.39
7.38
7.37
7.28
7.21
7.21
6.84
6.45
4.51
4.29
4.27
4.25
2.80
2.78
2.76
CN
HO
N
3.03
2.01
3.00
2.02
1.00
2.02
2.01
2.04
f1 (ppm)

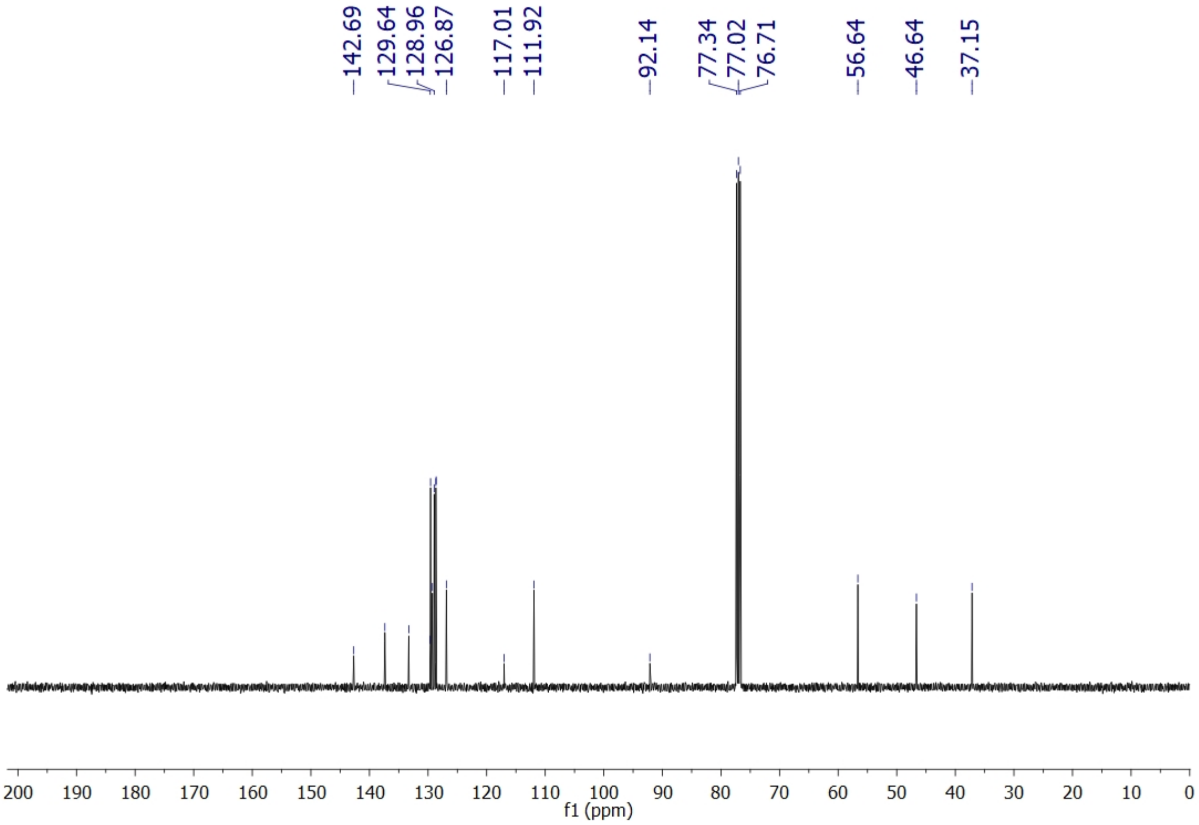
142.69
129.64
128.96
126.87
117.01
111.92
92.14
77.34
77.02
76.71
56.64
46.64
37.15
f1 (ppm)

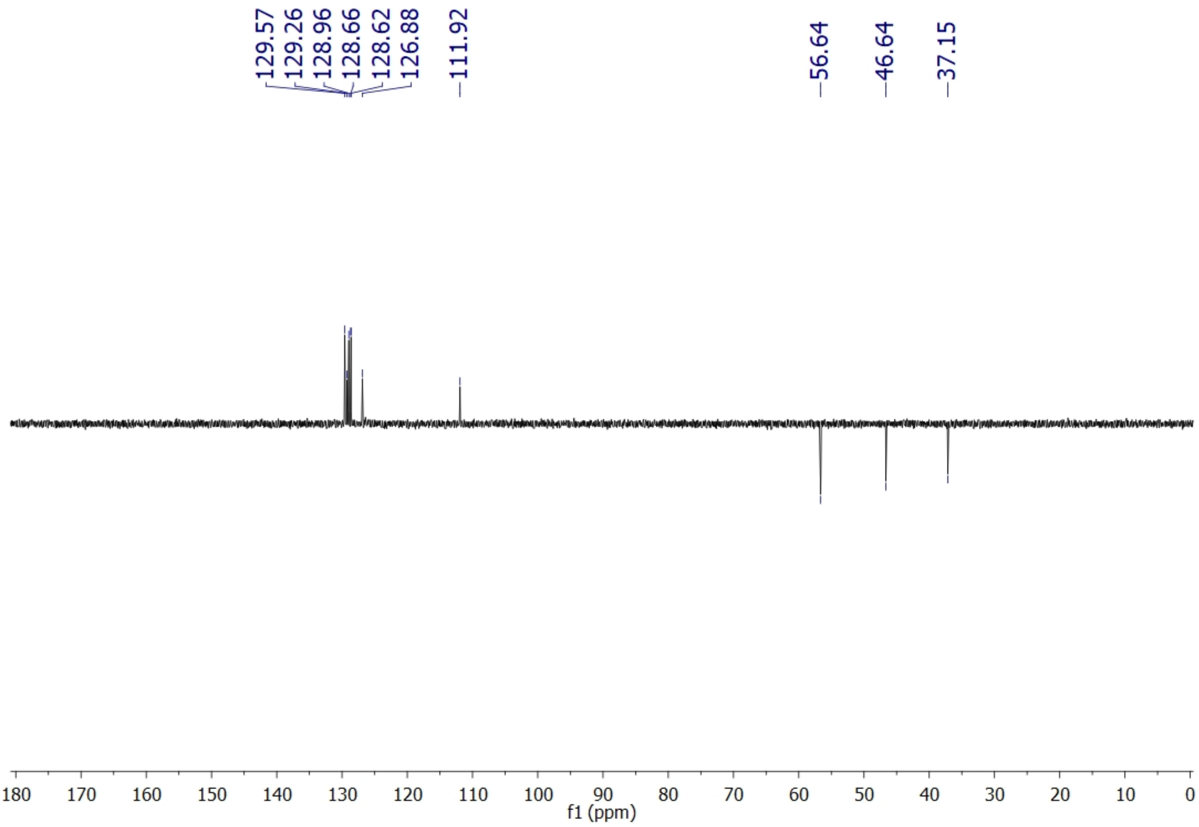

Figure S26. ^{1}H NMR and ^{13}C NMR spectra of 5-(Hydroxymethyl)-1-phenethyl-2-phenyl-1*H*-pyrrole-3-carbonitrile.

2. Single-crystal X-ray and crystal parameters of pyrroles 1c and 14c

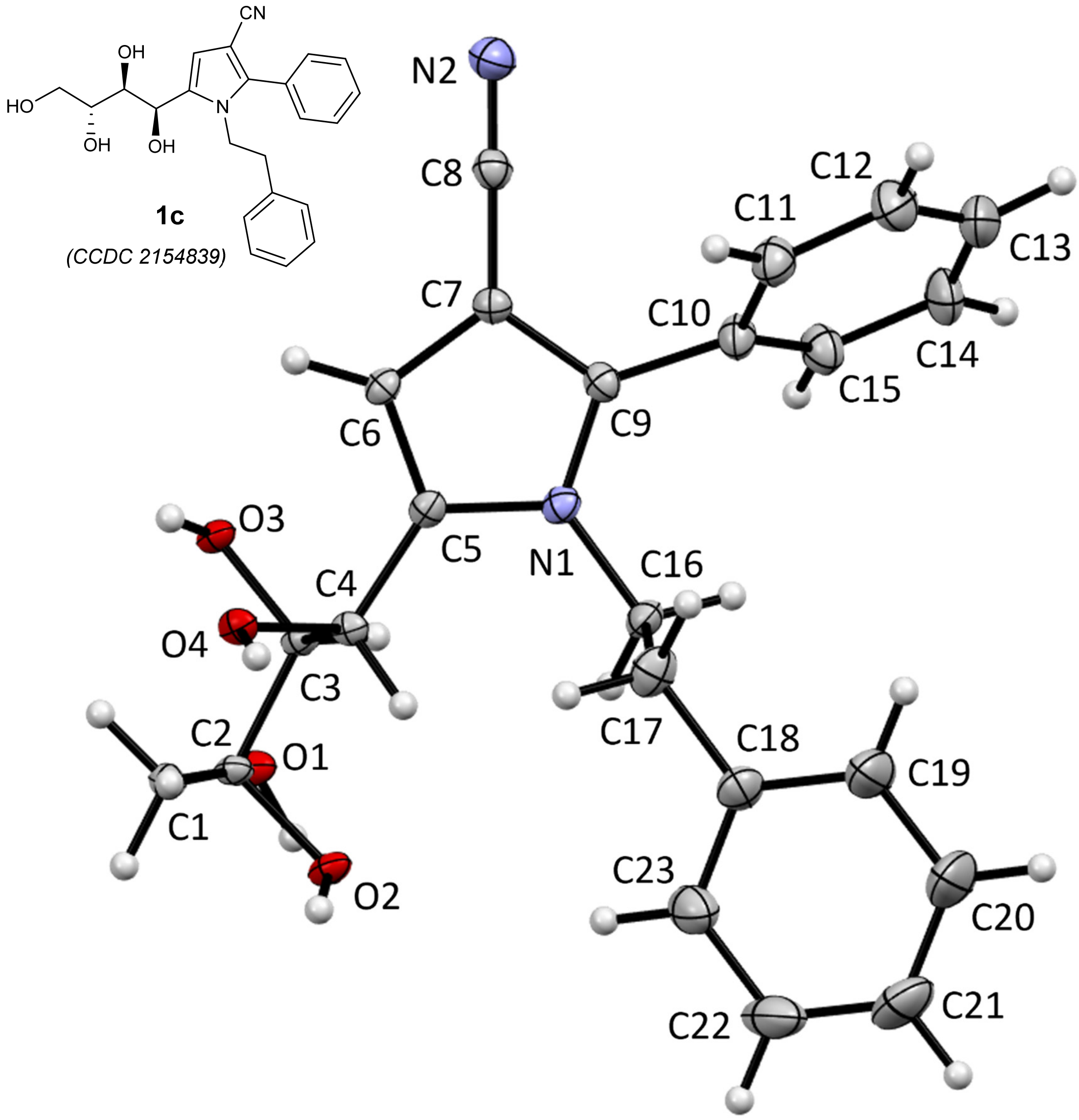

Figure S27. Single-crystal X-ray and crystal parameters of pyrroles **1c** (displacement ellipsoids are drawn at the 50% probability level).

Table S1. Crystal data and structure refinement for product **1c** (CCDC 2154839)

Empirical formula	C23 H21 Cl N2
Formula weight	360.87
Temperature	100(2) K
Crystal system	Triclinic
Space group	P-1
Unit cell dimensions	a = 6.817(2) Å a = 85.965(9)°.<br>b = 10.051(3) Å b = 82.295(9)°.<br>c = 13.998(4) Å g = 86.642(8)°.
Volume	946.9(5) Å^3
Z	2
Density (calculated)	1.266 Mg/m^3
F(000)	380
Reflections collected	11937
Independent reflections	3622 [R(int) = 0.1102]
Max. and min. transmission	1.00 and 0.52
Goodness-of-fit on F^2	1.120
Final R indices [I>2sigma(I)]	R1 = 0.0877, wR2 = 0.1689
R indices (all data)	R1 = 0.1546, wR2 = 0.1965

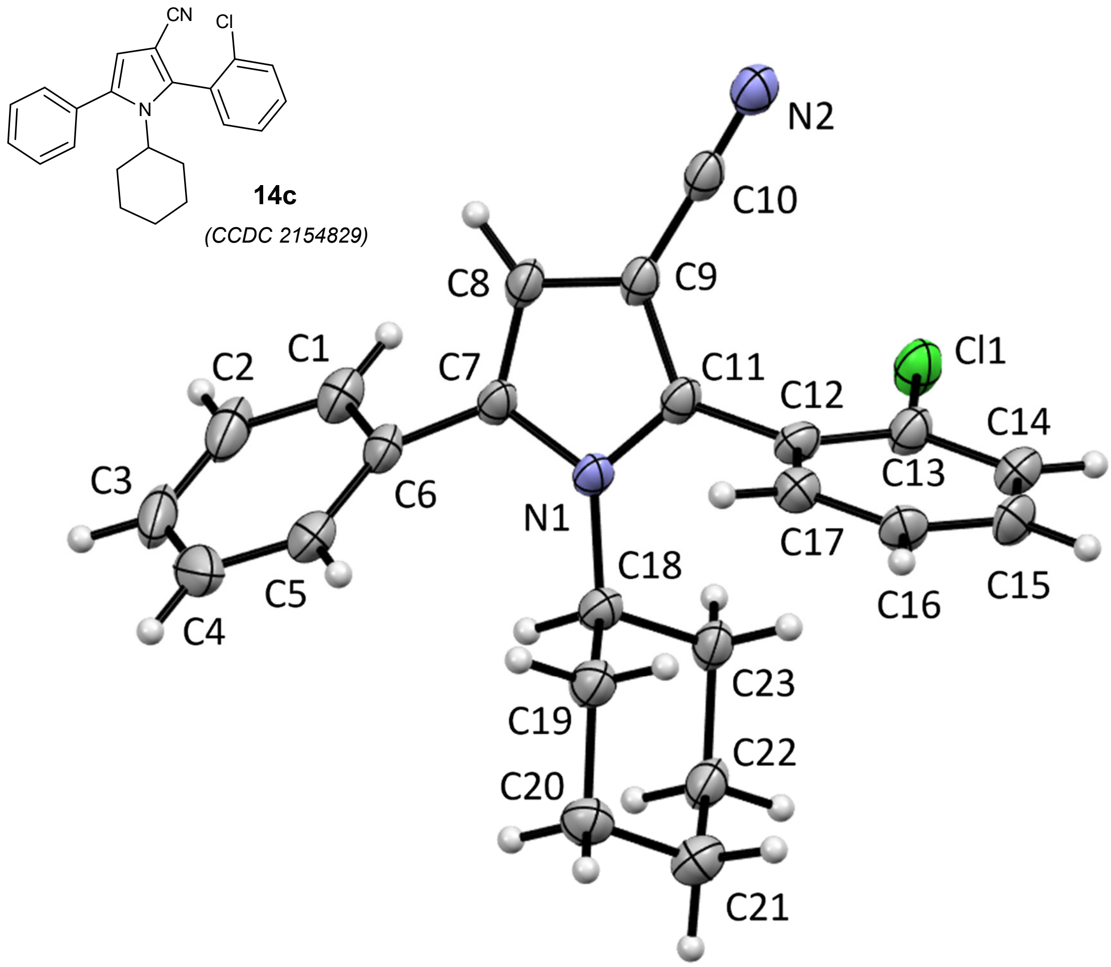

Figure S28. Single-crystal X-ray and crystal parameters of pyrroles **1c** (displacement ellipsoids are drawn at the 50% probability level).

Table S2. Crystal data and structure refinement for product **14c** (CCDC 2154829)

Empirical formula	C23 H24 N2 O4
Formula weight	392.44
Temperature	100(2) K
Crystal system	Triclinic
Space group	P-1
Unit cell dimensions	a = 9.5794(9) Å a= 78.127(4)°.<br>b = 12.2157(13) Å b= 83.884(4)°.<br>c = 17.948(2) Å g = 89.967(4)°.
Volume	2043.1(4) $Å^3$
Z	4
Density (calculated)	1.276 Mg/m^3
F(000)	832
Reflections collected	159527
Independent reflections	29865 [R(int) = 0.0785]
Max. and min. transmission	0.99 and 0.88
Goodness-of-fit on F^2	1.098
Final R indices [I>2sigma(I)]	R1 = 0.0852, wR2 = 0.2133
R indices (all data)	R1 = 0.1186, wR2 = 0.2370

3. 2D NMR of product 14b

COSY

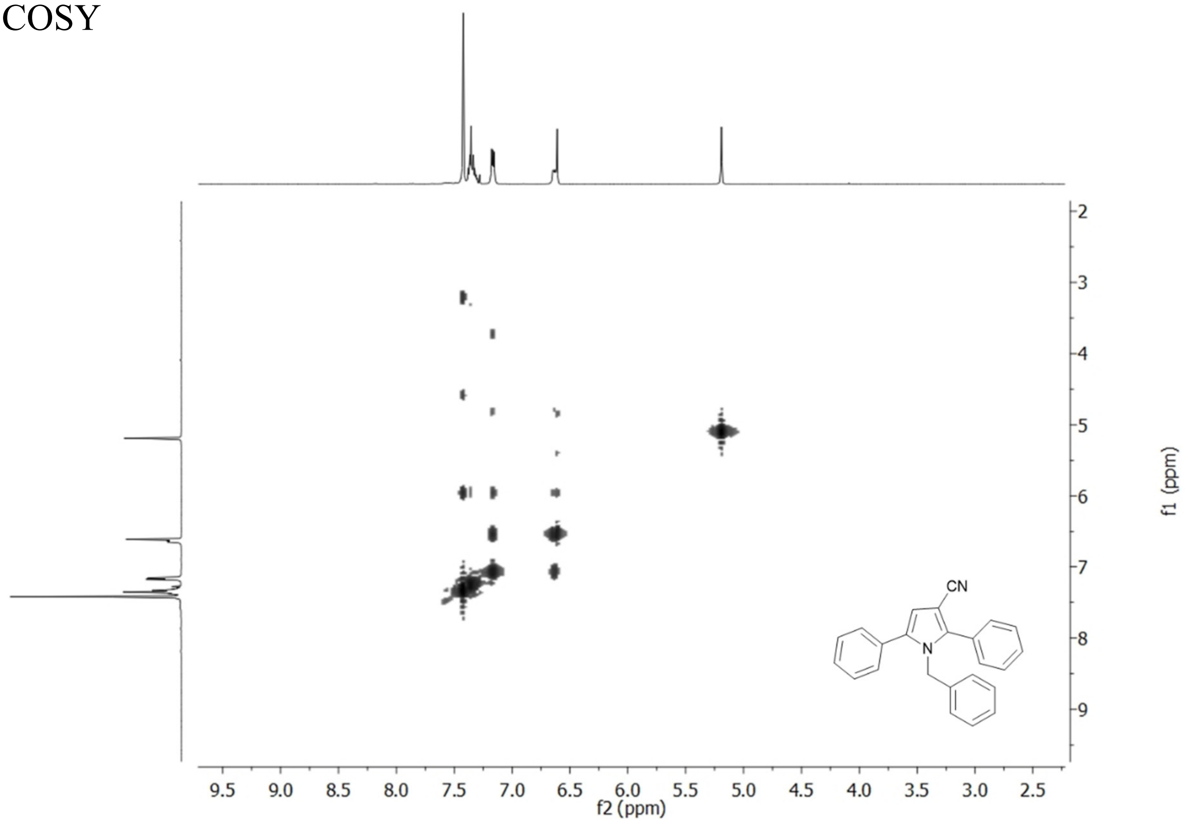

HSQC

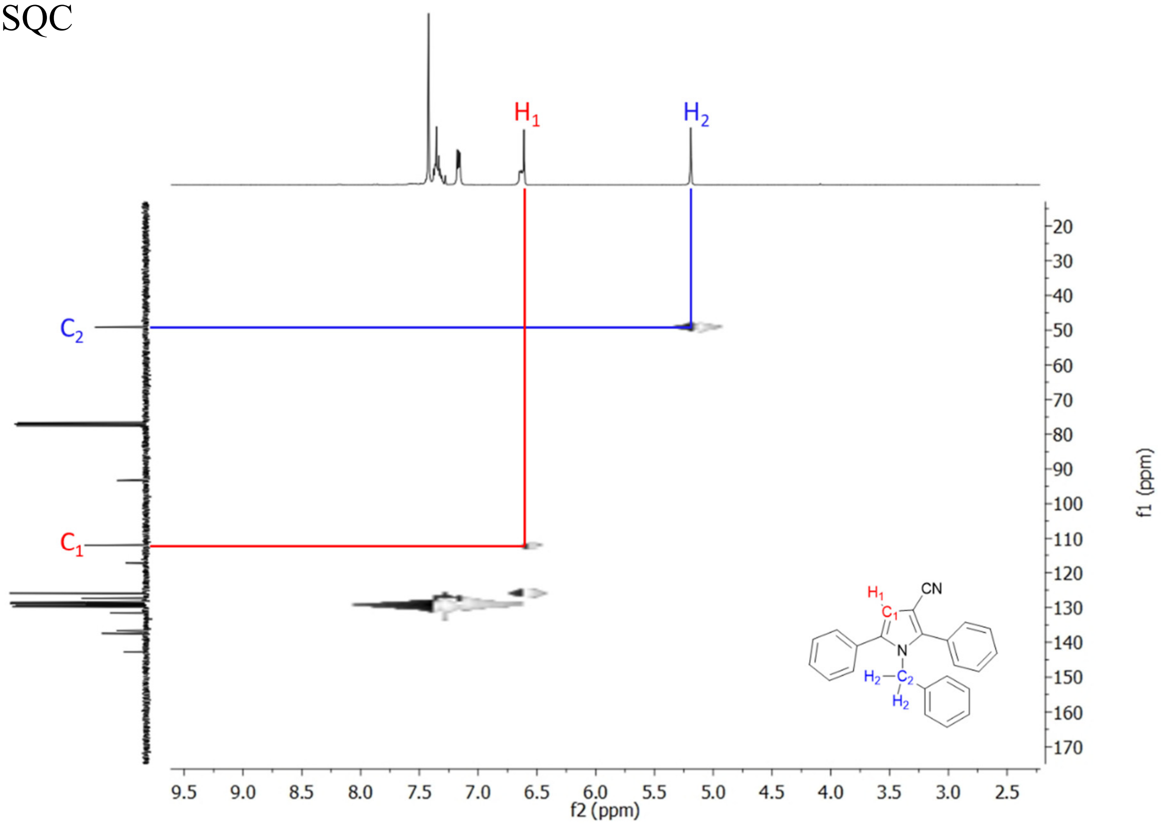

HMBC

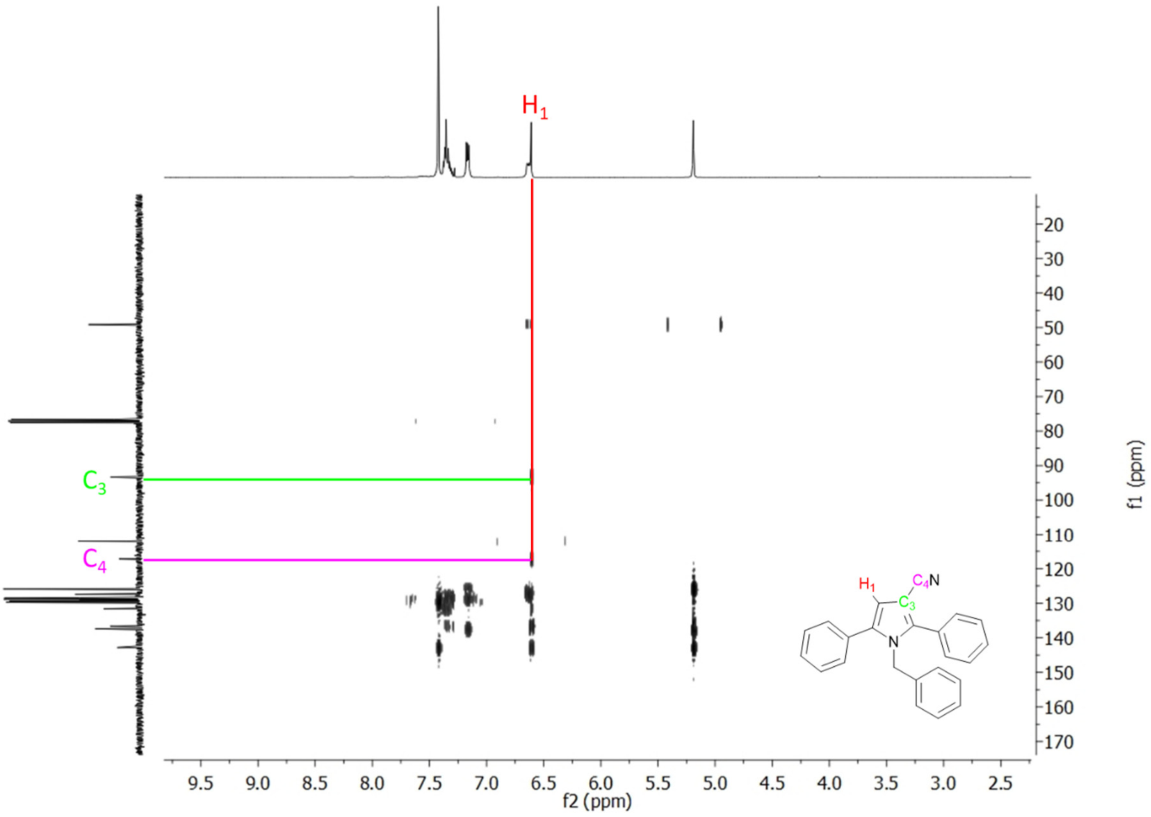

Figure S29. 2D NMR of product **14b** (COZY, HSQC, HMBC).
